# New Stable Cu(I) Catalyst Supported on Weakly Acidic Polyacrylate Resin for Green C-N Coupling: Synthesis of *N*-(Pyridin-4-yl)benzene Amines and *N*,*N*-Bis(pyridine-4-yl)benzene Amines

**DOI:** 10.3390/molecules22010002

**Published:** 2016-12-22

**Authors:** Nitin Kore, Pavel Pazdera

**Affiliations:** Centre for Syntheses at Sustainable Conditions and their Management, Department of Chemistry, Faculty of Science, Masaryk University, Kamenice 5, CZ-62500 Brno, Czech Republic; kore_ns@yahoo.co.in

**Keywords:** polymer solid support, supported Cu(I) catalyst, C-N coupling, recyclable green catalyst

## Abstract

A method for preparation of a new stable Cu(I) catalyst supported on weakly acidic polyacrylate resin without additional stabilizing ligands is described. A simple and efficient methodology for Ullmann Cu(I) catalyzed C-N cross coupling reactions using this original catalyst is reported. Coupling reactions of 4-chloropyridinium chloride with anilines containing electron donating (EDG) or electron withdrawing (EWG) groups, naphthalen-2-amine and piperazine, respectively, are successfully demonstrated.

## 1. Introduction

Synthetic applications of copper(I)-catalyzed post-Ullmann-type coupling syntheses have been well known for decades, however, they have remained relatively limited because of the required harsh reaction conditions involving high temperatures, highly polar solvents, use of further ligands and use of stoichiometric amounts of copper(I) reagents. Several studies were reported in the literature for post Ullmann-type methods, e.g., the *N*-arylation of anilines [1,2], imidazoles [3], amides [4], nitrogen containing heterocycles [5] and hydrazides [6] as well. However, all above reported C-N coupling reactions are homogeneously catalyzed and carried out in the presence of ligands, which are necessary for stabilization of both possible oxidation states of the copper intermediates, i.e., Cu(I) and Cu(III).

In some articles, uses of cuprous oxide [7,8], copper(I) halide salts [9], CuO nanoparticles [10,11] and silica supported copper(I) complex [12] for *N*-arylation reaction as heterogeneous catalysts are described. However, these methodologies involve high temperatures as well as highly polar solvents (DMF).

Arylaminopyridine moiety in molecules is useful in biological and pharmaceutical science [13]. Syntheses of compounds with aminopyridine moiety are mainly reported as C-N cross coupling reactions of amino pyridine with activated aryl halide [14,15] catalyzed by Pd(0) complexes in polar solvent such as DMF, 1,4-dioxane etc. C-N coupling of aryl amines with 4-chloropyridin-1-ium chloride using Pd(0) catalyst was reported by Keddie et al. [16]. No reports are available on C-N cross coupling of aryl amines with 4-chloropyridine using the Ullmann copper catalysis yet.

Cation exchange resins are easily available and used for different purposes in industry as well as in laboratory. Use of cation exchange resins as a support for deposition of charged metal ion and metal complexes for different catalytic reactions were studied [17,18,19,20]. Modified cation exchange resin is easily separable from reaction mixture compared to other solid supports such as silica, alumina, carbon, zeolite, etc. due to its larger size and, simultaneously, the resin, unlike hard supports, does not rub against the surface of reaction vessels.

The goal of our work is to demonstrate catalytic application of bare Cu(I) ion supported on weakly acidic polyacrylate resin at mild reaction conditions. Herein, we report the method for preparation of this supported catalyst as a catalyst for C-N cross coupling reactions of different amines with 4-chloropyridine in the absence of ligands for stabilization of possible oxidation states of the copper intermediates.

## 2. Results and Discussion

The selective reduction methods of Cu(II) to Cu(I) may be carried out by action of iodide ion, cyanide ion [21], or using sodium ascorbate [22]. Few articles demonstrate the use of hydroxyl amine as a reducing agent for reduction of copper(II) complex [23], reduction of copper(II) sulfate [24,25] and to reduce Cu(II) ion [26] to corresponding Cu(I). In this work, we used hydroxyl ammonium chloride successfully to reduce polymer supported Cu(II) ion in ammine complex form to Cu(I).

We found that obtained supported Cu(I) catalyst is very stable against oxidation by air oxygen due to stabilization of Cu(I) ions probably by flexible coordination in the field of carboxylate functional groups present in the weakly acidic macroporous cation exchange resin.

We decided to try the coupling reaction of 4-methoxyaniline with 4-chloropyridine catalyzed by title supported Cu(I) catalyst as a model reaction, because of nucleophilic properties of 4-methoxyaniline and, moreover, their coupling product prepared by Pd(0) catalyzed reaction with result of good yield was reported previously [16] (Table 1).

We were pleased to find that the reaction occurred to afford *N*-4-pyridyl(*p*-methoxyphenyl)amine in 64% yield in presence of Cu(I) ions supported on weakly acidic cation-exchanger resin as a catalyst. However, the reaction using CuI as a catalyst affords only 8% of product and in the case of process without catalyst there was no reaction at all at the same reaction conditions. We suppose that the presence of sufficient amount of carboxylate moieties on resin stabilizes Cu(I) catalyst, hence it shows much better activity than only CuI. 

**Scheme 1:**
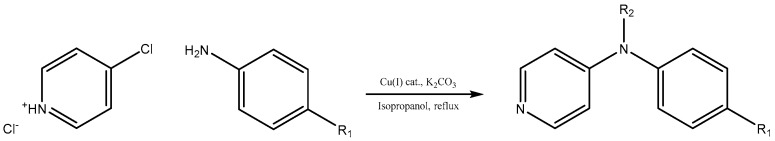
C-N coupling reaction with 4-chloropyridin-1-ium chloride with substituted aniline. R_1_–H/EDG/EWG; R_2_–H/Pyridine.

To investigate the further scope of the procedure, we attempted to carry out the reaction with a broader spectrum of different aromatic amines (Scheme 1). The temperature of boiling isopropyl alcohol as well as the presence of potassium carbonate or hydrogen carbonate as a base in the reaction mixture evoked decomposition of 4-chloropyridine after 24 h during the course of synthesis, hence duration of all reactions was kept for 24 h. We successfully recycled the title Cu(I) catalyst twenty times for C-N coupling of 4-methoxyaniline with 4-chloropyridine without losing any activity (see Supplementary Materials). TON (turnover number) was calculated as a ratio of mole of 4-chloropyridin-1-ium chloride converted to product and mole of Cu present in catalyst.

The C-N coupling of 4-chloropyridine with anilines containing electron donating substituents, i.e., 4-*N*,*N*-diethylbenzene-1,4-diamine, 4-*N*,*N*-dimethylbenzene-1,4-diamine, 4-ethoxy-, or 4-methoxyanilinem afforded products with the only one amino group hydrogen atom replaced in 64%–80% yield (Table 2, entries 1–2, 4–5).

The synthetic process with sterically hindered o-substituted aniline, e.g., 2,5-dimethoxy- and 2,4-dimethylaniline, underwent replacement of one amine hydrogen atom in 22% and 12% yield, respectively. Low yields of the reactions may be inflicted by steric hindrance of *o*-substitutes in the molecule (Table 2, entries 3, 6).

The reaction with slightly deactivated substituted aniline, 4-chloro-, 4-bromo-, 4-iodo-, 4-fluoroaniline, and ethyl 4-aminobenzoate, underwent reaction with replacement of one amine hydrogen in 26%–49% yield (Table 2, entries 8–12).

Our observation of formation of C-N coupling product under replacement of only one hydrogen atom of aniline amino group in high yield in the case of aniline consisting electron donating group and formation of C-N coupling product with replacement of the only hydrogen of aniline in comparatively low yield concerning aniline consisting slightly deactivating group is similar to all previously reported articles [6,10,15].

The reaction with aniline substituted by a strong electron withdrawing group, 2,4-dichloro, 4-trifluoromethyl, 4-cyano, 2-cyano, 3-nitro- and 4-nitroaniline, ended up in formation of novel *N*,*N*-bis(pyridin-4-yl)benzene amines as a major product in 24%–42% yield and *N*-(pyridin-4-yl)benzene amines as the second minor product in 6%–9% yield. (Table 2, entries 13–14, 16–19).

The reaction with 2,5-dichloroaniline gave selectively the corresponding *N*,*N*-bis(pyridin-4-yl)benzene amine only.

**Figure 1:**
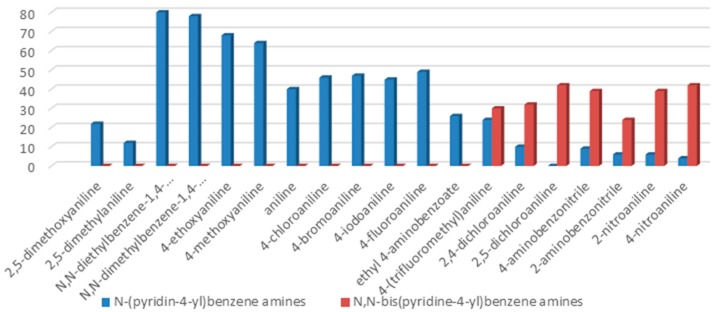
Comparison of yields for different aromatic amines in C-N coupling reaction with 4-chloropyridine in the presence of K_2_CO_3_ as a base.

The reaction with naphthalene-2-amine (Table 2, entry 20) was carried out with a high yield; on the other hand, the reaction with naphthalene-1-amine gave trace amount of expected product, which we were not able to purify. C-N cross coupling gave two types of products, i.e., *N*-(pyridin-4-yl)benzene amines and/or *N*,*N*-bis(pyridine-4-yl)benzene amines depends on electronic effect on aniline (Figure 1).

In the case of coupling reaction between piperazine and 4-chloropyridinium chloride, a molar excess of piperazine as a base and isopropyl alcohol as a solvent were used. Only formation of the 1-(pyridin-4-yl)piperazine in very good yield was observed. If the reaction was carried out under reflux in methanol or in ethanol, 4-methoxy- or 4-ethoxypyridine was formed due to competing reaction in yield 22% and 18%, respectively. When using isopropyl alcohol, the above-mentioned competitive C-O coupling reaction did not proceed at all apparently because of the presence of the bulky isopropyl group and the low acidity of hydroxyl group. On the other hand, the application of potassium hydroxide, carbonate or hydrogen carbonate as a base as well as temperature of boiling solvent evoked decomposition of 4-chloropyridine in the course of synthesis.

**Figure 2:**
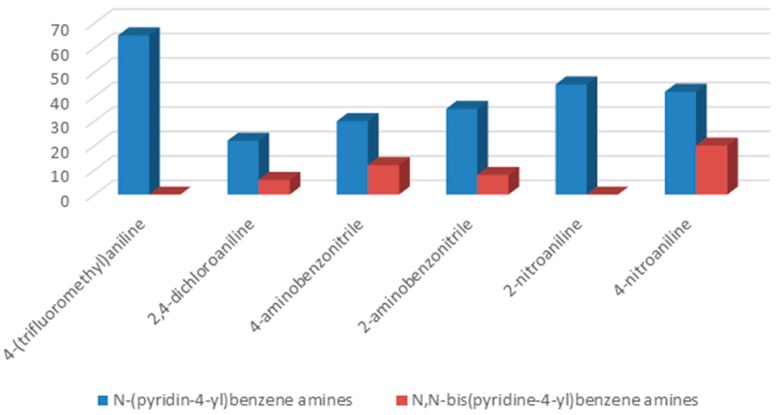
Comparison of yields for different aromatic amines in C-N coupling reaction with 4-chloropyridine in the presence of KHCO_3_ as a base.

To understand the chemistry of the coupling process, we decided to accomplish the same reaction using KHCO_3_ as a weaker base than potassium carbonate. In the presence of KHCO_3_ as a base, 4-(trifluoromethyl)aniline as well as 2-aminobenzonitrile yielded selectively corresponding *N*-(pyridin-4-yl)benzene amines as the only product, while, in the cases of 2-nitroaniline, 2,4-dichloroaniline, 4-aminobenzonitrile, and 4-nitroaniline, formation of *N*-(pyridin-4-yl)benzene amines as a major product together with *N*,*N*-bis(pyridin-4-yl)benzeneamines as a secondary product was observed (Figure 2).

To understand structural behavior of 4-chloropyridine derivative, we attempted to carry out reaction of 4-methoxyaniline with 4-chloro-1-methylpyridinium iodide and 4-chloro-3,5-dimethylpyridine.

There was no reaction between 4-methoxyaniline and 4-chloro-3,5-dimethylpyridine observed. This can be explained by big sterical hindrance among two methyl groups and a nearby chlorine atom. This steric hindrance can be unfavorable to oxidative addition step (Table 3, entry 2). 4-Chloro-1-methylpyridinium iodide underwent reaction with 4-methoxyaniline under replacement of one hydrogen atom of amino group with 80% yield (Table 3, entry 3). The presence of quaternized pyridine nitrogen in 4-chloro-1-methylpyridinium iodide activates chlorine atom for exchange, hence it shows better activity than 4-chloropyridine.

## 3. Materials and Methods

All reagents were purchased from commercial suppliers (Sigma-Aldrich, Merck, Acros Organics, Diegem, Belgium) and were used without further purification. Purolite^®^ C104 Plus, macroporous weakly acidic cation exchange resin, divinylbenzene crosslinked polyacrylic, total capacity (min) 4.5 eq/L (H^+^-form), shipping weight (approx.) 740–780 g/L [27]. All of the solvents were purified according to the standard methods. The reactions were monitored by TLC (aluminum plates TLC Silica gel 60 F254 by Merck), spots were detected using ninhydrin and/or UV lamp CAMAG. Melting points were measured on Boetius apparatus PHMK 05 (VEB Kombinat Nagema, Dresden, Germany). NMR spectra were obtained from a Bruker Avance NMR III^TM^ 300 MHz and Bruker Avance III™ 500 MHz (Leiderdorp, The Netherland), using wideband probe BBFO. Infrared spectra were collected on Bruker Tensor 27 spectrometer. Samples were measured in the form of KBr pellets. High resolution mass spectra were obtained on Agilent 6224 Accurate-Mass TOF spectrometer (Santa Clara, CA, USA).

### 3.1. Preparation of Catalysts

Purolite^®^ C104 Plus resin in Na^+^ form in amount of 75.0 g was stirred in water (200 mL). Cupric acetate monohydrate (49.9 g, 250 mmol) dissolved in water (750 mL) was mixed with aqueous ammonia solution (28 *w*/*w* %, 85 mL, 1255 mmol) under good stirring. Furthermore, the dark blue solution was added to the resin suspension and stirred for 30 min. Then the aqueous phase was decanted and the blue solid washed twice with water (300 mL). The resin was then stirred for 30 min in the water solution (250 mL) containing hydroxylammonium chloride (29.9 g, 430 mmol) at 50 °C until the blue color of resin changed to light gray. After that, the solution was decanted, the solid residue washed twice with water (250 mL), twice with methanol (150 mL) and dried in vacuum. The copper content determined by flame atomic absorption spectrometry was approximately 2.2 mmol of Cu/1 g of dry catalyst.

### 3.2. General Synthetic Procedure for C-N Coupling of Aryl Amine with 4-Chloropyridin-1-ium Chloride

A mixture of the corresponding aryl amine (9.74 mmol), 4-chloropyridin-1-ium chloride (1.21 g, 8.12 mmol), anhydrous potassium carbonate (3.45 g, 25 mmol) and supported Cu(I) catalyst (100 mg, 0.22 mmol of Cu, 2.7 mol % of Cu) was refluxed in isopropyl alcohol (40 mL) for 24 h under open atmosphere conditions. After 24 h, reaction mixture was filtered to remove potassium salts and catalyst. The solid was stirred in 50 mL of water until dissolution of potassium salts, catalyst was filtered off, washed twice with water (10 mL), methanol (10 mL), dried in vacuum and stored for further use. Diethyl ether (100 mL) was added to the product containing filtrate and the solution was then washed with 100 mL of water (three times). Organic phase was dried using anhydrous sodium sulfate and solvents were removed under reduced pressure. Product(s) was/were separated by flash chromatography on silica gel using methanol-dichloromethane (1:9) mixture as a mobile phase. Yields of products are listed in Table 2.

### 3.3. Synthesis of 4-Chloro-3,5-dimethylpyridin-1-ium Chloride

The mixture of 3,5-lutidine (5.0 g, 46.7 mmol) and thionyl chloride (10 mL, 137 mmol) was refluxed for 24 h under argon atmosphere. After 24 h, the reaction mixture was cooled and 40 mL of toluene were added. Twenty-milliliter volumes of the mixture were distilled under vacuum. The brown precipitate was collected by filtration. The crude product was recrystallized from methanol [28]. Yield: 60%, m.p. 130 °C. Melting point not reported in literature. 

### 3.4. Synthesis of 4-Chloro-1-methylpyridin-1-ium Iodide 

4-Chloropyridin-1-ium chloride (3 g, 19.99 mmol) was neutralized by 0.5 M KOH in ice cold water. Subsequently 4-chloropyridine was extracted into cold dichlomethane, which was later removed under reduced pressure at 0 °C. Excess of methyl iodide was added into the resultant 4-chloropyridine and solution was stirred for 20 h at 0 °C in order to get 98% yield of 4-chloro-1-methylpyridinium iodide, m.p. 161 °C (dichloromethane), literature 156–159 °C (diethyl ether: methanol) [29].

### 3.5. General Synthetic Procedure for C-N Coupling of Selected 4-Chloropyridine Derivatives with 4-Methoxyaniline

A mixture of the corresponding 4-chloropyridine derivative (8.12 mmol), 4-methoxyaniline (1.19 g, 9.74 mmol), anhydrous potassium carbonate (3.45 g, 25 mmol) and supported Cu(I) catalyst (100 mg, 0.22 mmol of Cu, 2.7 mol % of Cu) was refluxed in isopropyl alcohol (40 mL) for 24 h under open atmosphere conditions. After 24 h, reaction mixture was filtered to remove potassium salts and catalyst. The solid was stirred in 50 mL of water until dissolution of potassium salts, catalyst was filtered off, washed twice with water (10 mL), methanol (10 mL), dried in vacuum and stored for further use. Diethyl ether (100 mL) was added to the product containing filtrate and the solution was then washed with 100 mL of water (three times). Organic phase was dried using anhydrous sodium sulfate and solvents were removed under reduced pressure. Product(s) was/were separated by flash chromatography on silica gel using methanol-dichloromethane (1:9) mixture as a mobile phase. Yields of products see Table 3.

### 3.6. Synthesis of 1-(Pyrid-4-yl)piperazine

4-Chloropyridin-1-ium chloride (1.5 g, 10 mmol) was dissolved in 100 ml of isopropyl alcohol at 60 °C. Piperazine (2.15 g, 25 mmol) and supported Cu(I) catalyst (100 mg, 0.22 mmol) was added into the resulted solution. The reaction mixture was refluxed for 13 h and monitored by TLC on silica gel plates, methanol was used as a mobile phase and spots were developed by ninhydrin. After the completion of a reaction, the reaction mixture was cooled to 5–10 °C, the precipitated piperazine-1,4-diium dichloride was filtered off together with the catalyst, the filtrate was filtered with the addition of active carbon, and the solvent distilled off in vacuum to dryness. The residue of crude 1-(pyrid-4-yl)piperazine was recrystallized from *n*-heptane with the addition of silica gel. The yield of the reaction was 11.5 g (70%) of 1-(pyridin-4-yl)piperazine and the purity was determined by gas chromatography and achieved 99.1%. The insoluble residue during crystallization of the crude product was crystallized in boiling *n*-heptane. The filtrate was then concentrated to 1/3 of the original volume and allowed to crystallize at 5–10 °C overnight. It gave an additional yield 2.5 g (15.3%) of 1-(pyrid-4-yl)piperazine and its purity of 97.8% was determined by gas chromatography as well. 

## 4. Conclusions

In conclusion, we found that recyclable copper(I) supported on weakly acidic polyacrylate resin catalyzes C-N cross coupling reaction in the absence of additional ligand. Described supported system catalyzes C-N cross coupling reactions of 4-chloropyridinium chloride with anilines containing electron donating (EDG) or electron withdrawing (EWG) groups, naphthalen-2-amine and piperazine, efficiently.

C-N cross coupling gave two types of products, i.e., *N*-(pyridin-4-yl)benzene amines and/or *N*,*N*-bis(pyridine-4-yl)benzene amines depends on electronic effect on aniline and type of base used.

A molar excess of piperazine as a base used in the case of coupling reaction realized between piperazine and 4-chloropyridinium chloride. Only formation of the 1-(pyridin-4-yl)piperazine in very good yield was observed as far as this reaction was realized in isopropyl alcohol. Synthesis in methanol or ethanol as solvents gave in addition to target 1-(pyridin-4-yl)piperazine product the corresponding 4-methoxy- or 4-ethoxypyridine.

C-N cross coupling reactions of 4-methoxyaniline with 4-chloro-1-methylpyridinium iodide shows better activity than that with 4-chloropyridinium chloride. There was no C-N cross coupling reaction observed with 4-chloro-3,5-dimethylpyridine and 4-methoxyaniline.

The applied novel supported catalytic Cu(I) system is very stable, insensitive to moisture and atmospheric oxygen. Hence, the described C-N coupling reactions can be carried out under open atmosphere and generally mild conditions. The applied Cu(I) catalyst is less toxic, economical, and easily preparable, separable, and recyclable (more than twenty times at full conversion of the starting 4-chloropyridinium chloride and the same reaction time). Therefore, the studied syntheses may be regarded as environmentally clean and green processes.

## Figures and Tables

**Table 1:** Comparison of different catalytic conditions for C-N coupling of 4-methoxyaniline with 4-chloropyridine.

Sr. No.	Catalyst	Product Yield (%)	TON
1	Cu(I) ions supported on a weekly acidic cation-exchanger resin	64	23.6
2	Copper(I) iodide	8	2.9
3	No catalyst	0	0

*Reaction conditions*: A mixture of 4-methoxyaniline (1.20 g, 9.74 mmol), 4-chloropyridin-1-ium chloride (1.21 g, 8.12 mmol), anhydrous potassium carbonate (3.45 g, 25 mmol) and supported Cu(I) catalyst (100 mg, 0.22 mmol) or copper(I) iodide (50.0 mg, 0.26 mmol) or no catalyst was refluxed in isopropyl alcohol (40 mL) for 24 h in open atmosphere conditions.

**Table 2:** Comparison of pure product yields for different aromatic amines in C-N coupling reaction with 4-chloropyridin-1-ium chloride in the presence of potassium carbonate as a base.

Entry	Amine	Product	Yield (%)	TON
1	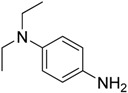	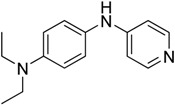	80	29.5
2	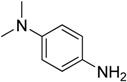	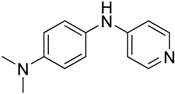	78	28.7
3	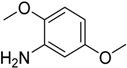	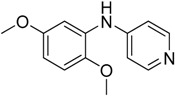	22	8.1
4	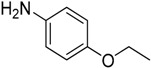	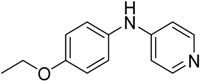	68	25
5	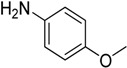	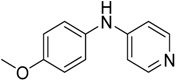	64	23.6
6	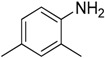	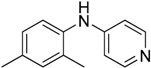	12	4.4
7	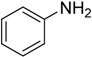	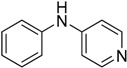	40	14.7
8	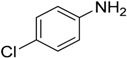	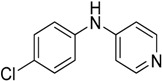	46	16.9
9	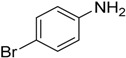	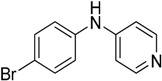	47	17.3
10	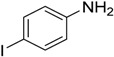	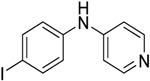	45	16.6
11	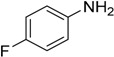	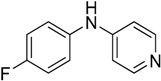	49	18
12	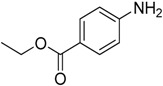	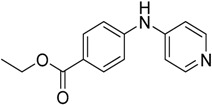	26	9.5
13	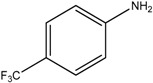	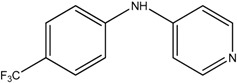	24	30.9
		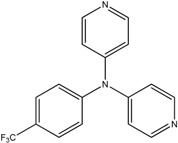	30	
14	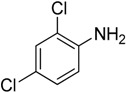	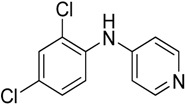	10	27.3
		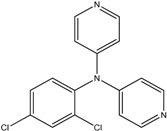	32	
15	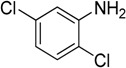	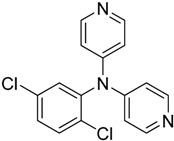	42	31
16	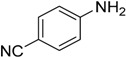	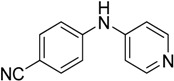	9	32.10
		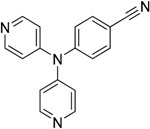	39	
17	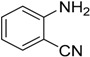	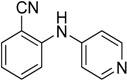	6	20
		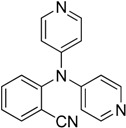	24	
18	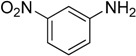	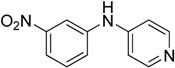	6	31
		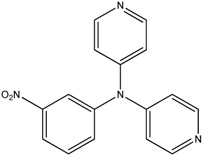	39	
19	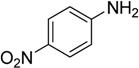	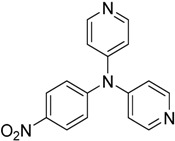	42	32.4
		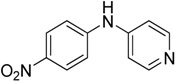	4	
20	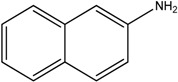	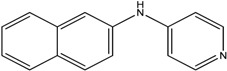	65	24

*Reaction conditions*: A mixture of aryl amine (9.74 mmol), 4-chloropyridin-1-ium chloride (1.21 g, 8.12 mmol), anhydrous potassium carbonate (3.45 g, 25 mmol) and supported Cu(I) catalyst (100 mg, 0.22 mmol) was refluxed in isopropyl alcohol (40 mL) for 24 h in open atmosphere conditions.

**Table 3:** Comparison of yields for selected 4-chloropyridine derivatives in C-N coupling reaction with 4-methoxyaniline.

Entry	4-Chloropyridine Derivative	Product	Yield (mol %)	TON
1	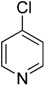	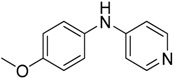	64	23.6
2	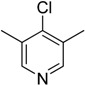	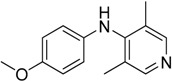	0	0
3	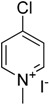	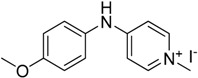	80	29.5

*Reaction conditions*: A mixture of the corresponding 4-chloropyridine derivative (8.12 mmol), 4-methoxyaniline (9.74 mmol), anhydrous potassium carbonate (3.45 g, 25 mmol) and 100 mg (0.22 mmol) of Cu(I) ions supported on weakly acidic cation-exchanger resin was refluxed in isopropyl alcohol (40 mL) for 24 h in conditions of an open atmosphere.

## Supplementary Materials

Supplementary materials can be accessed at: http://www.mdpi.com/1420-3049/22/1/2/s1.
