# Supplementary material for: New Stable Cu(I) Catalyst Supported on Weakly Acidic Polyacrylate Resin for Green C-N Coupling: Synthesis of N-(Pyridin-4-yl)benzene Amines and N,N-Bis(pyridine-4-yl)benzene Amines
Source: Molecules. 2016 Dec 22;22(1):2. doi: 10.3390/molecules22010002 (PMC6155682; doi:10.3390/molecules22010002)
Supplement: Supplementary file 1 [file molecules-22-00002-s001.pdf]

Supplementary Materials: New Stable Cu(I) Catalyst Supported on Weakly Acidic Polyacrylate Resin for Green C-N Coupling: Synthesis of *N*-(Pyridin-4-yl)benzene Amines and *N*,*N*-Bis(pyridine-4-yl)benzene Amines

Nitin Kore and Pavel Pazdera

1. Synthesis of 4-Chloro-3,5-Dimethylpyridin-1-ium Chloride

The mixture of 3,5-lutidine (5 g, 46.7 mmol) and thionyl chloride (10 mL, 137 mmol) was refluxed for 24 h under argon atmosphere. After 24 h, the reaction mixture was cooled and 40 mL of toluene were added. 20 mL volumes of the mixture were distilled under vacuum. The heavy brown precipitate was collected by filtration [1]. The crude product was recrystallized from methanol [1]. Yield: 60 %, m.p. 130 °C. Melting point not reported in literature.

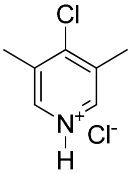

^{1}H-NMR (300 MHz, DMSO) δ 8.65 (s, 2H, H_{Py}-2, H_{Py}-6), 8.31 (s, 1H, NH), 2.46 (s, 6H, CH_3). ^{13}C-NMR (126 MHz, D_2O) δ 148.2 (1C, C_{Py}-4), 138.3 (2C, C_{Py}-2, C_{Py}-6), 137.7 (2C, C_{Py}-3, C_{Py}-5), 17.5 (2C, CH_3).

2. Synthesis of 4-Chloro-1-methylpyridin-1-ium Iodide

4-Chloropyridin-1-ium chloride (3 g, 19.99 mmol) was neutralized by 0.5 M KOH in ice bath. Subsequently 4-chloropyridine was extracted into cold dichlomethane, which was next removed under reduced pressure at 0 °C. Excess of methyl iodide was added into the resultant 4-chloro pyridine and solution was stirred for 20 h at 0 °C in order to get 98% yield of 4-chloro-1–methylpyridinium iodide [2], m.p. 161 °C (dichloromethane), literature 156–159 °C (diethyl ether:methanol) [2].

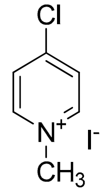

^{1}H-NMR (300 MHz, D_2O) δ 8.73 (d, 2H, H_{Py}-2, H_{Py}-6), 8.10 (d, *J* = 9.0 Hz, 2H, H_{Py}-3, H_{Py}-5), 4.34 (s, 3H, CH_3). ^{13}C-NMR (126 MHz, D_2O) δ 153.9 (1C, C_{Py}-4), 146.1 (2C, C_{Py}-2, C_{Py}-6), 128.6 (2C, C_{Py}-3, C_{Py}-5), 48.1 (1C, CH_3).

3. Synthesis of 1-(Pyridin-4-yl)piperazine

4-Chloropyridin-1-ium chloride (1.5 g, 10 mmol) was dissolved in 100 ml of isopropyl alcohol at 60 °C. Piperazine (2.15 g, 25 mmol) and 100 mg (0.22 mmol) of Cu(I) catalyst was added into the resulted solution. The reaction mixture was refluxed for 13 h and reaction was monitored by TLC on silica gel plates, methanol was used as a mobile phase and spots were developed by ninhydrin. After the completion of a reaction, the reaction mixture was cooled to 5–10 °C, the precipitated piperazine-1,4-diium dichloride was filtered off together with the catalyst, the filtrate was filtered with the addition of active carbon, and the solvent distilled off in vacuum. The residue of crude 1-(pyrid-4-yl) -piperazine was recrystallized from n-heptane with the addition of a silica gel. The yield of 1-(pyridin-

4-yl)piperazine was 11.5 g (70%) and the purity determined by gas chromatography achieved 99.1 %. The insoluble residue from the crystallization of the crude product was crystallized from *n*-heptane and the filtrate was then concentrated to 1/3 of the original volume and allowed to crystallize at 5–10 °C overnight. It gave an additional yield 2.5 g (15.3%) of 1-(pyrid-4-yl)piperazine and its purity of 97.8% was determined by gas chromatography as well.

4. General Synthetic Procedure for C-N Coupling of Selected 4-Chloropyridine Derivatives with 4-Methoxyaniline

A mixture of the corresponding 4-chloropyridine derivative (1.21 g, 8.12 mmol), 4-methoxyaniline (1.20 g, 9.74 mmol), anhydrous potassium carbonate (3.45 g, 25 mmol) and supported Cu(I) catalyst (100 mg, 0.22 mmol) was refluxed in isopropyl alcohol (40 mL) for 24 h under open atmosphere conditions. After 24 h, reaction mixture was filtered to remove potassium salts and catalyst. The solid was stirred in 50 mL of water until dissolution of potassium salts, catalyst was filtered off, washed twice with water (10 mL), methanol (10 mL), dried in vacuum and stored for further use. Diethyl ether (100 mL) was added to the product containing filtrate and washed with 100 mL of water (three times). Organic phase was dried using anhydrous sodium sulfate and solvents were removed under reduced pressure. Product(s) was/were separated by flash chromatography on silica gel using methanol–dichloromethane (1:9) mixture as a mobile phase.

5. Study of Recycling of Catalyst for C-N Coupling of 4-Methoxyaniline with 4-Chloropyridin-1-ium Chloride

A mixture of 4-methoxyaniline (1.20 g, 9.74 mmol), 4-chloropyridin-1-ium chloride (8.12 mmol), anhydrous potassium carbonate (25 mmol) and 100 mg (0.22 mmol, 0.22 mmol) of Cu(I) ions supported on weakly acidic cation-exchanger resin was refluxed in isopropyl alcohol under open atmosphere condition. Reaction is monitored by TLC. After 24 h, there was no 4-chloropyridine observed on TLC. The reaction mixture was filtered to remove catalyst. Diethyl ether (100 mL) was added to the filtrate and the solution was then washed with 100 mL of water (three times). Organic phase was dried using sodium sulfate and solvents were removed under reduced pressure. The brown solid purified by recrystallization in acetonitrile gave *N*-(4-methoxyphenyl)pyridin-4-amine (**11**) as white crystals (1.04 g, 5.2 mmol, 64%). Because of large size of resin, all catalyst recovered very easily by simple filtration. Catalyst recovered from reaction mixture washed with methanol and dried at room temperature and used it for next run. The same reaction procedure repeated using recycled catalyst.

Table S1. Study of recycling catalyst.

Run No.	Yield (%)	Run No.	Yield (%)	Run No.	Yield (%)	Run No.	Yield (mol %)
1	64	6	62	11	64	16	61
2	64	7	63	12	63	17	62
3	63	8	64	13	62	18	63
4	64	9	62	14	61	19	61
5	61	10	61	15	64	20	63

6. Typical Physical & Chemical Characteristics of Purolite® C104 Plus

Table S2. Typical physical & chemical characteristics of Purolite® C104 Plus.

Polymer Structure	Porous crosslinked polyacrylic acid
Appearance	Spherical beads
Functional Group	Carboxylic Acid
Ionic Form	H^+
Total Capacity	4.5 eq/L (98.3 Kgr/ft^3) (H^+ form)
Moisture Retention	45–55 % (H^+ form)
Particle Size Range	300–1600 μm
<300 μm (max.)	1 %
Reversible Swelling, $H^+ \rightarrow Ca^{2+}$ (max.)	20 %
Reversible Swelling, $H^+ \rightarrow Ca^{2+}$ (operating)	7 * %
Reversible Swelling, $H^+ \rightarrow Na^+$ (max.)	70 %
Specific Gravity	1.19
Shipping Weight (approx.)	740–780 g/L (46.2–48.8 lb/ft^3)
Temperature Limit	120 °C (248.0 °F)
Footnote	(approximately)

7. General Synthetic Procedure for C-N Coupling of Arylamine with 4-Chloropyridin-1-ium Chloride

A mixture of aryl amine (9.74 mmol), 4-chloropyridin-1-ium chloride (8.12 mmol), anhydrous potassium carbonate (25 mmol) and 100 mg (0.22 mmol) of Cu(I) ions supported on weakly acidic cation-exchanger resin was refluxed in isopropyl alcohol for 24 h under open atmosphere conditions. After 24 h, reaction mixture was filtered to remove K_2CO_3 and catalyst. Diethyl ether (100 mL) was added to the filtrate and the solution was then washed with 100 mL of water (three times). Organic phase was dried using sodium sulfate and solvents were removed under reduced pressure. Products were purified either by recrystallization or by flash chromatography using methanol–dichloromethane (1:9) as a mobile phase.

Table S3. Comparison of pure product yields for different aromatic amines in C-N coupling reaction with 4-chloropyridin-1-ium chloride

Entry	Amine	Product	Yield (mol %)	TON
1	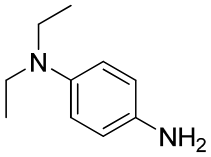	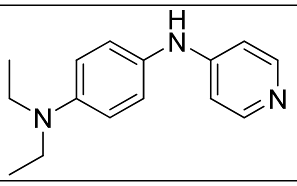	80	29.5
2	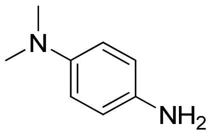	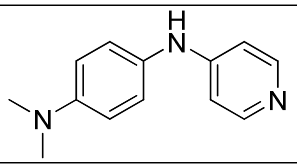	78	28.7
3	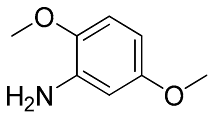	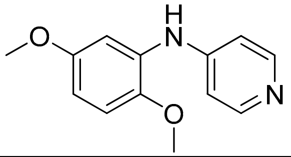	22	8.1
4	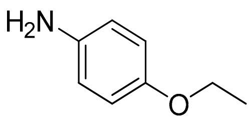	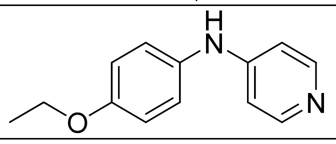	68	25

5	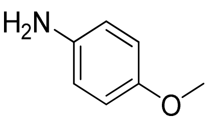	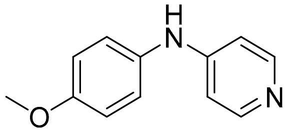	64	23.6
6	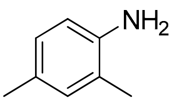	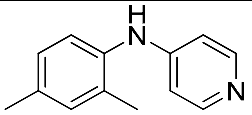	12	4.4
7	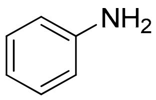	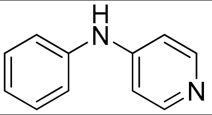	40	14.7
8	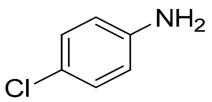	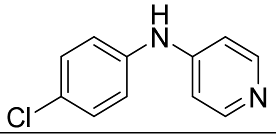	46	16.9
9	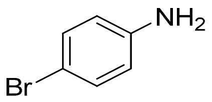	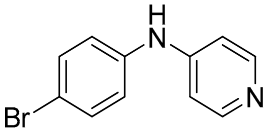	47	17.3
10	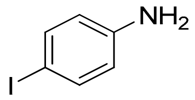	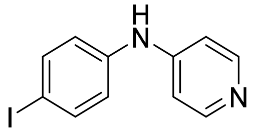	45	16.6
11	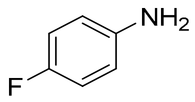	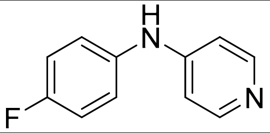	49	18
12	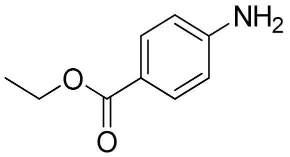	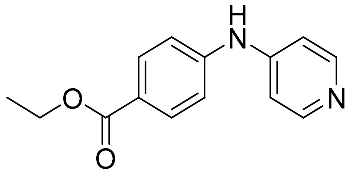	26	9.5
13	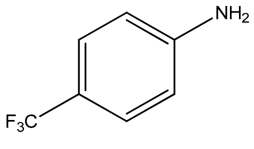	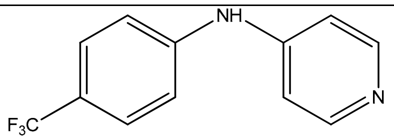<br>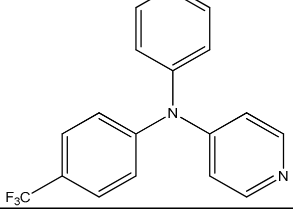	24<br>30	30.9
14	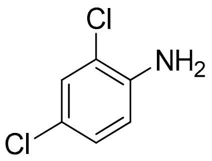	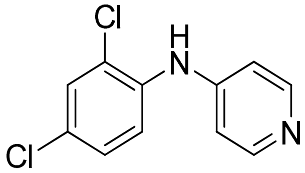<br>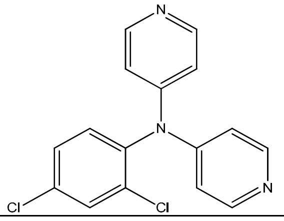	10<br>32	27.3

15	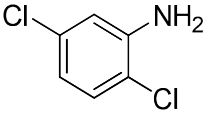	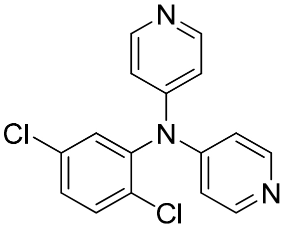	42	31
16	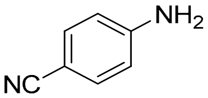	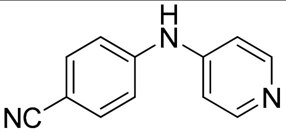	9	32.10
		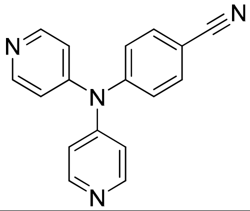	39	
17	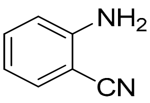	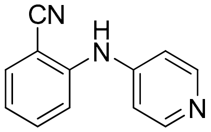	6	20
		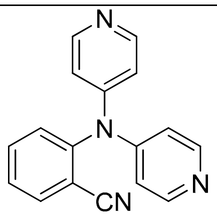	24	
18	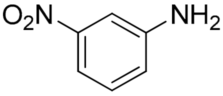	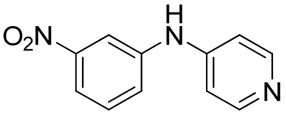	6	31
		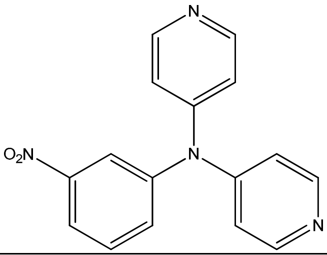	39	
19	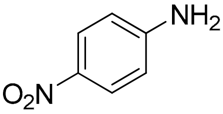	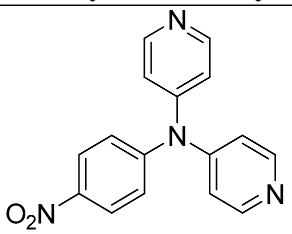	42	32.4
		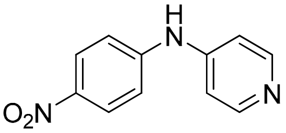	4	
20	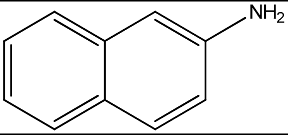	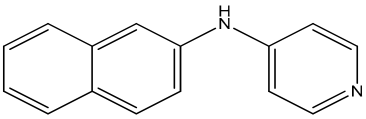	65	24

Product characterization

*1) N*1,*N*1-Diethyl-*N*4-(pyridin-4-yl)benzene-1,4-diamine (Table 1, Entry 1)

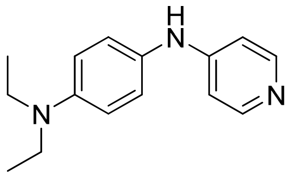

State: brown sticky material; Molecular formula: $C_{15}H_{19}N_3$. HRMS (APCI) *m/z* calculated/found: $[M + H]^+$ = 242.1652/242.1651. ^{1}H-NMR (300 MHz, $CDCl_3$) δ/ppm 8.12–7.07 (m, 4H, H_{Py}), 6.76 (s, 1H, NH), 6.74–6.64 (m, 4H, H_{Ar}), 3.37 (q, *J* = 7.1 Hz, H_{CH2}), 1.19 (t, *J* = 7.1 Hz, H_{CH3}). ^{13}C-NMR (126 MHz, $CDCl_3$) δ 153.7 (2C, C_{Py}-2, C_{Py}-6), 148.0 (1C, C_{Py}-4), 146.1 (1C, $C_{Ar\text{-}NEt2}$), 126.7 (1C, $C_{Ar\text{-}NHPy}$), 126.0 (2C, C_{Ar}), 112.5 (2C, C_{Py}-3, C_{Py}-5), 108.4 (2C, C_{Ar}), 44.5 (2C, CH_2), 12.5 (2C, CH_3). IR(KBr), ν (cm^{-1}) 3222 (ν, NH), 3136 (ν, NH), 3037 (ν, Ar-H), 2964, 2927, 2887, 2870 (ν, C-H), 1643(ν, Py-C=N), 1598 (ν, C=C) 1513, 1393, 1372, 1342, 1326, 1261, 1211, 1197, 1150, 1075, 1013, 989, 887, 807, 784, 737, 607.

2) *N*1,*N*1-Dimethyl-*N*4-(pyridin-4-yl)benzene-1,4-diamine (Table 1, Entry 2)

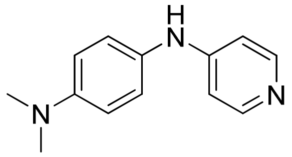

State: brown solid; Molecular formula: $C_{13}H_{15}N_3$. Melting point: 196 ° C (acetonitrile). HRMS (APCI) *m/z* calculated/found: $[M + H]^+$ = 214.1339/214.1339. ^{1}H-NMR (300 MHz, DMSO) δ 8.32 (s, 1H, NH), 8.07–7.04 (m, 4H, H_{Py}), 6.66–6.76 (m, 4H, H_{Ar}), 2.88 (s, 6H, H_{CH3}). ^{13}C-NMR (126 MHz, DMSO) δ 152.4 (2C, C_{Py}-2, C_{Py}-6), 150.2 (1C, C_{Py}-4), 148.0 (1C, $C_{Ar\text{-}Me2}$), 129.7 (1C, $C_{Ar\text{-}NHPy}$), 124.2 (2C, C_{Ar}), 113.8 (2C, C_{Py}-3, C_{Py}-5), 108.5 (2C, C_{Ar}), 41.0 (2C, CH3). IR(KBr), ν (cm^{-1}) 3216 (ν, NH), 3135 (ν, NH), 3099 (ν, Ar-H), 3033 (ν, Ar-H), 2959, 2883 (ν, C-H), 2798, 1612, 1587 (ν, Ar-C=C), 1511, 1475, 1442, 1432, 1339, 1321, 1209, 1093, 1052, 989, 944, 887, 854, 801, 720, 643, 607, 528, 512.

3) *N*-(2,5-Dimethoxyphenyl)pyridin-4-amine (Table 1, Entry 3)

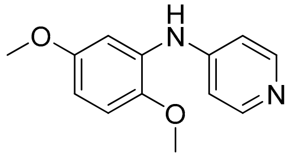

State: brown viscus liquid; Molecular formula: $C_{13}H_{14}N_2O_2$. HRMS (APCI) *m/z* calculated/found: $[M + H]^+$ = 231.1128/231.1130. ^{1}H-NMR (300 MHz, $CDCl_3$) δ 8.28 (d, *J* = 4.7 Hz, 2H, H_{Py}-2, H_{Py}-6), 6.98 (s, 1H, NH), 6.90 (d, *J* = 5.2 Hz, 2H, H_{Py}-3, H_{Py}-5), 6.25–6.82 (m, 3H, H_{Ar}), 3.80 (s, 3H, OCH_3), 3.75 (s, 3H, H_{OCH3}). ^{13}C-NMR (75 MHz, $CDCl_3$) δ 153.8 (1C, $C_{Ar\text{-}OCH3}$), 150.3 (2C, C_{Py}-2, C_{Py}-6), 149.8 (1C, C_{Py}-4), 144.5 (1C, $C_{Ar\text{-}OCH3}$), 130.1 (1C, $C_{Ar\text{-}NHPy}$), 111.8 (2C, C_{Py}-3, C_{Py}-5), 110.3 (1C, C_{Ar}), 106.8 (1C, C_{Ar}), 06.3 (1C, C_{Ar}), 56.2 (OCH_3), 55.7 (2C, OCH_3). IR(KBr), ν (cm^{-1}) 3240 (ν, NH), 3137(ν, NH), 3032 (ν, Ar-H), 2996 (ν, Ar-H), 2933, 2832 (ν, Ar-H), 1585 (ν, Ar-C=C), 1519, 1500, 1461, 1433, 1411, 1340, 1298, 1280, 1212, 1179, 1161, 1130, 1041, 1022, 989, 863, 813, 735, 709, 644, 616, 588, 520.

4) *N*-(4-Ethoxyphenyl)pyridin-4-amine (Table 1, Entry 4)

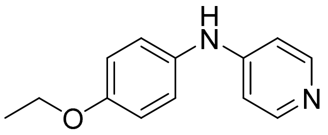

State: white solid; Molecular formula: $C_{13}H_{14}N_2O$. Melting point: 149 °C (acetonitrile). HRMS (APCI) *m*/*z* calculated/found: $[M + H]^+$ = 215.1179/215.1185. ^{1}H-NMR (300 MHz, Acetone-D6) δ 8.13 (s, 2H, 2H, H_{Py}-2, H_{Py}-6), 7.73 (s, 1H, NH), 7.17 (d, *J* = 8.9 Hz, 2H, 2H, H_{Py}-3, H_{Py}-5), 6.76–6.93 (m, 4H, H_{Ar}), 4.04 (q, *J* = 7.0 Hz, 2H, H_{CH2}), 1.36 (t, *J* = 7.0 Hz, 3H, H_{CH3}). ^{13}C-NMR (75 MHz, DMSO) δ 155.3 (1C, $C_{Ar\text{-}OEt}$), 151.8 (2C, C_{Py}-2, C_{Py}-6), 150.3 (1C, C_{Py}-4), 133.4 (1C, $C_{Ar\text{-}NHPy}$), 123.7 (2C, C_{Ar}), 115.6 (2C, C_{Ar}), 108.9 (2C, C_{Py}-3, C_{Py}-5), 63.7 (1C, CH_2), 15.1 (1C, CH_3). IR(KBr), ν (cm^{-1}) 3251 (ν, NH), 3156 (ν, NH), 2970, 2838 (ν, C-H), 1592 (ν, Ar-C=C), 1497, 1474, 1432, 1389, 1347, 1327, 1301, 1286, 1236, 1213, 1169, 1113, 1047, 993, 922, 891, 843, 806, 741, 660, 645, 601, 567.

5) *N*-(4-Methoxyphenyl)pyridin-4-amine (Table 1, Entry 5)

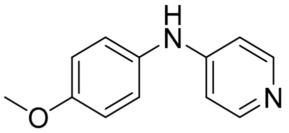

State: white solid; Molecular formula: $C_{12}H_{12}N_2O$. Melting point: 170 °C (acetonitrile) Lit 167 °C (aq. methanol) [3]. HRMS (APCI) *m*/*z* calculated/found: $[M + H]^+$ = 201.1022/201.1019. ^{1}H-NMR (300 MHz, $CDCl_3$) δ 8.23–7.13 (m, 4H, H_{Py}), 6.64 -6.91(m, 4H, H_{Ar}), 5.96 (s, 1H, NH), 3.82 (s, 3H, H_{OCH3}) [4]. ^{13}C-NMR (75 MHz, $CDCl_3$) δ 157.1 (1C, $C_{Ar\text{-}OCH3}$), 151.9 (2C, C_{Py}-2, C_{Py}-6), 150.3 (1C, C_{Py}-4), 132.1 (1C, $C_{Ar\text{-}NHPy}$), 125.2 (2C, C_{Ar}), 114.8 (2C, C_{Ar}), 108.7 (2C, C_{Py}-3, C_{Py}-5), 55.5 (1C, OCH_3). IR(KBr), ν (cm^{-1}) 3152 (ν, NH), 2833 (ν, C-H), 1611, 1591 (ν, Ar-C=C), 1505, 1343, 1326, 1289, 1234, 1213, 1182, 1170, 1112, 1095, 1037, 992, 891, 845, 812, 765, 737, 660, 647, 604, 544, 527, 526.

6) *N*-(2,4-Dimethylphenyl)pyridin-4-amine (Table 1, Entry 6)

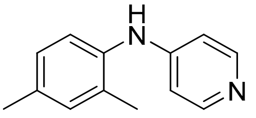

State: white solid; Molecular formula: $C_{13}H_{14}N_2$. Melting point: 162 °C (acetonitrile). HRMS (APCI) *m*/*z* calculated/found: $[M + H]^+$ = 199.1230/199.1232. ^{1}H-NMR (500 MHz, $CDCl_3$) δ 8.23 (s, 2H, H_{Py}-2, H_{Py}-6), 7.03–7.17 (m, 3H, H_{Ar}), 6.55 (s, 2H, H_{Py}-3, H_{Py}-5), 5.77 (s, 1H, NH), 2.33 (s, 3H, H_{CH3}), 2.19 (s, 3H, H_{CH3}). ^{13}C-NMR (126 MHz, $CDCl_3$) δ 152.0 (2C, C_{Py}-2, C_{Py}-6), 150.2 (1C, C_{Py}-4), 135.8 (1C, $C_{Ar\text{-}NHPy}$), 134.8 (1C, $C_{Ar\text{-}CH3}$), 133.4 (1C, C_{Ar}), 131.9 (1C, $C_{Ar\text{-}CH3}$), 127.6 (1C, C_{Ar}), 125.3 (1C, C_{Ar}), 108.8 (2C, C_{Py}-3, C_{Py}-5), 20.9 (1C, CH_3), 17.8 (1C, CH_3). IR(KBr), ν (cm^{-1}) 3237 (ν, NH), 3155 (ν, NH), 2959, 2911 (ν, C-H), 1592 (ν, Ar-C=C), 1513, 1482, 1426, 1377, 1340, 1320, 1261, 1230, 1212, 1120, 1096, 1033, 991, 875, 847, 832, 808, 736, 717, 688, 657, 630, 560.

7) *N*-Phenylpyridin-4-amine (Table 1, Entry 7)

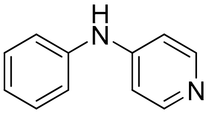

State: white solid; Molecular formula: $C_{11}H_{10}N_2$. Melting point: 176 °C (acetonitrile) Lit 173.5–175 °C (aq. ethanol) [5]. HRMS (APCI) *m*/*z* calculated/found: $[M + H]^+$ = 171.0917/171.0912. ^{1}H-NMR (300 MHz,

$CDCl_3$) δ 8.28–7.36 (m, 4H, H_{Py}), 7.19–6.82 (m, 4H, H_{Ar}), 6.56 (s, 1H, NH) [4]. ^{13}C-NMR (75 MHz, $CDCl_3$) δ 150.7 (2C, C_{Py}-2, C_{Py}-6), 150.5 (1C, C_{Py}-4), 139.7 (1C, $C_{Ar\text{-}NHPy}$), 129.5 (2C, C_{Ar}), 124.0 (2C, C_{Ar}), 121.6 (1C, C_{Ar}), 109.5 (2C, C_{Py}-3, C_{Py}-5). IR(KBr), ν (cm^{-1}) 3251 (ν, NH), 3155 (ν, NH), 2832 (ν, C-H), 1610, 1584 (ν, Ar-C=C), 1523, 1490, 1446, 1426, 1348, 1333, 1234, 1216, 1174, 1156, 1076, 1026, 993, 892, 832, 806, 748, 693, 662, 636, 613, 568, 523.

8) *N*-(4-Chlorophenyl)pyridin-4-amine (Table 1, Entry 8)

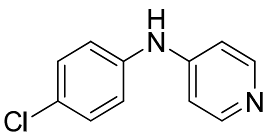

State: white solid; Molecular formula: $C_{11}H_9ClN_2$. Melting point: 249 °C (acetonitrile) Lit 250–252 °C (acetonitrile) [6]. HRMS (APCI) *m*/*z* calculated/found: $[M + H]^+$ = 205.0527/205.0525 [7]. ^{1}H-NMR (300 MHz, $CDCl_3$) δ 8.31–7.33 (m, 4H, H_{Py}), 6.78–7.13 (m, 4H, H_{Ar}), 5.96 (s, 1H, NH). ^{13}C-NMR (75 MHz, DMSO) δ 150.6 (2Py), 150.1 (1C, C_{Py}-4), 140.0 (1C, $C_{Ar\text{-}NHPy}$), 129.6 (2C, C_{Ar}), 126.3 (1C, $C_{Ar\text{-}Cl}$), 121.8 (2C, C_{Ar}), 109.9 (2C, C_{Py}-3, C_{Py}-5). IR(KBr), ν (cm^{-1}) 3273 (ν, NH), 3168 (ν, NH), 2891 (ν, C-H), 2786, 1892, 1635, 1607, 1581 (ν, Ar-C=C), 1523, 1483, 1428, 1341, 1234, 1214, 1171, 1110, 1083, 1058, 995, 834, 805, 708, 655, 635, 601, 525.

9) *N*-(4-Bromophenyl)pyridin-4-amine (Table 1, Entry 9)

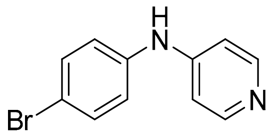

State: Yellowish white solid; Molecular formula: $C_{11}H_9BrN_2$. Melting point: 264 °C (acetonitrile) Lit. 264–265 °C (acetonitrile) [8]. HRMS (APCI) *m*/*z* calculated/found: $[M + H]^+$ = 249.0022/249.0024. ^{1}H-NMR (500 MHz, DMSO) δ 8.88 (s, 1H, NH), 8.22–7.49 (m, 4H, H_{Py}), 6.91–7.16 (m, 4H, H_{Ar}). ^{13}C-NMR (126 MHz, DMSO) δ 150.7 (2C, C_{Py}-2, C_{Py}-6), 150.0 (1C, C_{Py}-4), 140.5 (1C, $C_{Ar\text{-}NHPy}$), 132.5 (2C, C_{Ar}), 122.0 (2C, C_{Ar}), 114.0 (1C, $C_{Ar\text{-}Br}$), 110.0 (2C, C_{Py}-3, C_{Py}-5). IR(KBr), ν (cm^{-1}) 3270 (ν, NH), 3164 (ν, NH), 2788, 1891, 1633, 1603 (ν, Ar-C=C), 1523, 1483, 1428, 1346, 1233, 1215, 1172, 1109, 1069, 995, 849, 803, 703, 649, 595, 525.

10) *N*-(4-Iodophenyl)pyridin-4-amine (Table 1, Entry 10)

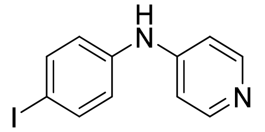

State: Yellowish white solid; Molecular formula: $C_{11}H_9IN_2$. Melting point: 244 °C (acetonitrile). HRMS (APCI) *m*/*z* calculated/found: $[M + H]^+$ = 296.9883/296.9882. ^{1}H-NMR (500 MHz, DMSO) δ 8.86 (s, 1H, NH), 8.22–7.64 (m, 4H, H_{Py}), 6.91–7.03 (m, 4H, H_{Ar}). ^{13}C-NMR (126 MHz, DMSO) δ 150.7 (2C, C_{Py}-2, C_{Py}-6), 149.8 (1C, C_{Py}-4), 141.0 (1C, $C_{Ar\text{-}NHPy}$), 138.3 (2C, C_{Ar}), 122.3 (2C, C_{Ar}), 110.1 (2C, C_{Py}-3, C_{Py}-5), 85.4 (1C, $C_{Ar\text{-}I}$). IR(KBr), ν (cm^{-1}) 3263 (ν, NH), 3158 (ν, NH), 2804, 1720, 1628, 1604, (ν, Ar-C=C), 1519, 1476, 1428, 1342, 1231, 1214, 1176, 1058, 994, 849, 803, 763, 700, 646, 592, 525.

11) *N*-(4-Fluorophenyl)pyridin-4-amine (Table 1, Entry 11)

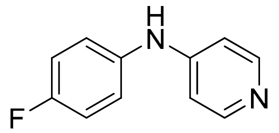

State: Pale brown solid; Molecular formula: $C_{11}H_9FN_2$. Melting point: 201 °C (acetonitrile) Lit. 200–201 °C (acetonitrile) [8]. HRMS (APCI) *m*/*z* calculated/found: $[M + H]^+$ = 189.0823/189.0821. ^{1}H-NMR (500 MHz, DMSO) δ 8.71 (s, 1H, NH), 8.18 (s, 2H, H_{Py}-2, H_{Py}-6), 7.33–7.09 (m, 4H, H_{Ar}), 6.82 (d, *J* = 5.9 Hz, 2H, H_{Py}-3, H_{Py}-5) [4]. ^{13}C-NMR (75 MHz, DMSO) δ 160.0 (1C, $C_{Ar\text{-}F}$), 156.8 (2C, C_{Py}-2, C_{Py}-6), 151.0 (2Py), 150.5 (1C, C_{Py}-4), 137.2 (1C, C_{Ar}), 137.1 (1C, C_{Ar}), 123.1 (1C, C_{Ar}), 123.0 (1C, C_{Ar}), 116.6 (2C, C_{Py}-3, C_{Py}-5), 116.3 (3Py), 109.3 (1C, $C_{Ar\text{-}NHPy}$). IR(KBr), ν (cm^{-1}) 3266 (ν, NH), 3167 (ν, NH), 3036 (ν, Ar-H), 2806, 1590 (ν, Ar-C=C), 1528, 1494, 1433, 1347, 1208, 1152, 1104, 1090, 1058, 995, 893, 838, 814, 772, 660, 646, 607, 521.

12) Ethyl 4-[(pyridin-4-ylamino)]benzoate (Table 1, Entry 12)

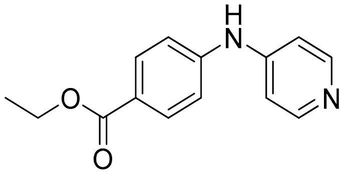

State: Yellow solid; Molecular formula: $C_{14}H_{14}N_2O_2$. Melting point: 191 °C (acetonitrile). HRMS (APCI) *m*/*z* calculated/found: $[M + H]^+$ = 243.1128/243.1132. ^{1}H-NMR (300 MHz, DMSO) δ 9.25 (s, 1H, NH), 8.30–7.91 (m, 4H, H_{Py}), 7.06–7.28 (m, 4H, H_{Ar}), 4.28 (q, *J* = 7.1 Hz, 2H, H_{CH2}), 1.31 (t, *J* = 7.1 Hz, 3H, H_{CH3}). ^{13}C-NMR (75 MHz, DMSO) δ 165.8 (C=O), 150.8 (2C, C_{Py}-2, C_{Py}-6), 149.0 (1C, C_{Py}-4), 146.0 (1C, $C_{Ar\text{-}NHPy}$), 131.3 (2C, C_{Ar}), 122.8 (1C, $C_{Ar\text{-}COOEt}$), 117.8 (2C, C_{Ar}), 111.3 (2C, C_{Py}-3, C_{Py}-5), 60.7 (1C, CH_2), 14.7 (1C, CH_3). IR(KBr), ν (cm^{-1}) 3267 (ν, NH), 3163 (ν, NH), 2983, 2898 (ν, C-H), 1701 (ν, C=O), 1630, 1584 (ν, Ar-C=C), 1519, 1501, 1474, 1430, 1411, 1346, 1306, 1267, 1213, 1172, 1103, 1026, 994, 895, 849, 827, 761, 697, 674, 653, 600, 524 [9].

13-A) *N*-(4-(Trifluoromethyl)phenyl)pyridin-4-amine (Table 1, Entry 13)

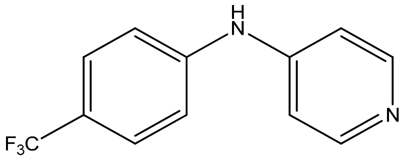

State: White solid; Molecular formula: $C_{12}H_9F_3N_2$. Melting point: 184 °C (dichlomethane:methanol, 9:1)

HRMS (APCI) *m*/*z* calculated/found: $[M + H]^+$ = 239.0791/239.0791. ^{1}H-NMR (500 MHz, DMSO) δ 9.21 (s, 1H, NH), 8.30 (d, *J* = 5.9 Hz, 2H, H_{Py}-2, H_{Py}-6), 7.05–7.65 (m, 4H, H_{Ar}), 7.05 (d, *J* = 6.3 Hz, 2H, H_{Py}-3, H_{Py}-5). ^{13}C-NMR (75 MHz, DMSO) δ 150.8 (1C, C_{Py}-4), 149.1 (2C, C_{Py}-2, C_{Py}-6), 145.1 (1C, $C_{Ar\text{-}NHPy}$), 127.0 (2C, C_{Ar}), 122.1 (CF3), 121.7 (1C, $C_{Ar\text{-}CF3}$), 118.6 (2C, C_{Ar}), 111.0 (2C, C_{Py}-3, C_{Py}-5). IR(KBr), ν (cm^{-1}) 3262 (ν, NH), 3163 (ν, NH), 3059 (ν, Ar-H), 2952, 2891, 2805, 1620, 1588 (ν, Ar-C), 1525, 1501, 1317, 1217, 1155, 1104, 1066, 997, 813, 758, 731, 672, 652, 602.

13-B) *N*-(Pyridin-4-yl)-*N*-[4-(trifluoromethyl)phenyl]pyridin-4-amine (Table 1, Entry 13)

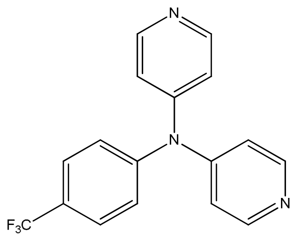

State: Yellow solid; Molecular formula: $C_{17}H_{12}F_3N_3$. Melting point: 96 °C (dichlomethane:methanol, 9:1) HRMS (APCI) *m*/*z* calculated/found: $[M + H]^+$ = 316.1056/316.1057. ^{1}H-NMR (500 MHz, $CDCl_3$) δ 8.72 (d, *J* = 6.2 Hz, 1H), 7.58 (d, *J* = 8.4 Hz, 1H), 7.40 (dd, *J* = 8.0, 2.3 Hz, 1H), 7.26 (dd, *J* = 8.1, 2.3 Hz, 1H), 7.22 (d, *J* = 6.3 Hz, 1H), 7.00 (d, *J* = 8.3 Hz, 1H), 6.53 (dd, *J* = 8.0, 2.6 Hz, 1H), 6.26 (dd, *J* = 8.1, 2.6 Hz, 1H). ^{13}C-NMR (75 MHz, $CDCl_3$) δ 155.6 (1C, C_{Py}-4), 155.0 (1C, C_{Py}-4), 151.9 (1C, $C_{Ar\text{-}N(Py)2}$), 148.7 (CF3), 134.4 (2C, C_{Py}-2, C_{Py}-6), 133.2 (2C, C_{Py}-2, C_{Py}-6), 126.5 (2C, C_{Ar}), 121.5 (2C, C_{Ar}), 118.0 (2C, C_{Py}-3, C_{Py}-5), 114.5 (2C, C_{Py}-3, C_{Py}-5), 108.7 (1C, $C_{Ar\text{-}CF3}$). IR(KBr), ν (cm^{-1}) 3044 (ν, Ar-H), 2922, 2851, 2360, 1650 (ν, Py-C=N), 1604 (ν, Ar-C), 1574, 1549, 1504, 1486, 1408, 1312, 1291, 1224, 1187, 1157, 1100, 1059, 1022, 854, 807, 795, 743, 719, 701, 655, 629, 599, 581, 550, 516, 484.

14-A) *N*-(2,4-Dichlorophenyl)pyridin-4-amine (Table 1, Entry 14)

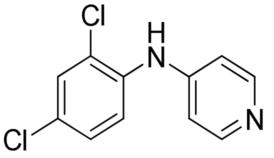

State: Green solid; Molecular formula: $C_{11}H_8Cl_2N_2$. Melting point: 206 °C (acetonitrile). HRMS (APCI) *m*/*z* calculated/found: $[M + H]^+$ = 239.0137/239.0142. ^{1}H-NMR (500 MHz, DMSO) δ 8.54 (s, 1H, NH), 8.20 (s, 2H, H_{Py}-2, H_{Py}-6), 7.43–7.70 (m, 4H, H_{Ar}), 6.78 (d, *J* = 6.0 Hz, 2H, H_{Py}-3, H_{Py}-5). ^{13}C-NMR (126 MHz, DMSO) δ 150.7 (2C, C_{Py}-2, C_{Py}-6), 150.5 (1C, C_{Py}-4), 136.9 (1C, $C_{Ar\text{-}NHPy}$), 130.1 (1C, C_{Ar}), 128.9 (1C, $C_{Ar\text{-}Cl}$), 128.8 (1C, C_{Ar}), 128.6 (1C, $C_{Ar\text{-}Cl}$), 126.2 (1C, C_{Ar}), 110.1 (2C, C_{Py}-3, C_{Py}-5). IR(KBr), ν (cm^{-1}) 3208 (ν, NH), 3135 (ν, NH), 2908, 1731, 1617, 1598 (ν, Ar-C=C), 1576, 1500, 1459, 1417, 1381, 1331, 1317, 1266, 1208, 1099, 1049, 994, 897, 864, 848, 816, 796, 727, 696, 673, 655, 618, 550.

14-B) *N*-(2,4-Dichlorophenyl)-*N*-(pyridin-4-yl)pyridin-4-amine (Table 1, Entry 14)

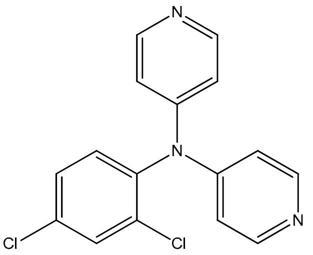

State: Yellowish viscus liquid; Molecular formula: $C_{16}H_{11}Cl_2N_3$. HRMS (APCI) *m*/*z* calculated/found: $[M + H]^+$ = 316.0403/316.0403. ^{1}H-NMR (500 MHz, $CDCl_3$) δ 8.59 (d, *J* = 6.2 Hz, 2H), 7.34 (t, *J* = 13.3 Hz, 1H), 7.29 (d, *J* = 2.3 Hz, 1H), 7.19 (dd, *J* = 8.0, 2.2 Hz, 1H), 7.15–7.10 (m, 1H), 7.09–7.00 (m, 1H), 6.75 (d, *J* = 8.5 Hz, 1H), 6.45 (dt, *J* = 17.1, 8.5 Hz, 1H), 5.89 (dd, *J* = 8.0, 2.7 Hz, 1H). ^{13}C-NMR (126 MHz, DMSO) δ ^{13}C-NMR (126 MHz, $CDCl_3$) δ 155.5, 150.8, 147.7, 146.5, 133.3, 132.3, 128.6, 126.7, 126.5, 126.0, 122.7, 116.3, 113.6, 108.1. IR(KBr), ν (cm^{-1}) 3039 (ν, Ar-H)), 2961, 2923, 2898, 2458, 1648, 1555 (ν, Ar-C=C), 1489, 1454, 1413, 1292, 1247, 1218, 1194, 1095, 1051, 1019, 878, 866, 810, 751, 733, 694, 635, 599.

15) *N*-(2,5-Dichlorophenyl)-*N*-(pyridin-4-yl)pyridin-4-amine (Table 1, Entry 15)

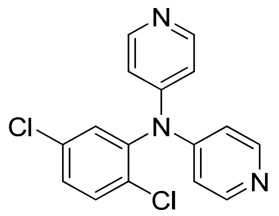

State: Yellow solid; Molecular formula: $C_{16}H_{11}Cl_2N_3$; Melting point: 174 °C (Acetonitrile). HRMS (APCI) *m*/z calculated/found: $[M + H]^+$ = 316.0403/316.0401. ^{1}H-NMR (300 MHz, $CDCl_3$) δ 8.68 (d, *J* = 6.2 Hz, 2H, H_{Py}-2, H_{Py}-6), 7.43 (d, *J* = 9.8 Hz, 1H, H_{Ar}), 7.29 (d, *J* = 9.0 Hz, 2H, H_{Py}-2, H_{Py}-6), 7.21 (d, *J* = 6.3 Hz, 2H, H_{Py}-3, H_{Py}-5), 6.92 (dd, *J* = 6.5, 2.4 Hz, 2H, H_{Py}-3, H_{Py}-5), 6.57 (dd, *J* = 7.9, 2.5 Hz, H_{Ar}), 6.00 (dd, *J* = 8.0, 2.5 Hz, H_{Ar}). ^{13}C-NMR (75 MHz, $CDCl_3$) δ 156.4, 151.9, 149.7, 148.7, 134.4, 133.5, 132.8, 130.8, 124.9, 123.32, 122.75, 117.3, 114.6, 109.2. IR(KBr), ν (cm^{-1}) 3097 (ν, Ar-H), 3080 (ν, Ar-H), 3058 (ν, Ar-H), 2958, 2927, 1727, 1653 (ν, Py-C=N), 1585 (ν, Ar-C=C), 1490, 1452, 1425, 1411, 1372, 1349, 1324, 1291, 1230, 1199, 1128, 1085, 1048, 1017, 1003, 911, 888, 841, 800, 732, 695, 627, 593, 583.

16-A) 4-[(Pyridin-4-yl)amino]benzonitrile (Table 1, Entry 16)

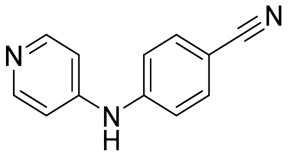

State: Pale yellow solid; Molecular formula: $C_{12}H_9N_3$. Melting point: 248 °C (dichlomethane:methanol, 9:1). HRMS (APCI) *m*/*z* calculated/found: $[M + H]^+$ = 196.0869/196.0872. ^{1}H-NMR (300 MHz, DMSO) δ 9.35 (s, 1H, NH), 8.34 (s, 2H, H_{Py}-2, H_{Py}-6), 7.72 (d, *J* = 8.8 Hz, 2H, H_{Ar}), 7.30 (d, *J* = 11.2 Hz, 2H, H_{Py}-3, H_{Py}-5), 7.08 (d, *J* = 6.1 Hz, 2H, H_{Ar}) [4]. ^{13}C-NMR (75 MHz, DMSO) δ 150.9 (2C, C_{Py}-2, C_{Py}-6), 148.5 (1C, C_{Py}-4), 146.0 (1C, $C_{Ar\text{-}NHPy}$), 134.2 (2C, C_{Ar}), 119.8 (2C, C_{Ar}), 118.2 (CN), 111.7 (2C, C_{Py}-3, C_{Py}-5), 102.9 (1C, $C_{Ar\text{-}CN}$). IR(KBr), ν (cm^{-1}) 3281 (ν, NH), 3170 (ν, NH), 2951, 2897, 2801, 2285, 2218 (ν, C≡N), 1644 (ν, Py-C=N), 1614, 1584 (ν, Ar-C=C), 1523, 1493, 1429, 1346, 1240, 1214, 1170, 1054, 997, 896, 855, 843, 821, 804, 722, 654, 612, 541.

16-B) 4-[(Dipyridin-4-yl)amino]benzonitrile (Table 1, Entry 16)

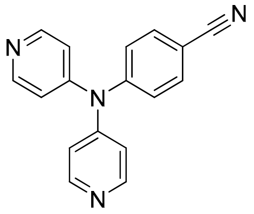

State: yellow solid; Molecular formula: $C_{17}H_{12}N_4$; Melting point: 208 °C (dichlomethane:methanol, 9:1). HRMS (APCI) *m*/*z* calculated/found: $[M + H]^+$ = 273.1135/273.1134. ^{1}H-NMR (300 MHz, DMSO) δ 8.68 (d, *J* = 6.2 Hz, 2H, H_{Py}-2, H_{Py}-6), 7.97 (d, *J* = 7.9 Hz, 2H, H_{Py}-2, H_{Py}-6), 7.72 (d, *J* = 8.6 Hz, 2H, H_{Ar}), 7.60 (d, *J* = 6.3 Hz, 2H, H_{Py}-3, H_{Py}-5), 6.98 (d, *J* = 8.5 Hz, 2H, H_{Py}-3, H_{Py}-5), 6.35 (d, *J* = 7.1 Hz, 2H, H_{Ar}). ^{13}C-NMR (75 MHz, DMSO) δ 156.2 (1C, C_{Py}-4), 155.7 (1C, C_{Py}-4), 151.8 (2C, C_{Py}-2, C_{Py}-6), 148.4 (2C, C_{Py}-2, C_{Py}-6), 136.6 (1C, $C_{Ar\text{-}N(Py)2}$), 134.0 (2C, C_{Ar}), 122.6 (2C, C_{Ar}), 120.1 (2C, C_{Py}-3, C_{Py}-5), 115.3 (CN), 104.1 (1C, $C_{Ar\text{-}CN}$). IR(KBr), ν (cm^{-1}) 3083 (ν, Ar-H), 3028 (ν, Ar-H), 2959, 2923, 2852, 2214 (ν, C≡N), 1738, 1649 (ν, Py-C=N), 1584 (ν, Ar-C=C), 1547, 1489, 1407, 1352, 1323, 1295, 1231, 1194, 1164, 1020, 855, 813, 794, 738, 693, 598, 544, 496.

17-A) 2-[(Pyridin-4-yl)amino]benzonitrile (Table 1, Entry 17)

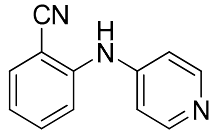

State: Brown solid; Molecular formula: $C_{12}H_9N_3$. Melting point: 164 °C (dichlomethane:methanol, 9:1). HRMS (APCI) m/z calculated/found: $[M + H]^+$ = 196.0869/196.0868. ^{1}H-NMR (500 MHz, $CDCl_3$) δ 8.47 (s, 2H, H_{Py}-2, H_{Py}-6), 7. 14–7.65 (m, 4H, H_{Ar}), 7.01 (s, 2H, H_{Py}-3, H_{Py}-5), 6.74 (s, 1H, NH). ^{13}C-NMR (126 MHz, $CDCl_3$) δ 150.8 (2C, C_{Py}-2, C_{Py}-6), 148.3 (1C, C_{Py}-4), 143.4 (1C, $C_{Ar\text{-}NHPy}$), 133.9 (1C, C_{Ar}), 133.6 (1C, C_{Ar}), 123.0 (1C, C_{Ar}), 119.1 (CN), 116.7 (1C, C_{Ar}), 111.6 (2C, C_{Py}-3, C_{Py}-5), 103.5 (1C, $C_{Ar\text{-}CN}$). IR(KBr), ν (cm^{-1}) 3239 (ν, NH), 3150 (ν, NH), 3072 (ν, Ar-H), 3045 (ν, Ar-H), 2959, 2912, 2223 (ν, C≡N), 1605, 1587 (ν, Ar-C=C), 1564, 1517, 1471, 1446, 1419, 1321, 1291, 1260, 1231, 1210, 1183, 1161, 1092, 1022, 997, 942, 898, 854, 808, 750, 711, 663, 634, 599, 547, 521 [9].

17-B) 4-[di(pyridin-4-yl)amino]benzonitrile (Table 1, Entry 17)

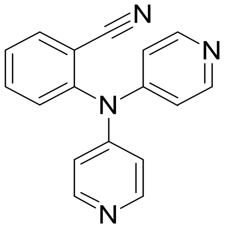

State: Pale yellow solid; Molecular formula: $C_{17}H_{12}N_4$. Melting point: 82 °C (dichlomethane:methanol, 9:1). HRMS (APCI) *m*/*z* calculated/found: $[M + H]^+$ = 273.1135/273.1137. ^{1}H-NMR (300 MHz, acetone) δ 8.69 (d, *J* = 5.9 Hz, 2H, H_{Py}-2, H_{Py}-6), 7.86 (d, *J* = 7.2 Hz, 2H, H_{Py}-2, H_{Py}-6), 7.56 -7.65 (m, 4H, H_{Ar}), 7.12 (td, *J* = 7.7, 0.9 Hz, 1H, H_{Py}-3, H_{Py}-5), 7.01 (d, *J* = 8.1 Hz, 1H, H_{Py}-3, H_{Py}-5), 6.37 (d, *J* = 88.7 Hz, 2H, 3PyH). ^{13}C-NMR (75 MHz, acetone) δ 156.5, 155.4, 151.5, 148.7, 133.7, 133.4, 122.4, 122.0, 117.8, 115.0, 105.6. IR(KBr), ν (cm^{-1}) 3042 (ν, Ar-H), 2962, 2927, 2631, 2457, 2374, 2218 (ν, C≡N), 1645 (ν, Py-C=N), 1587 (ν, Ar-C=C), 1565, 1544, 1489, 1471, 1439, 1411, 1350, 1322, 1290, 1261, 1222, 1195, 1155, 1097, 1019, 872, 797, 768, 733, 701, 548, 517, 484.

18-A) *N*-(3-Nitrophenyl)pyridin-4-amine (Table 1, Entry 18)

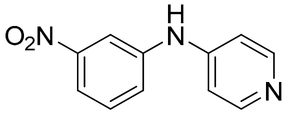

State: Bright Yellow solid; Molecular formula: $C_{11}H_9N_3O_2$. Melting point: 192 °C (acetonitrile) Lit. 182–184 °C (aq. methanol). HRMS (APCI) *m*/*z* calculated/found: $[M + H]^+$ = 216.0728/216.0731. ^{1}H-NMR (300 MHz, DMSO) δ 9.25 (s, 1H, NH), 8.31 (d, *J* = 4.5 Hz, 2H, H_{Py}-2, H_{Py}-6), 7.48–7.96 (m, 4H, H_{Ar}), 7.03 (d, *J* = 6.3 Hz, 2H, H_{Py}-3, H_{Py}-5) [10]. ^{13}C-NMR (75 MHz, DMSO) δ 150.9 (2C, C_{Py}-2, C_{Py}-6), 149.2 (1C, $C_{Ar\text{-}NO2}$), 149.1 (1C, C_{Py}-4), 142.7 (1C, $C_{Ar\text{-}NHPy}$), 131.2 (1C, C_{Ar}), 125.3 (1C, C_{Ar}), 116.6 (1C, C_{Ar}), 113.1 (2C, C_{Py}-3, C_{Py}-5), 110.7 (1C, C_{Ar}). IR(KBr), ν (cm^{-1}) 3254 (ν, NH), 3159 (ν, NH), 3056 (ν, Ar-H), 2801, 1621, 1592 (ν, Ar-C=C), 1524, 1498, 1475 (ν, N-O), 1439, 1421, 1337, 1231, 1212, 1083, 1055, 994, 949, 883, 856, 823, 801, 733, 698, 668, 629, 572 [11].

18-B) *N*-(3-Nitrophenyl)-*N*-(pyridin-4-yl)pyridin-4-amine (Table 1, Entry 18)

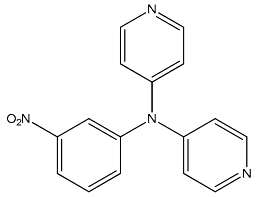

State: Bright red solid; Molecular formula: $C_{16}H_{12}N_4O_2$. Melting point: 92 °C (dichlomethane:methanol, 9:1). HRMS (APCI) *m*/*z* calculated/found: $[M + H]^+$ = 293.1033/293.1032. ^{1}H-NMR (300 MHz, DMSO) δ 8.68 (d, *J* = 5.2 Hz, 2H), 7.94 (d, *J* = 6.8 Hz, 2H), 7.83 (d, *J* = 8.2 Hz, 1H), 7.59 (m, 4H), 7.28 (d, *J* = 7.9 Hz, 1H), 6.35 (s, 2H). ^{13}C-NMR (126 MHz, DMSO) δ 156.4 (2C, C_{Py}-4), 151.8 (4C, C_{Py}-2, C_{Py}-6), 149.3 (1C, $C_{Ar\text{-}NO2}$), 148.4 (1C, $C_{Ar\text{-}NHPy}$), 131.0 (1C, C_{Ar}), 128.9 (s1C, C_{Ar}), 117.0 (1C, C_{Ar}), 115.9 (1C, C_{Ar}), 115.2 (4C, C_{Py}-3, C_{Py}-5s). IR(KBr), ν (cm^{-1}) 3383, 3323, 3230, 3087(ν, Ar-H), 3050(ν, Ar-H), 1647, 1589 (ν, Ar-C=C), 1546, 1514, 1493, 1462, 1414, 1353, 1324, 1297, 1220, 1201, 1028, 900, 818, 791, 731, 694, 649, 625, 577.

19-A) *N*-(4-Nitrophenyl)-*N*-(pyridin-4-yl)pyridin-4-amine (Table 1, Entry 19)

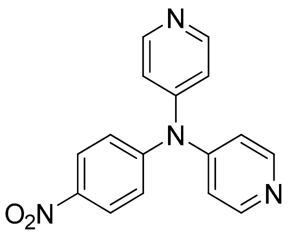

State: Bright red solid; Molecular formula: $C_{16}H_{12}N_4O_2$; Melting point: 172 °C (dichlomethane:methanol, 9:1). HRMS (APCI) *m*/*z* calculated/found: $[M + H]^+$ = 293.1033/293.1031. ^{1}H-NMR (300 MHz, DMSO) δ 8.68 (d, *J* = 6.3 Hz, 2H, H_{Py}-2, H_{Py}-6), 8.17 (d, *J* = 9.0 Hz, 2H, H_{Py}-2, H_{Py}-6), 7.61–7.96 (m, 4H, H_{Ar}), 6.98 (d, *J* = 9.0 Hz, 2H, H_{Py}-3, H_{Py}-5), 6.34 (d, *J* = 8.0 Hz, 2H, H_{Py}-3, H_{Py}-5). ^{13}C-NMR (75 MHz, DMSO) δ 159.9 (1C, $C_{Ar\text{-}N(Py)2}$), 156.0 (2C, C_{Py}-2, C_{Py}-6), 151.8 (2C, C_{Py}-2, C_{Py}-6), 148.3 (1C, C_{Py}-4), 141.7 (1C, C_{Py}-4), 136.4 (1C, $C_{Ar\text{-}NO2}$), 125.9 (2C, C_{Ar}), 122.0 (2C, C_{Ar}), 115.2 (2C, C_{Py}-3, C_{Py}-5), 112.8 (2C, C_{Py}-3, C_{Py}-5). IR(KBr), ν (cm^{-1}) 3085 (ν, Ar-H), 3051 (ν, Ar-H), 2963, 2895, 1650 (ν, Py-C=N), 1585 (ν, Ar-C=C), 1544, 1510, 1479 (ν, N-O), 1423, 1406, 1354, 1311, 1295, 1242, 1189, 1107, 1094, 1021, 860, 843, 804, 786, 753, 729, 697, 657, 627, 590, 542, 529.

19-B) *N*-(4-Nitrophenyl)pyridin-4-amine (Table 1, Entry 19)

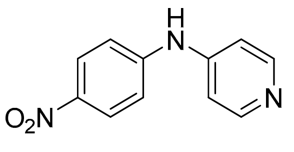

State: Yellow solid; Molecular formula: $C_{11}H_9N_3O_2$. Melting point: 295 °C (dichlomethane:methanol, 9:1) Lit. 298 °C (ethanol) [11]. HRMS (APCI) *m*/*z* calculated/found: $[M + H]^+$ = 216.0768/216.0767. ^{1}H-NMR (300 MHz, DMSO) δ 9.63 (s, 1H, NH), 8.39 (d, *J* = 4.8 Hz, 2H, H_{Py}-2, H_{Py}-6), 8.18 (d, *J* = 9.2 Hz, 2H, H_{Ar}), 7.33 (d, *J* = 9.2 Hz, 2H, H_{Py}-3, H_{Py}-5), 7.15 (d, *J* = 6.2 Hz, 2H, H_{Ar}). ^{13}C-NMR (75 MHz, DMSO) δ 151.0 (2C, C_{Py}-2, C_{Py}-6), 148.3 (1C, C_{Py}-4), 148.0 (1C, $C_{Ar\text{-}NHPy}$), 140.6 (1C, $C_{Ar\text{-}NO2}$), 126.2 (2C, C_{Ar}), 117.1 (2C, C_{Ar}), 112.5 (2C, C_{Py}-3, C_{Py}-5). IR(KBr), ν (cm^{-1}) 3279, 3221(ν, NH), 3175 (ν, NH), 3068 (ν, Ar-H), 2957, 2927, 2852 (ν, C-H), 2760, 1727, 1638, 1574 (ν, Ar-C=C), 1533, 1487 (ν, N-O), 1431, 1355, 1305, 1244, 1213, 1178, 1107, 1056, 998, 897, 860, 845, 817, 784, 745, 694, 652, 631, 601.

20) *N*-(Naphthalen-2-yl)pyridin-4-amine (Table 1, Entry 19)

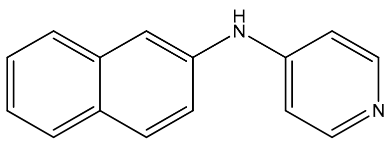

State: White solid; Molecular formula: $C_{15}H_{12}N_2$. Melting point: 223 °C (acetonitrile) Lit. 220 °C (dimethylformamide, water) [12]. HRMS (APCI) *m*/*z* calculated/found: $[M + H]^+$ = 221.1073/221.1073. ^{1}H-NMR (500 MHz, DMSO) δ 9.00 (s, 1H, NH), 8.25 (s, 2H, H_{Py}-2, H_{Py}-6), 7.89 (d, *J* = 8.8 Hz, 1H, H_{Ar}), 7.84 (t, *J* = 7.2 Hz, 2H, H_{Ar}), 7.68 (d, *J* = 1.7 Hz, 1H, H_{Ar}), 7.47 (t, *J* = 7.5 Hz, 1H, H_{Ar}), 7.42–7.33 (m, 2H, H_{Ar}), 7.04 (d, *J* = 5.0 Hz, 2H, H_{Py}-3, H_{Py}-5). ^{13}C-NMR (126 MHz, DMSO) δ 150.7 (2C, C_{Py}-2, C_{Py}-6), 150.3 (1C, C_{Py}-4), 138.8 (1C, $C_{Ar\text{-}NHPy}$), 134.4 (1C, C_{Ar}), 129.8 (1C, C_{Ar}), 129.5 (1C, C_{Ar}), 128.0 (1C, C_{Ar}), 127.3 (1C, C_{Ar}), 127.0 (1C, C_{Ar}), 124.7 (1C, C_{Ar}), 121.7 (1C, C_{Ar}), 114.9 (1C, C_{Ar}), 110.0 (2C, C_{Py}-3, C_{Py}-5). IR(KBr), ν (cm^{-1}) 3394, 3277 (ν, NH), 3047 (ν, Ar-H), 2995, 2833, 1623, 1582 (ν, Ar-C=C), 1524, 1505, 1488, 1467, 1390, 1339, 1311, 1249, 1212, 1178, 1122, 1058, 990, 963, 947, 922, 883, 856, 806, 744, 652, 597, 520.

21) 1-(Pyridin-4-yl)piperazine

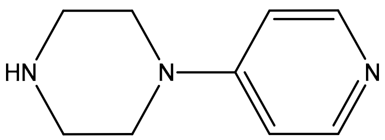

State: White solid; Molecular formula: $C_9H_{13}N_3$. Melting point: 140 °C (heptane) Lit .140 °C (heptane) [13]. HRMS (APCI) *m*/*z* calculated/found: $[M + H]^+$ = 164.1182/164.1182. ^{1}H-NMR (300 MHz, $CDCl_3$) δ 8.29–6.68 (m, 4H, H_{Py}), 3.52–3.18 (m, 4H, CH_2), 3.13–2.86 (m, 4H, CH_2) [13]. ^{13}C-NMR (75 MHz, $CDCl_3$) δ 155.3 (1C, C_{Py}-4), 150.3 (2C, C_{Py}-2, C_{Py}-6), 108.3 (2C, C_{Py}-3, C_{Py}-5), 47.0 (CH_2), 45.7 (CH_2). IR(KBr), ν (cm^{-1}) 3372, 3267 (ν, NH), 3212 (ν, NH), 2955 (ν, C-H), 2926 (ν, C-H), 2884 (ν, C-H), 2847 (ν, C-H), 2824, 2755, 2645, 2250, 1920, 1596 (ν, Ar-C=C), 1536, 1508, 1399, 1385, 1371, 1326, 1292, 1257, 1227, 1143, 1121, 1106, 1063, 990, 917, 851, 799, 733, 633, 601, 536.

22) 4-(4-Methoxyphenylamino)-1-methylpyridin-1-ium iodide

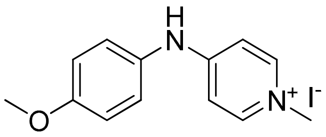

State: White solid; Molecular formula: $C_{13}H_{15}IN_2O$. Melting point: 110 °C. HRMS (APCI) *m*/*z* calculated/found: $[M + H]^+$ = 215.1179/215.1182. ^{1}H-NMR (300 MHz, D_2O) δ 8.05–7.37 (m, 4H, H_{Py}), 7.04–7.17 (m, 4H, H_{Ar}), 4.00 (s, 3H, CH_3), 3.94 (s, 3H, H_{OCH3}). ^{13}C-NMR (126 MHz, MeOD) δ 160.06 (1C, C_{Py}-4), 158.93 (1C, $C_{Ar\text{-}OCH3}$), 156.59 (2C, C_{Py}-2, C_{Py}-6), 143.61 (1C, $C_{Ar\text{-}NHPy}$), 129.58 (2C, C_{Ar}), 125.54 (2C, C_{Py}-3, C_{Py}-5), 114.90 (2C, C_{Ar}), 54.66 (1C, OCH_3), 43.87 (1C, CH_3). IR(KBr), ν (cm^{-1}) 3052 (ν, Ar-H), 2969 (ν, C-H), 2941 (ν, C-H), 1650 (ν, Py-C=N), 1607 (ν, Ar-C=C), 1541, 1508, 1458, 1357, 1304, 1247, 1216, 1198, 1169, 1111, 1027, 994, 956, 898, 875, 817, 773, 754, 705, 676, 658, 641, 625, 585, 521.

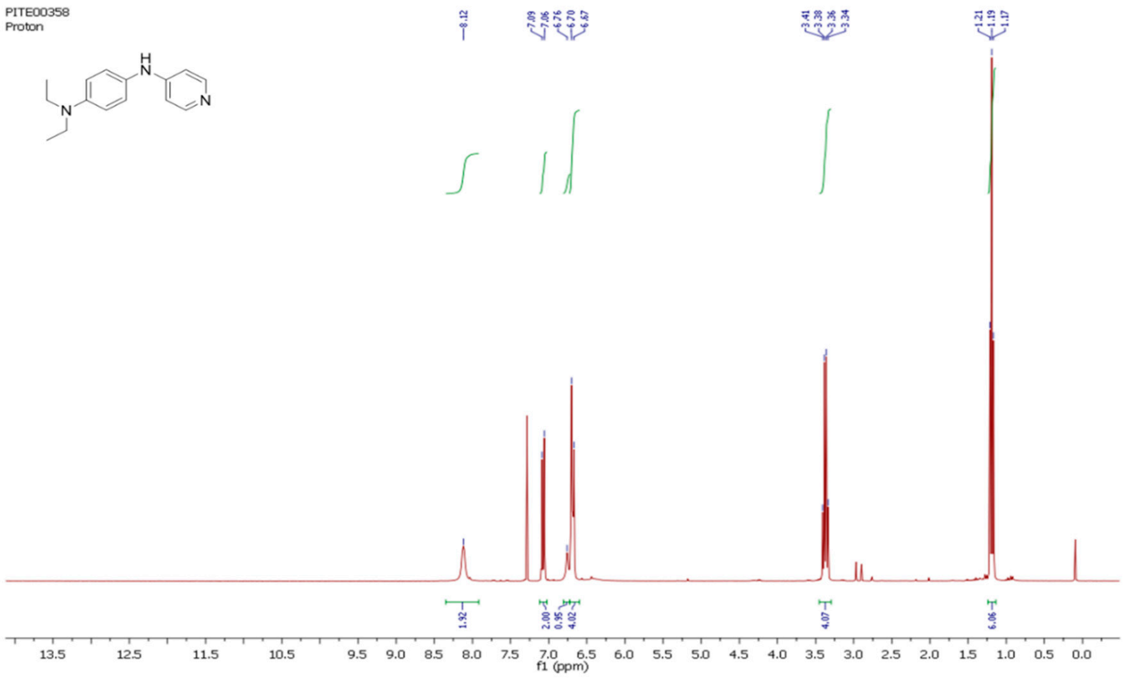

Figure S1. ^{1}H-NMR for *N*^{1},*N*^{1}-diethyl-*N*^{4}-(pyridin-4-yl)benzene-1,4-diamine.

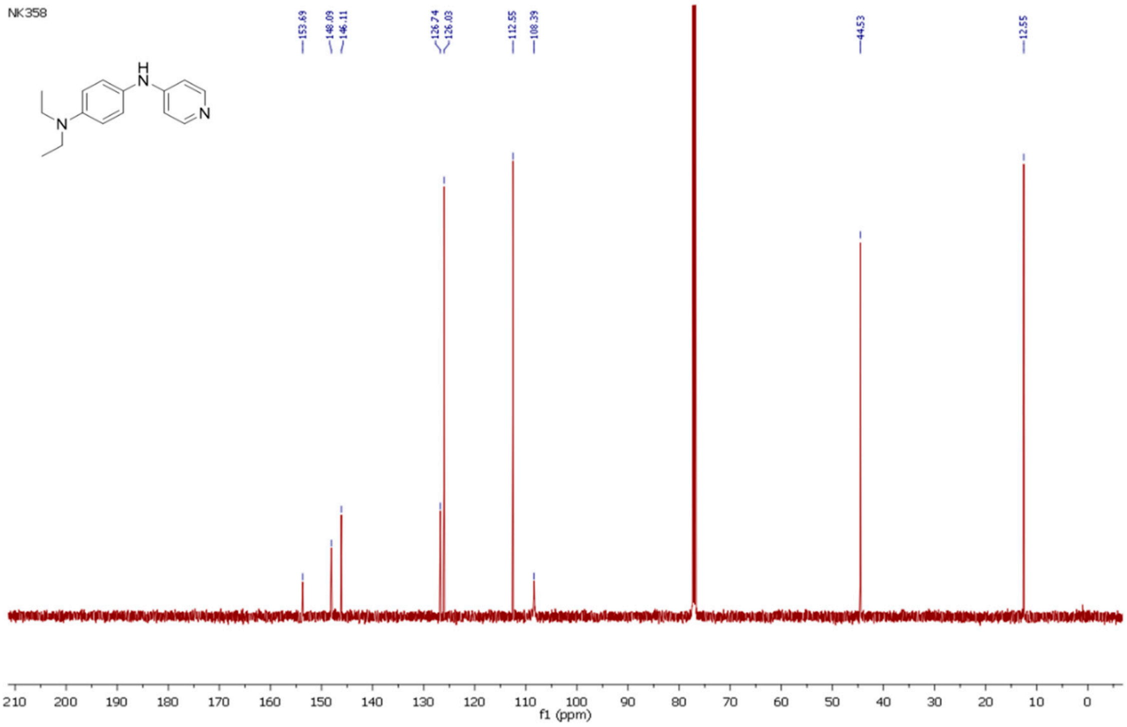

Figure S2. ^{13}C-NMR for *N*^{1},*N*^{1}-diethyl-*N*^{4}-(pyridin-4-yl)benzene-1,4-diamine.

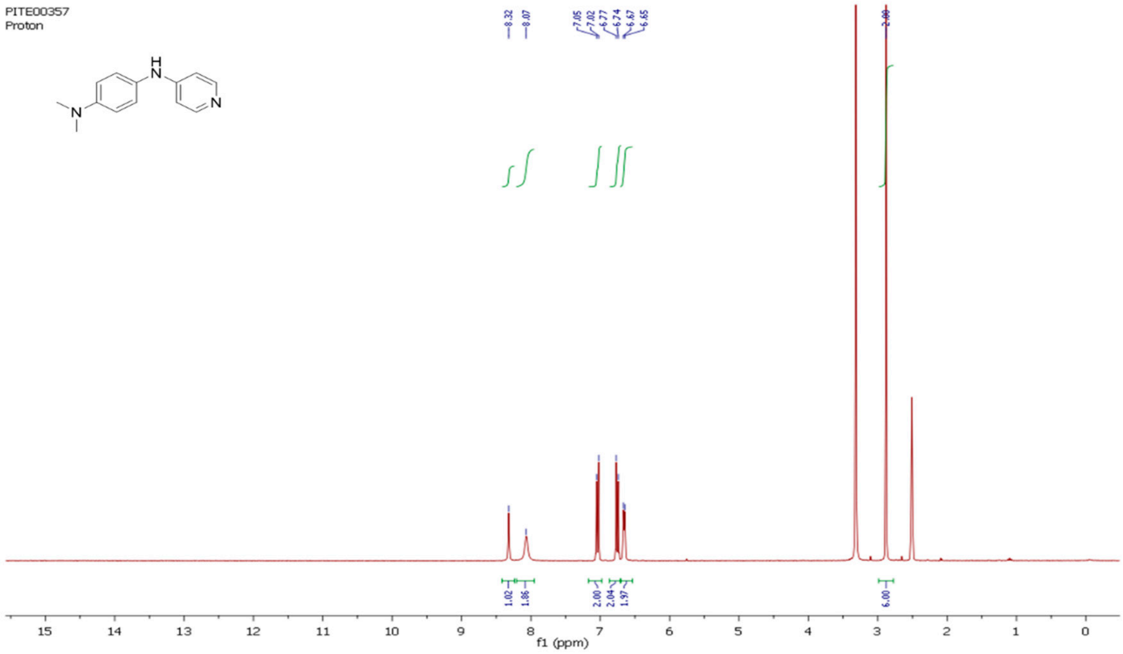

Figure S3. ^{1}H-NMR for *N*[1],*N*[1]-dimethyl-*N*[4]-(pyridin-4-yl)benzene-1,4-diamine.

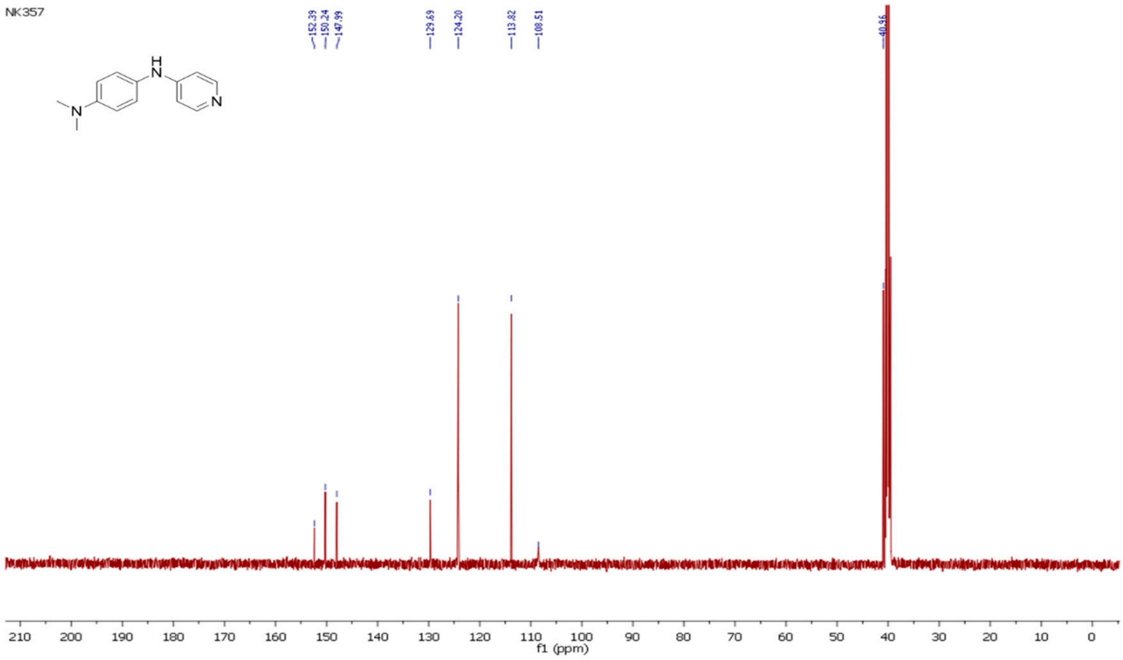

Figure S4. ^{13}C-NMR for *N*[1],*N*[1]-dimethyl-*N*[4]-(pyridin-4-yl)benzene-1,4-diamine.

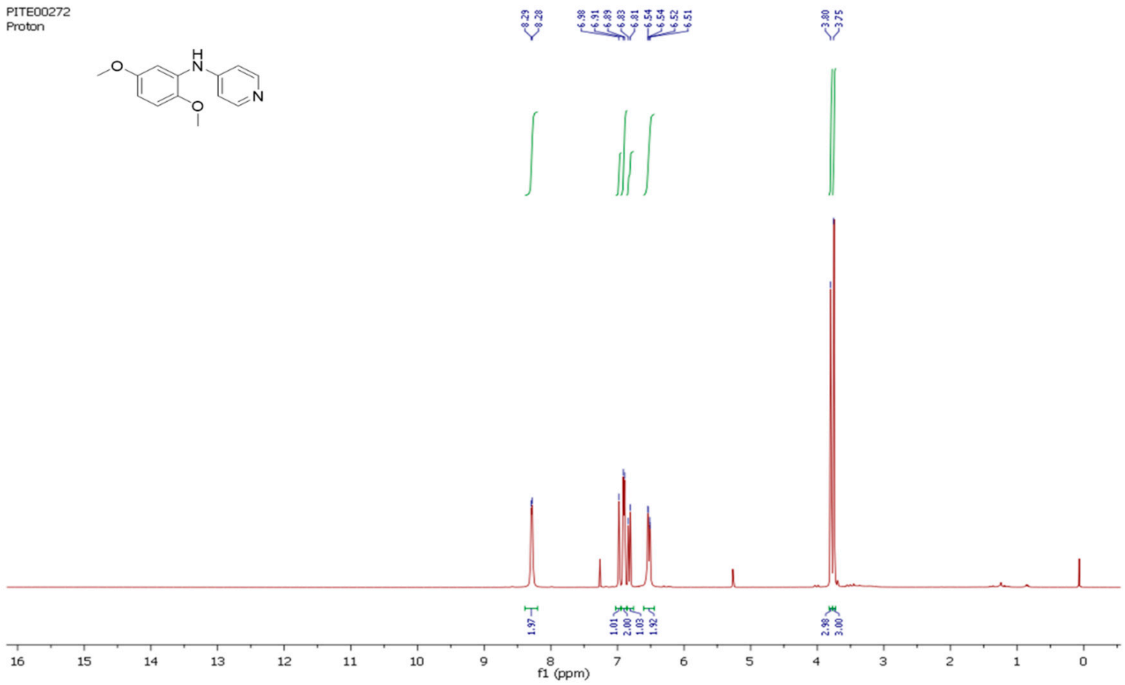

Figure S5. ^{1}H-NMR for *N*-(2,5-dimethoxyphenyl)pyridin-4-amine.

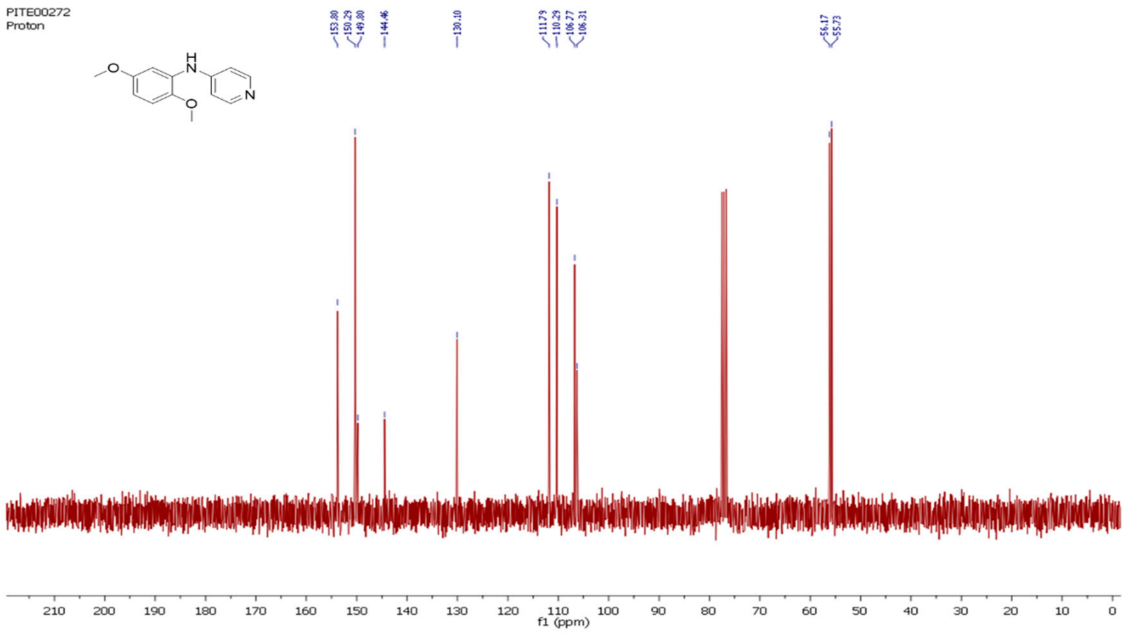

Figure S6. ^{13}C-NMR for *N*-(2,5-dimethoxyphenyl)pyridin-4-amine.

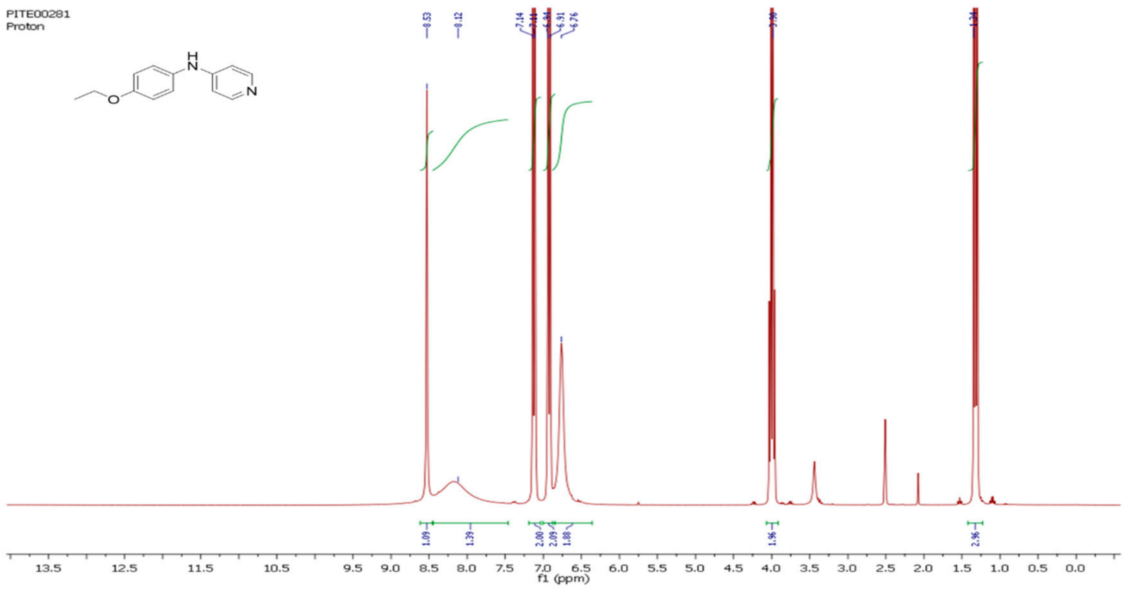

Figure S7. ^{1}H-NMR for *N*-(4-ethoxyphenyl)pyridin-4-amine.

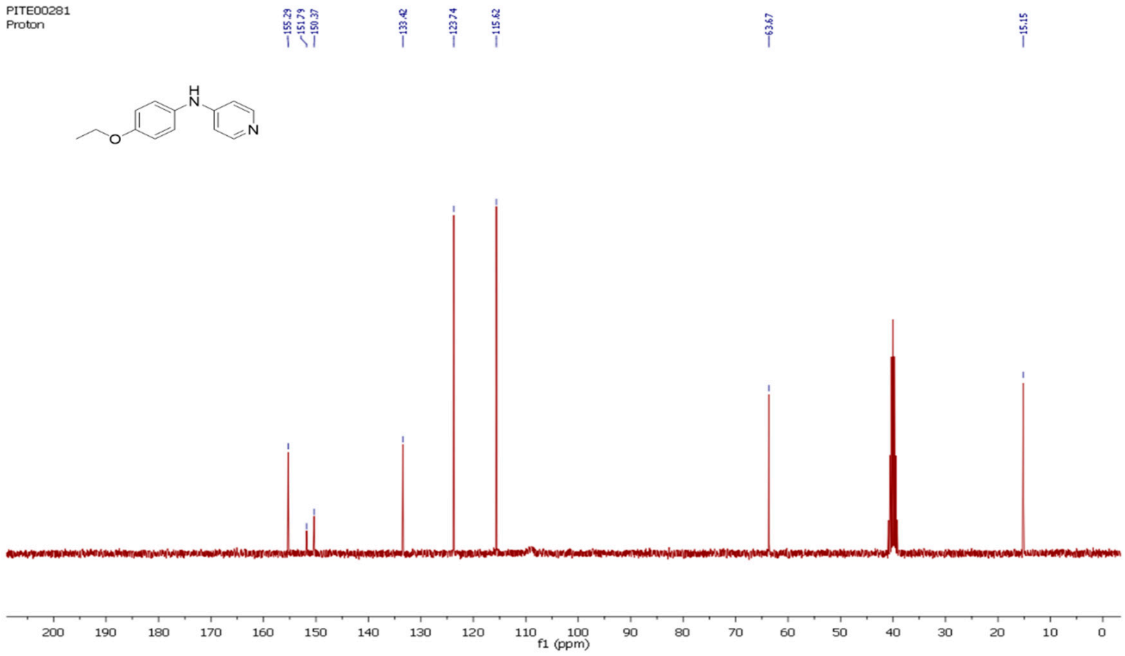

Figure S8. ^{13}C-NMR for *N*-(4-ethoxyphenyl)pyridin-4-amine.

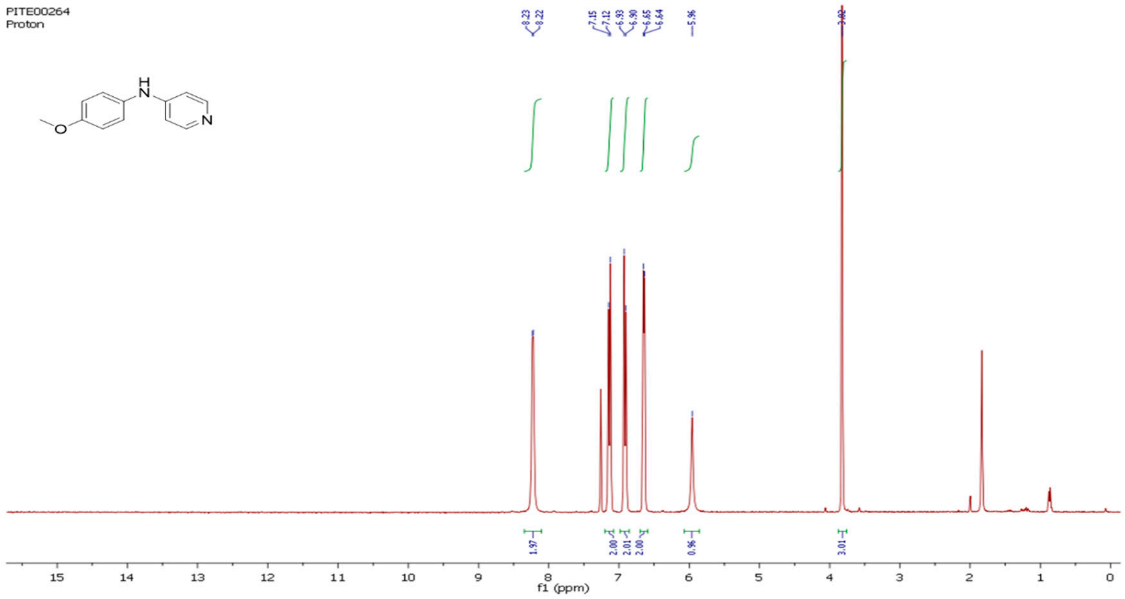

Figure S9. ^{1}H-NMR for *N*-(4-methoxyphenyl)pyridin-4-amine.

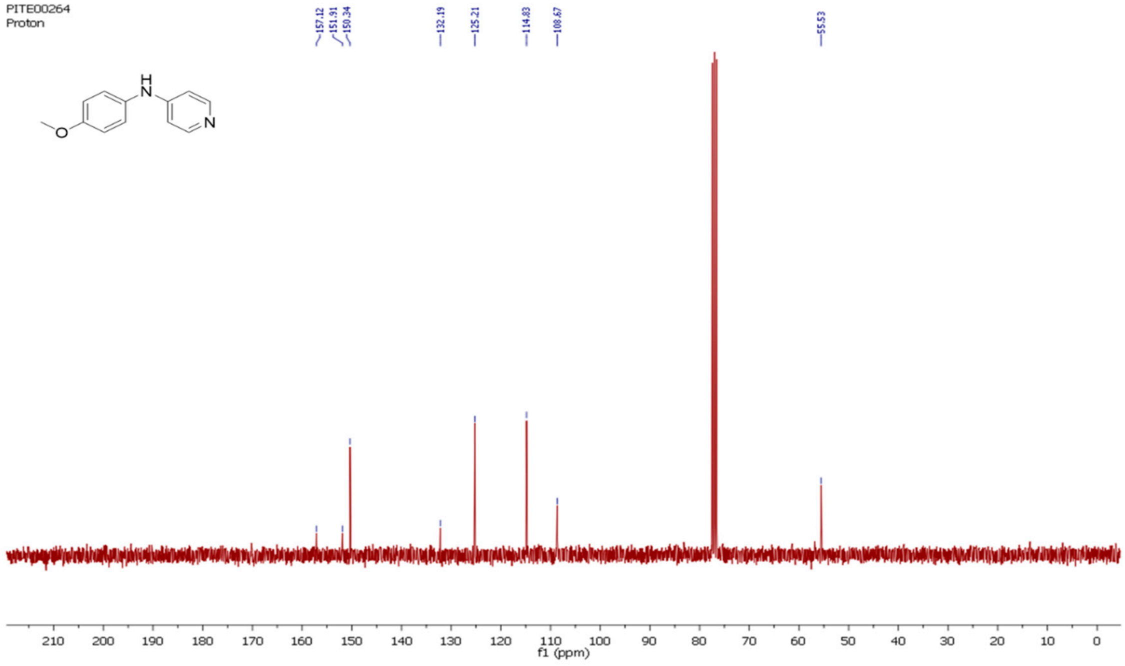

Figure S10. ^{13}C-NMR for *N*-(4-methoxyphenyl)pyridin-4-amine.

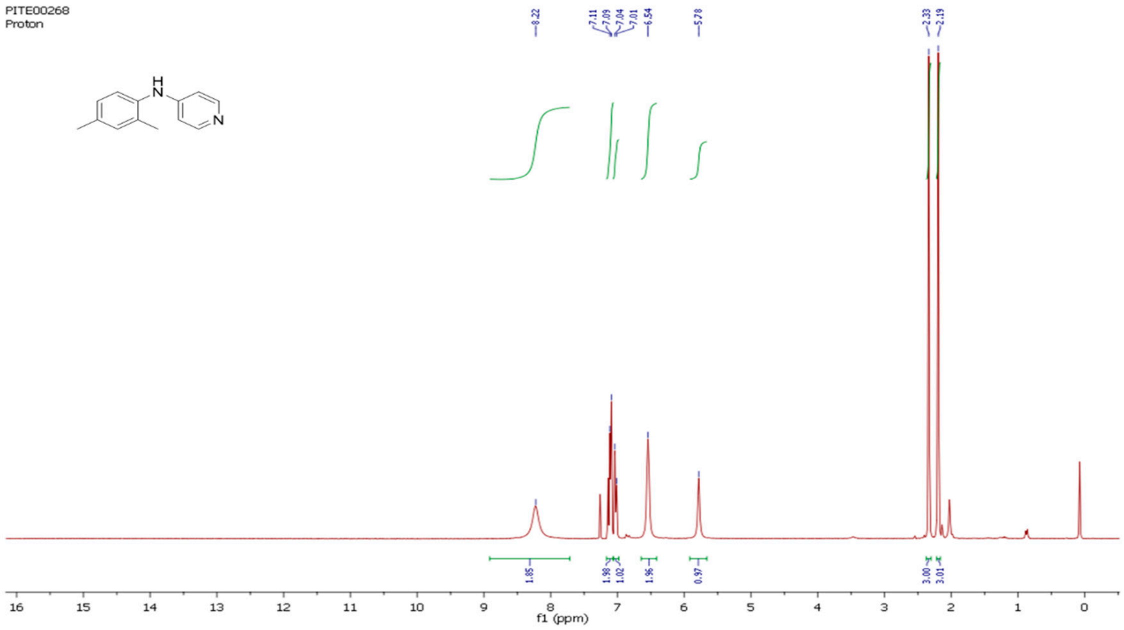

Figure S11. ^{1}H-NMR for *N*-(2,4-dimethylphenyl)pyridin-4-amine.

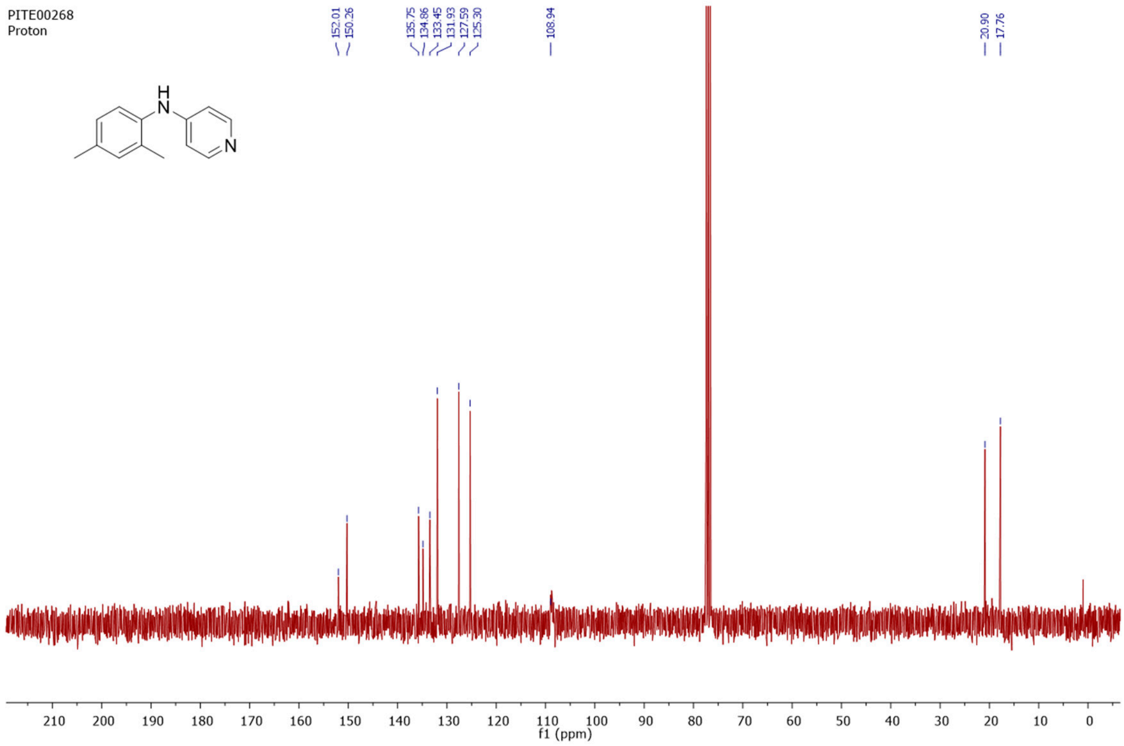

Figure S12. ^{13}C-NMR for *N*-(2,4-dimethylphenyl)pyridin-4-amine.

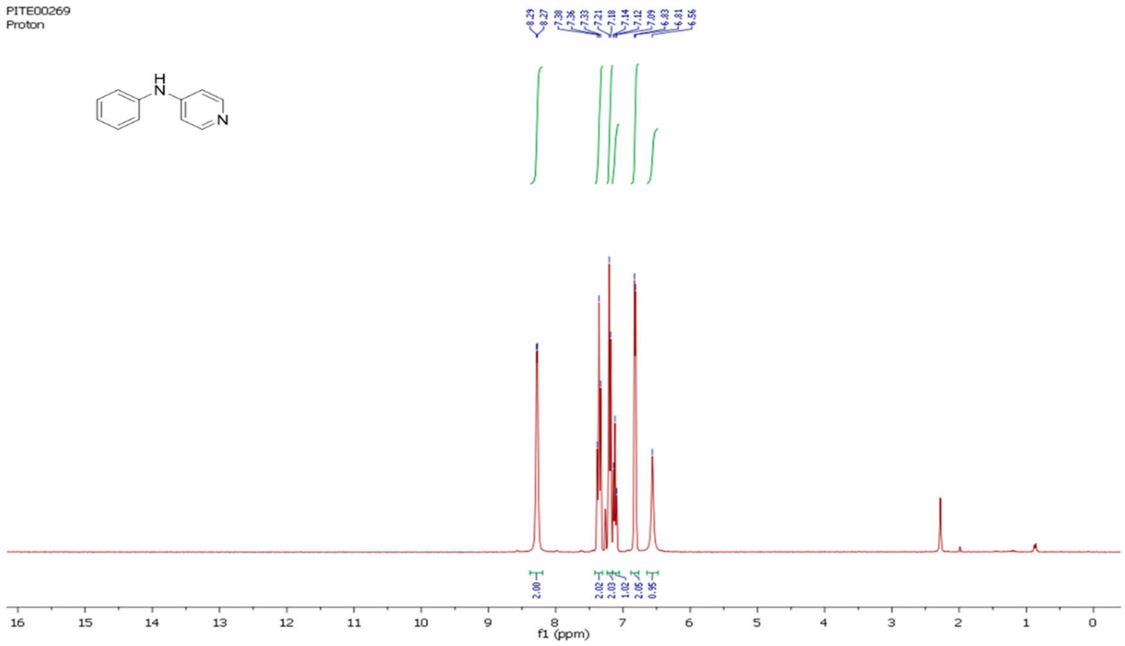

Figure S13. ^{1}H-NMR for *N*-phenylpyridin-4-amine.

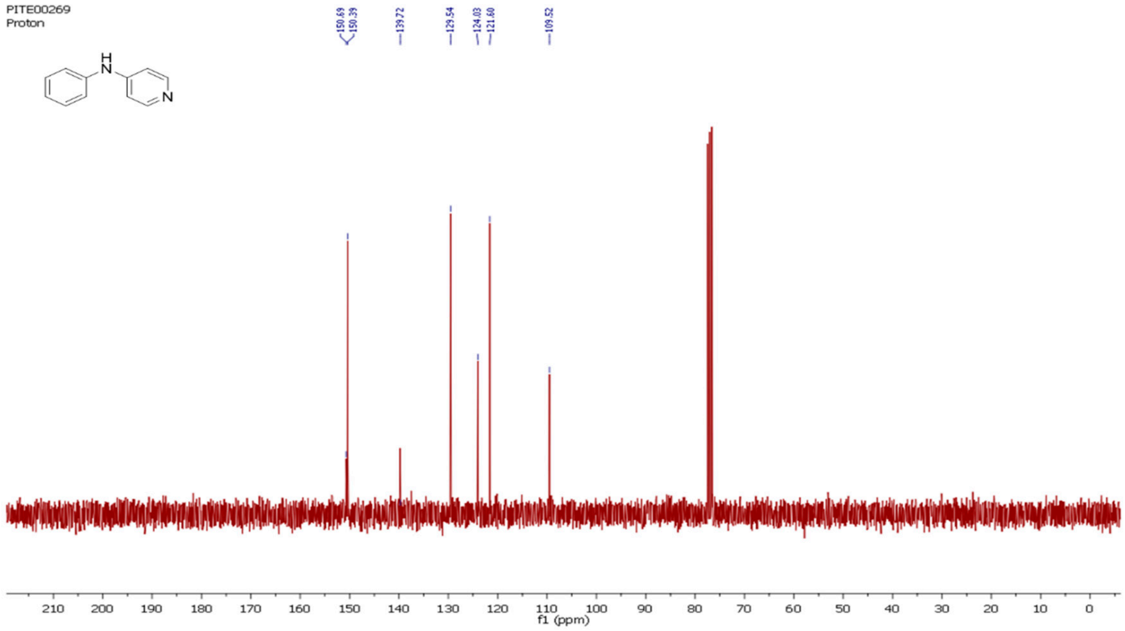

Figure S14. ^{13}C-NMR for *N*-phenylpyridin-4-amine.

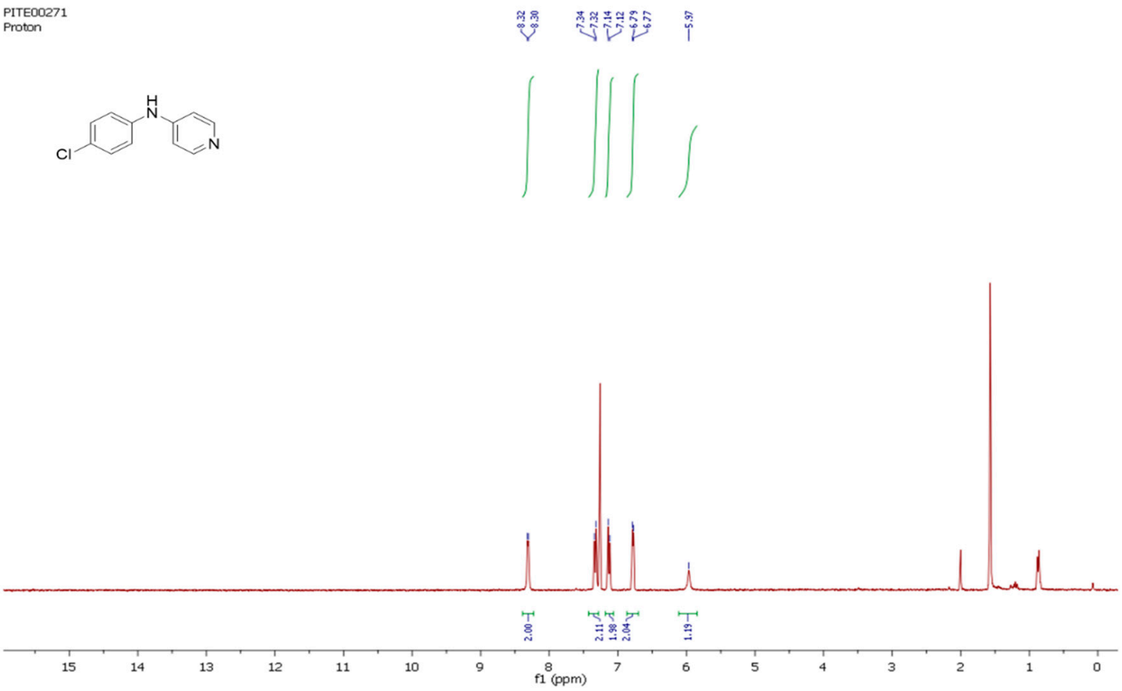

Figure S15. ^{1}H-NMR for *N*-(4-chlorophenyl)pyridin-4-amine.

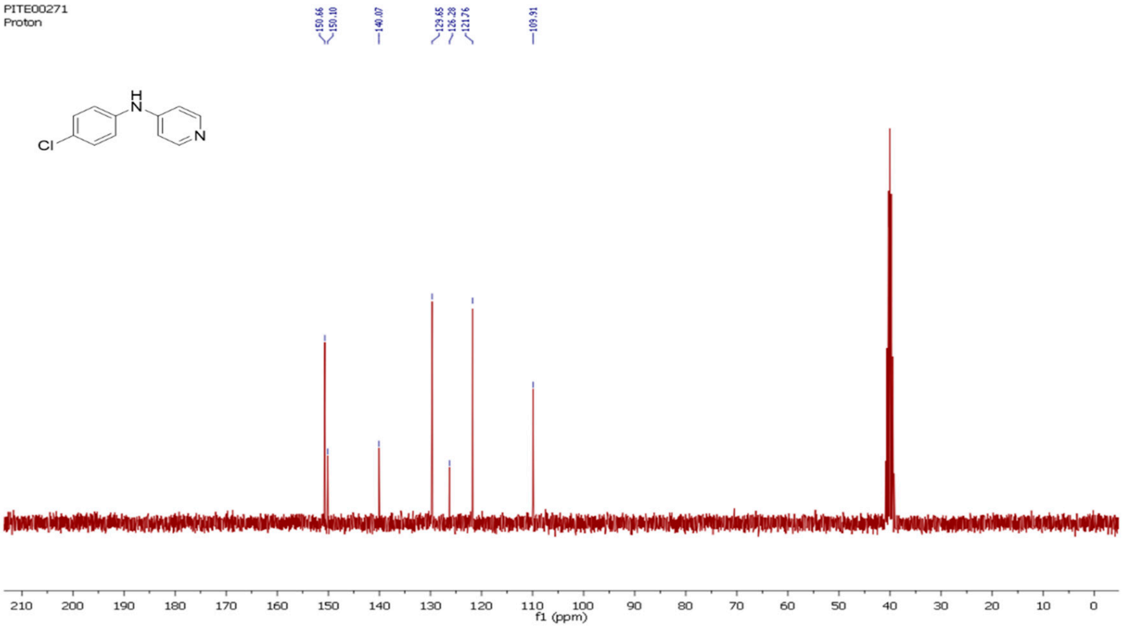

Figure S16. ^{13}C-NMR for *N*-(4-chlorophenyl)pyridin-4-amine.

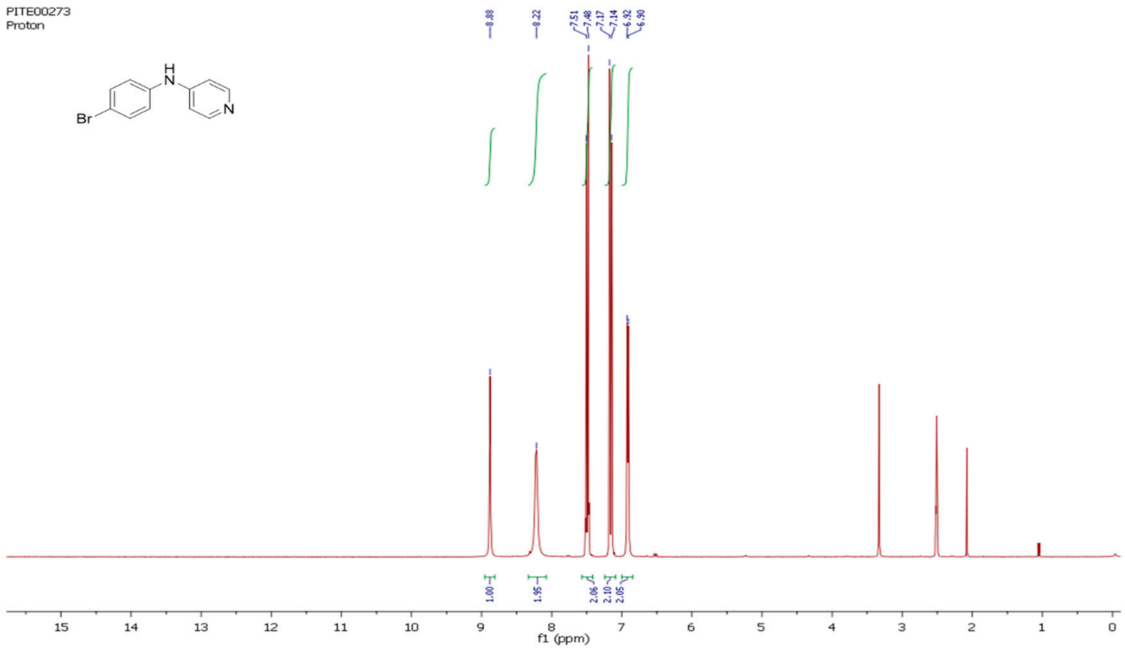

Figure S17. ^{1}H-NMR for *N*-(4-bromophenyl)pyridin-4-amine.

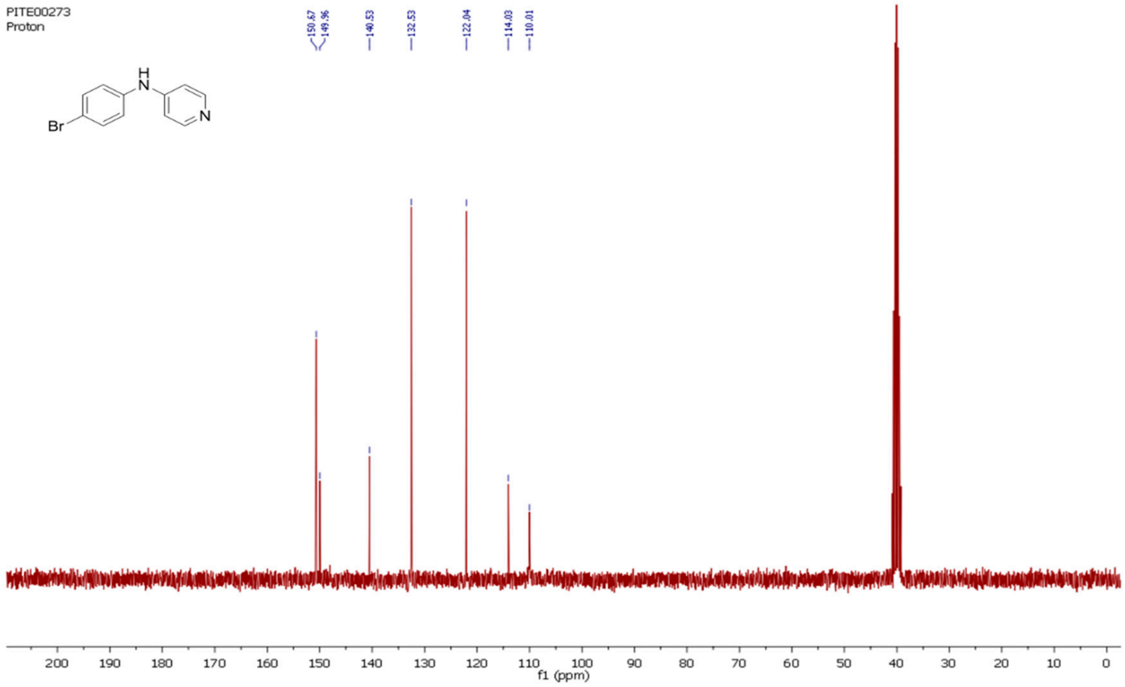

Figure S18. ^{13}C-NMR for *N*-(4-bromophenyl)pyridin-4-amine.

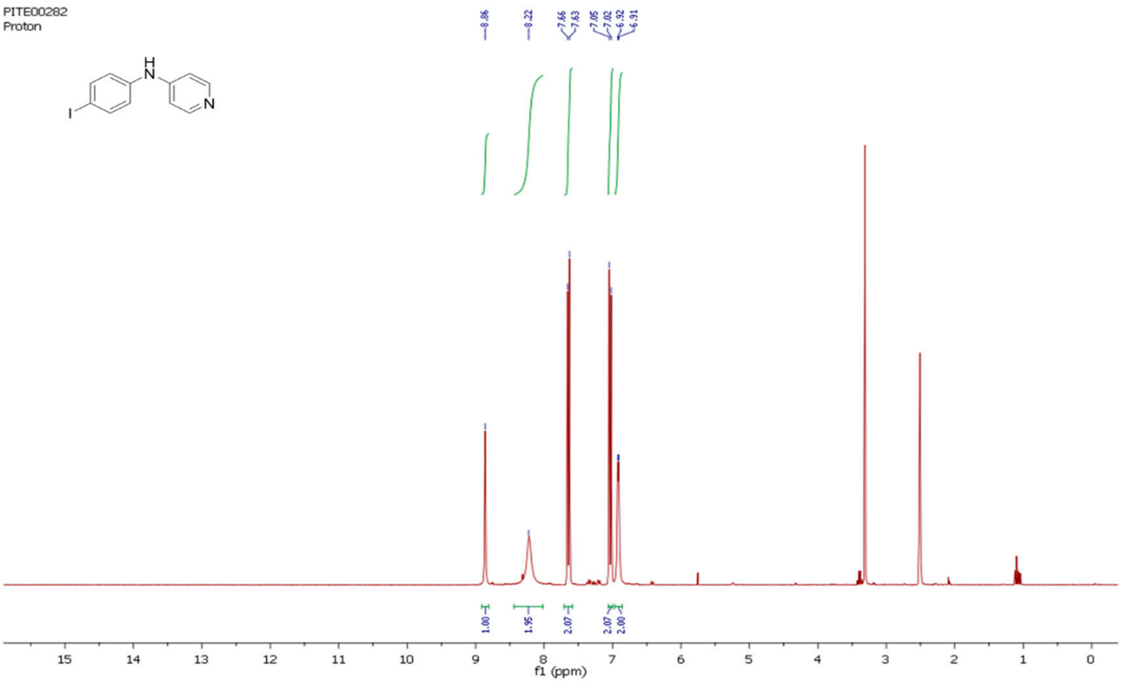

Figure S19. ^{1}H-NMR for *N*-(4-bromophenyl)pyridin-4-amine.

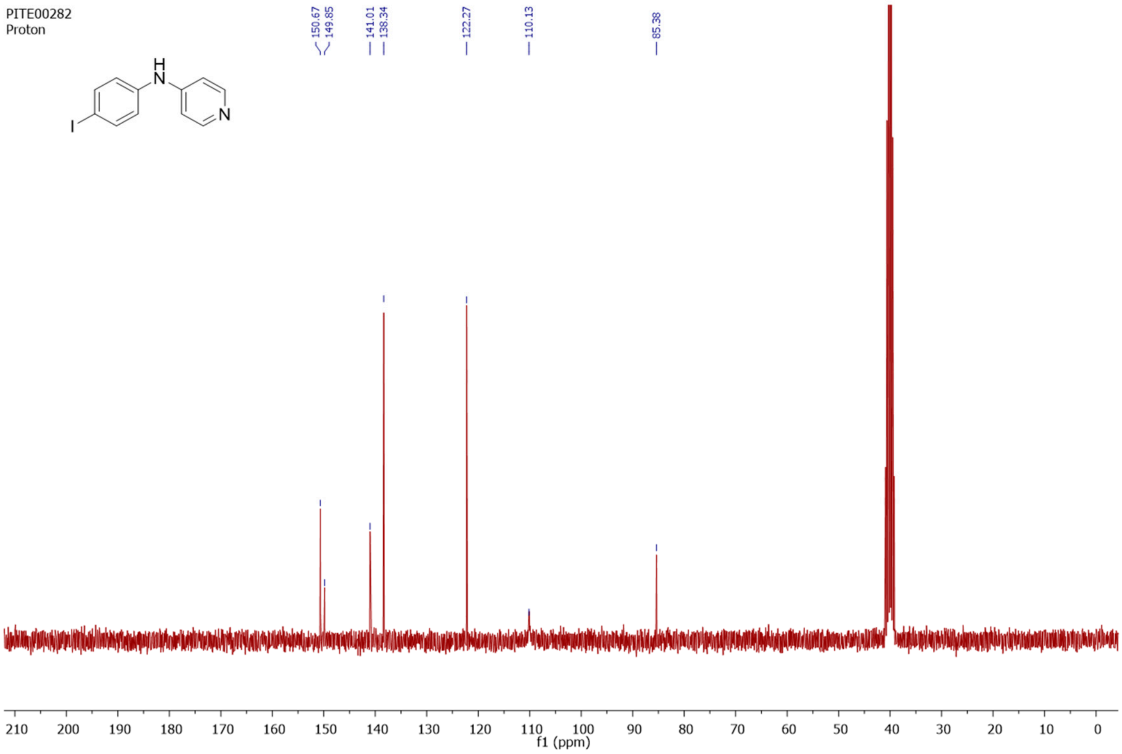

Figure S20. ^{13}C-NMR for *N*-(4-bromophenyl)pyridin-4-amine.

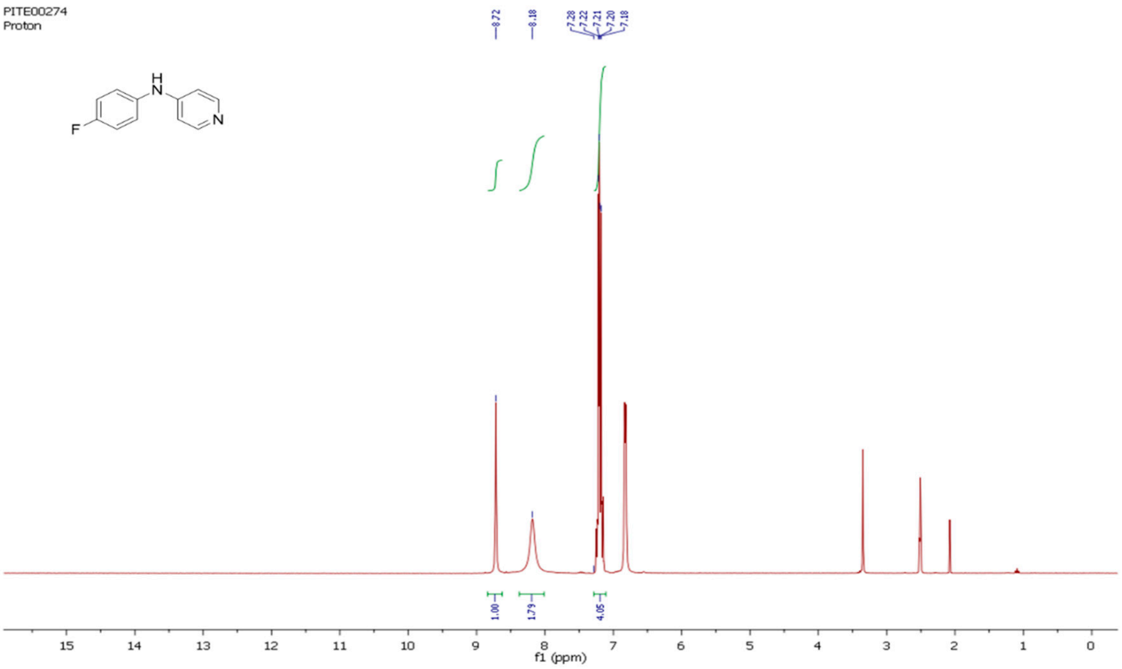

Figure S21. ^{1}H-NMR for *N*-(4-fluorophenyl)pyridin-4-amine.

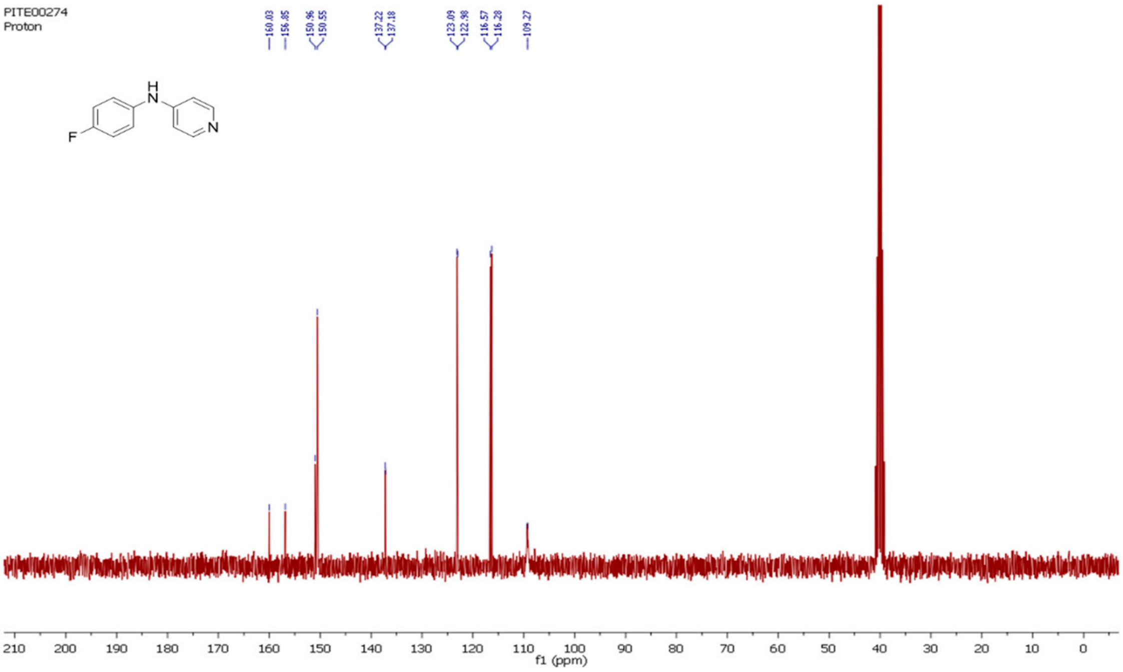

Figure S22. ^{13}C-NMR for *N*-(4-fluorophenyl)pyridin-4-amine.

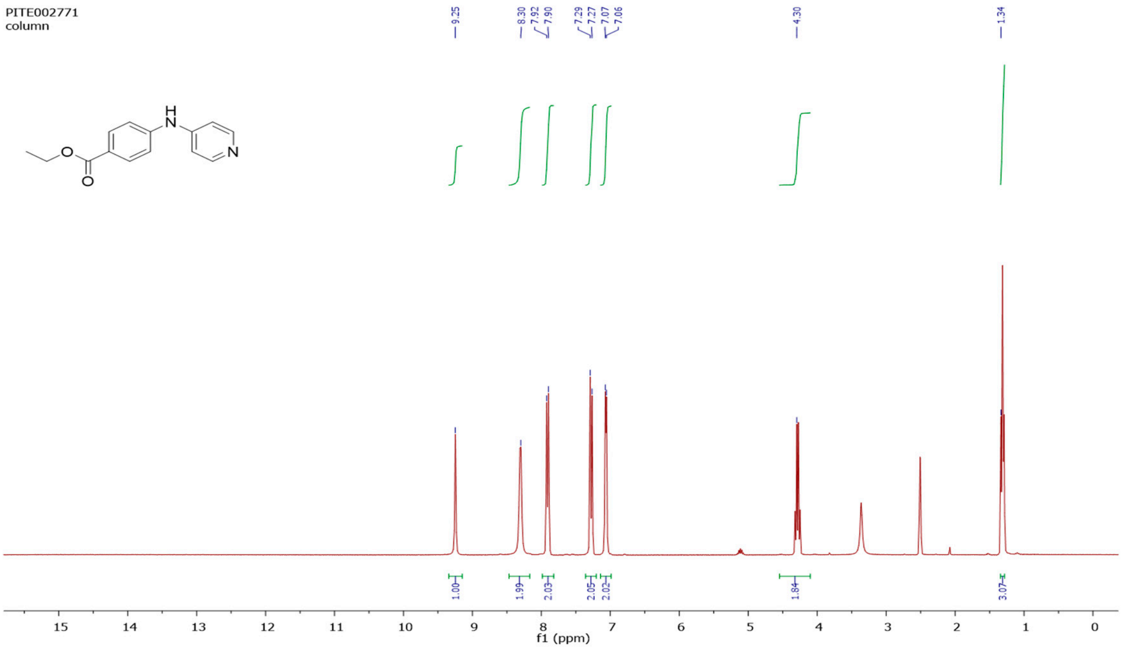

Figure S23. ^{1}H-NMR for ethyl 4-[(pyridin-4-ylamino)]benzoate.

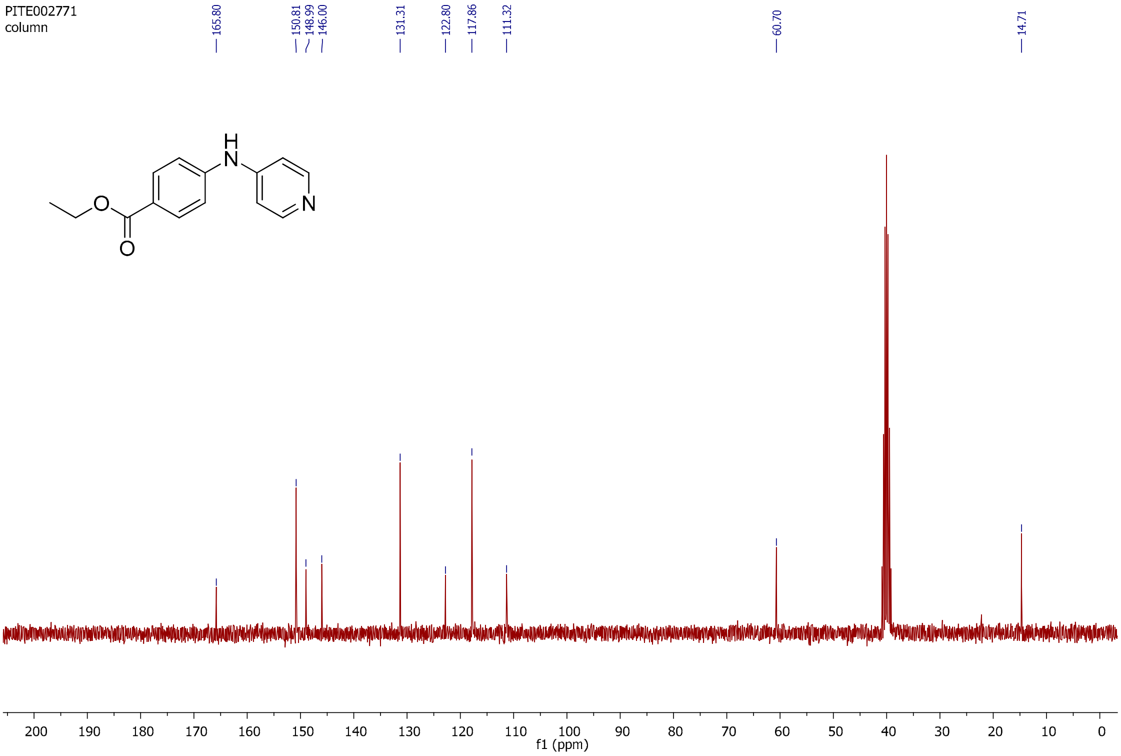

Figure S24. ^{13}C-NMR for ethyl 4-[(pyridin-4-ylamino)]benzoate.

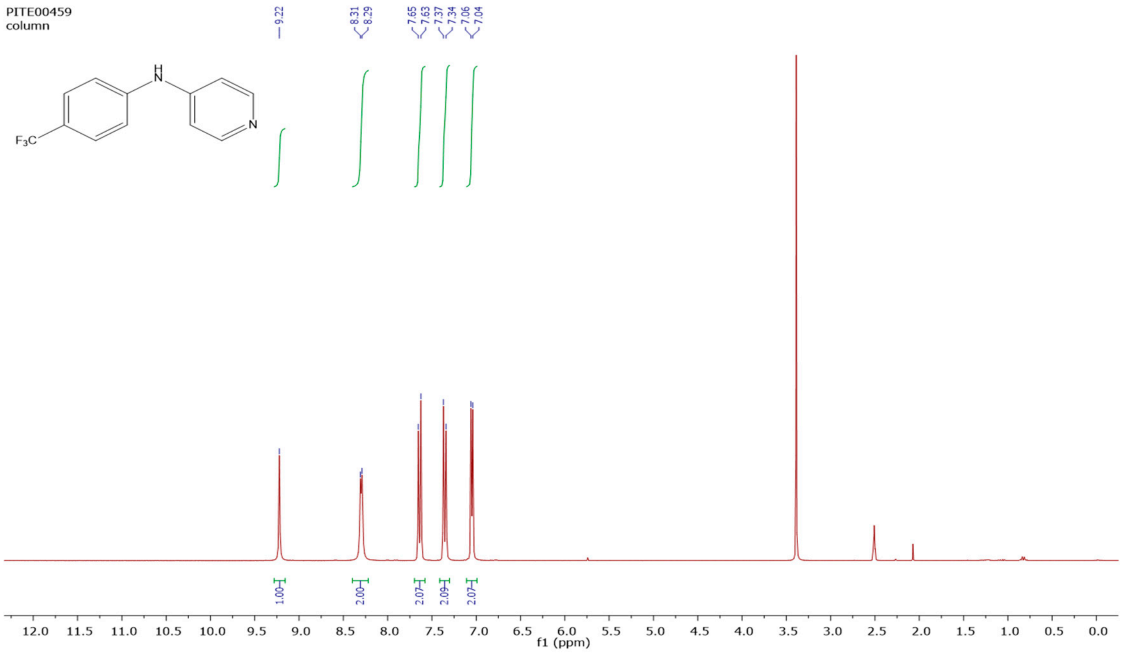

Figure S25. ^{1}H-NMR for *N*-(4-(trifluoromethyl)phenyl)pyridin-4-amine.

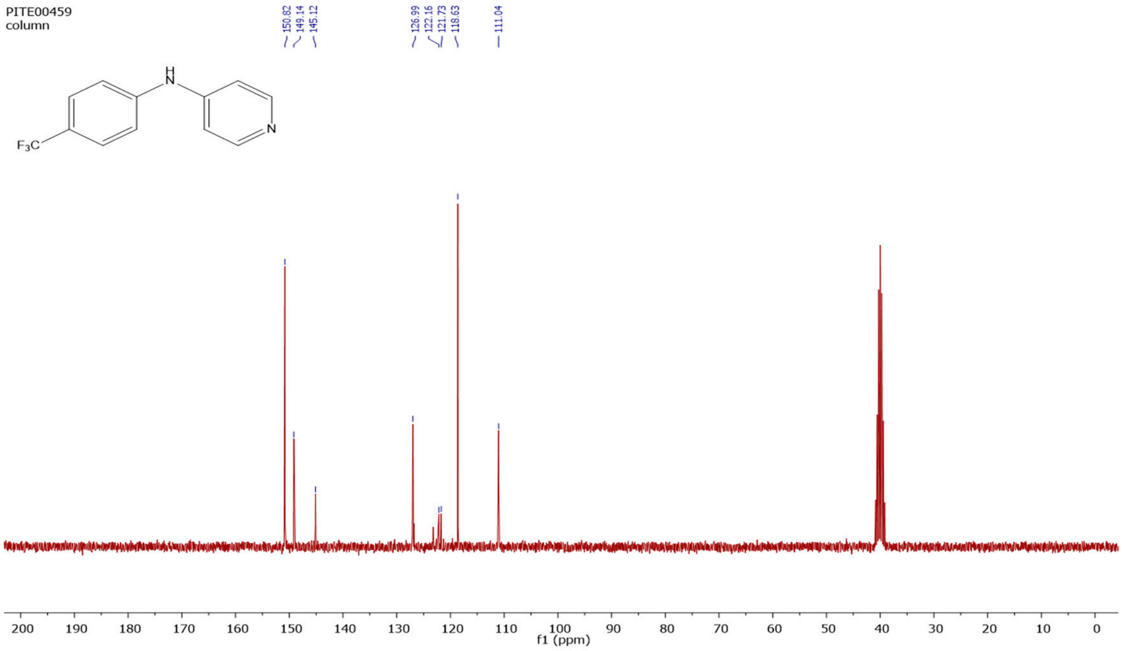

Figure S26. ^{13}C-NMR for *N*-(4-(trifluoromethyl)phenyl)pyridin-4-amine.

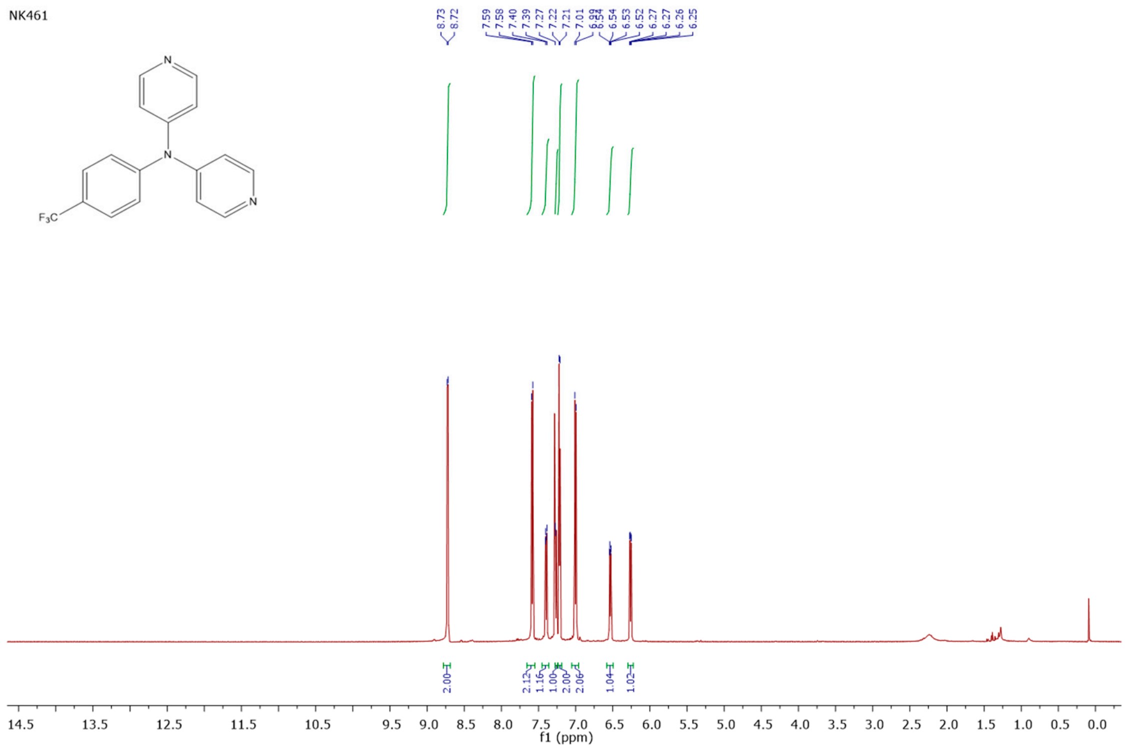

Figure S27. ^{1}H-NMR for *N*-(pyridin-4-yl)-*N*-[4-(trifluoromethyl)phenyl]pyridin-4-amine.

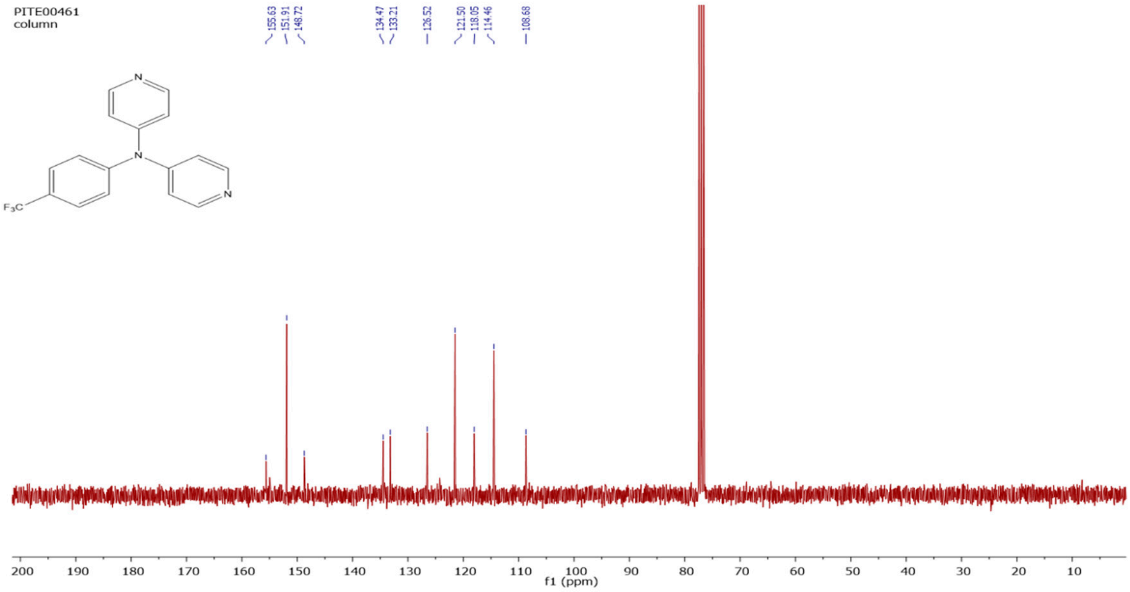

Figure S28. ^{13}C-NMR for *N*-(pyridin-4-yl)-*N*-[4-(trifluoromethyl)phenyl]pyridin-4-amine.

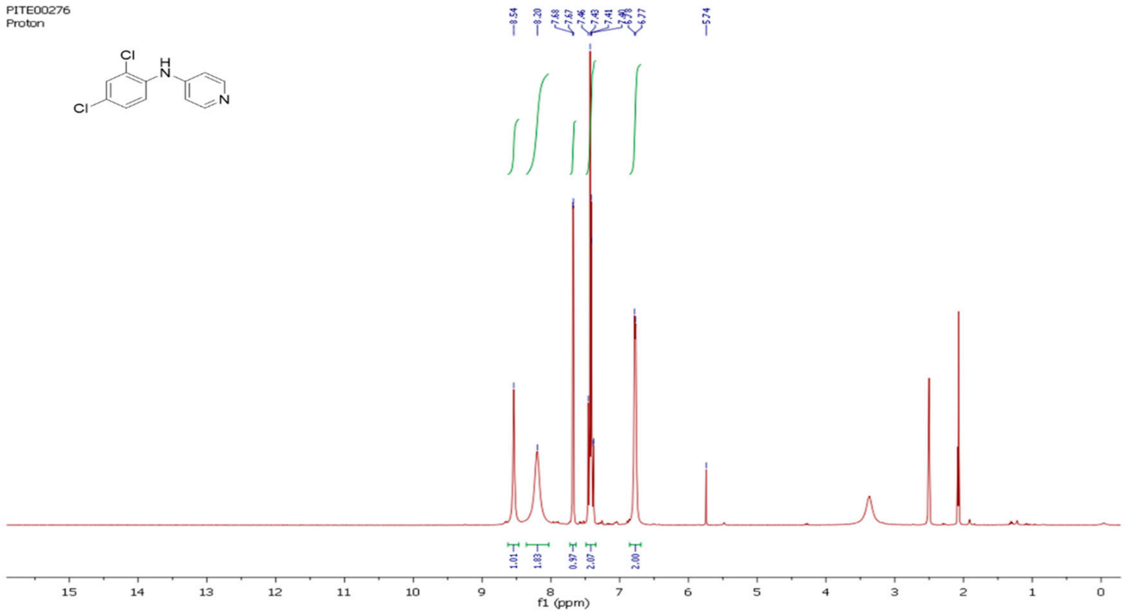

Figure S29. ^{1}H-NMR for *N*-(2,4-dichlorophenyl)pyridin-4-amine.

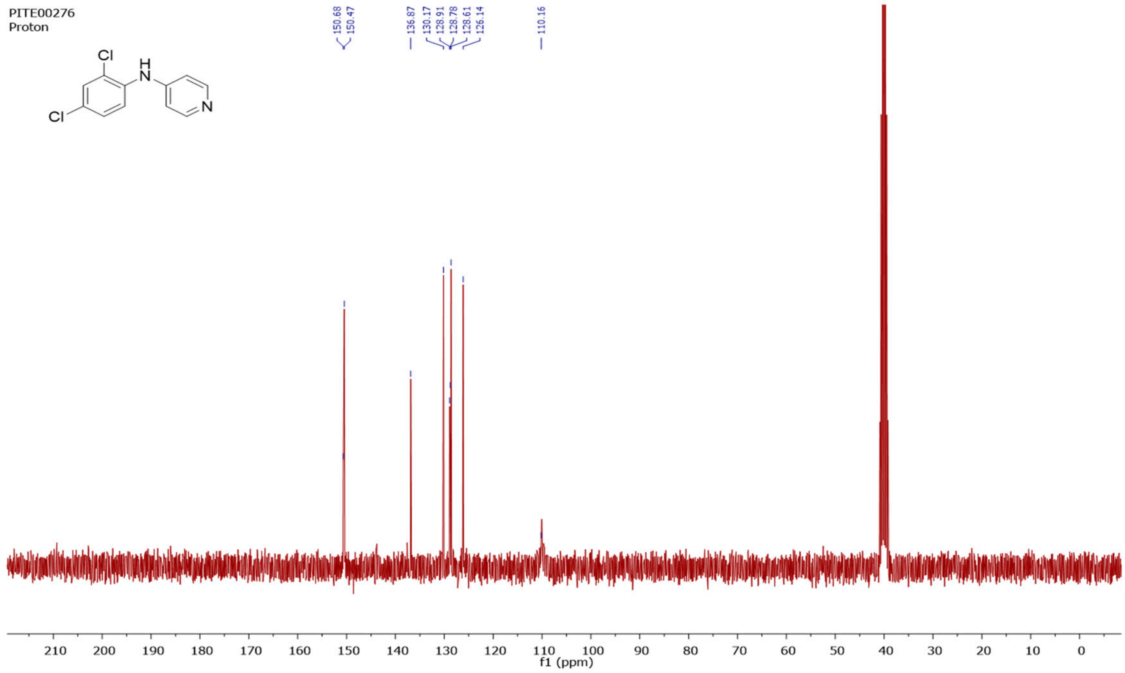

Figure S30. ^{13}C-NMR for *N*-(2,4-dichlorophenyl)pyridin-4-amine.

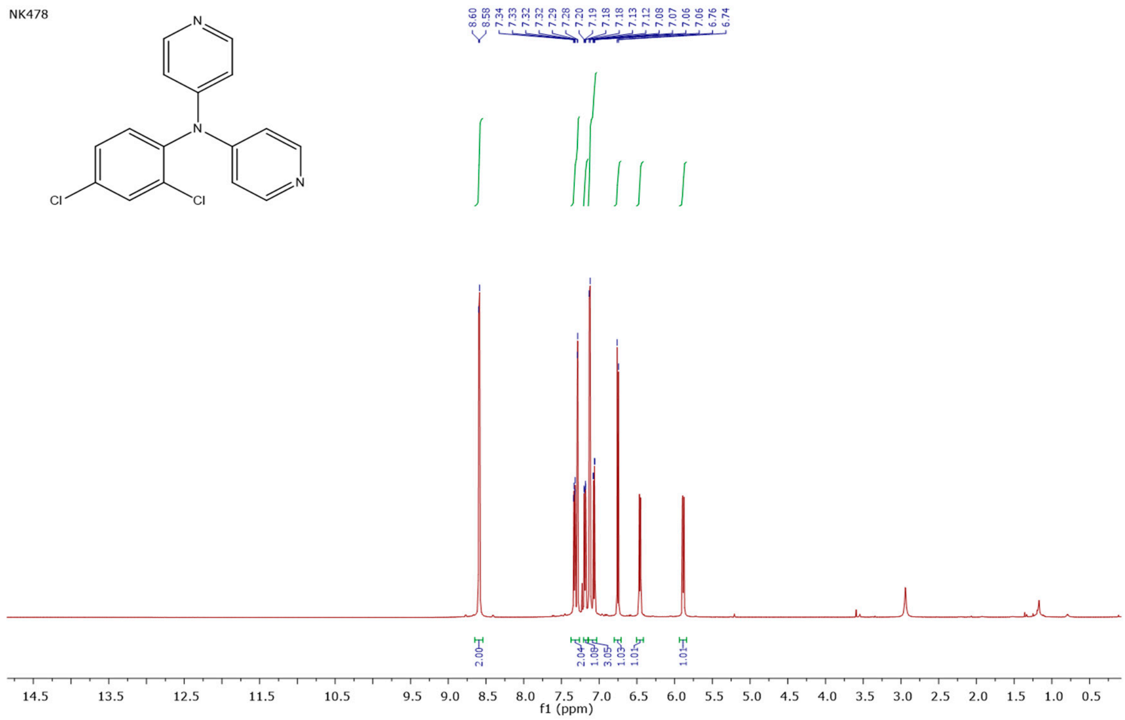

Figure S31. ^{1}H-NMR for *N*-(2,4-dichlorophenyl)-*N*-(pyridin-4-yl)pyridin-4-amine.

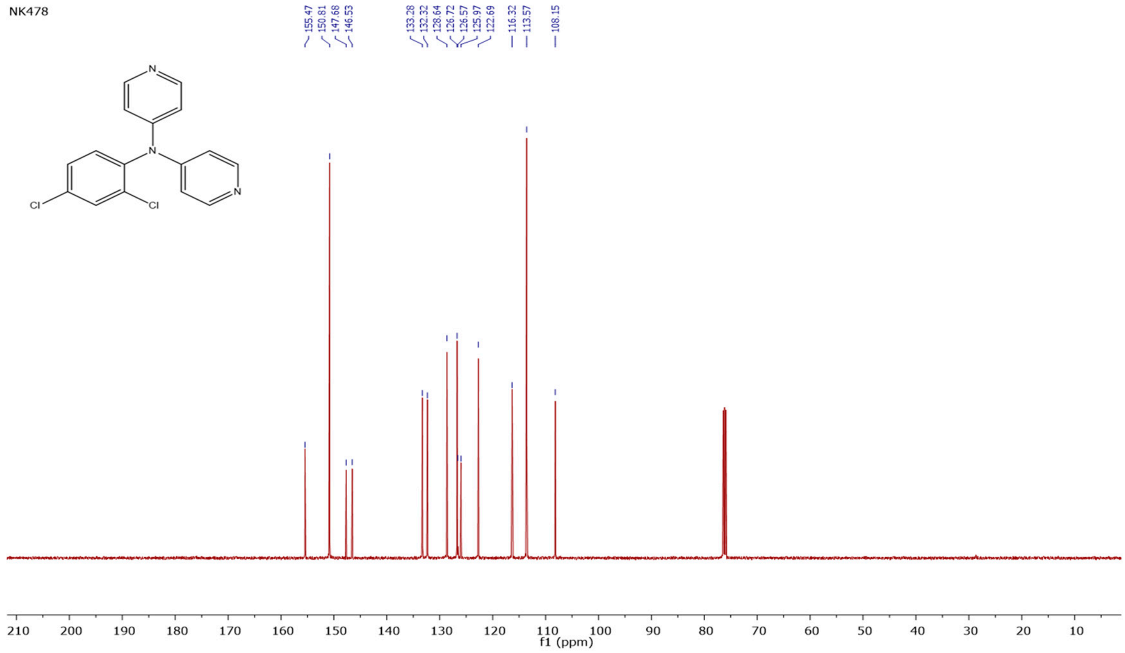

Figure S32. ^{13}C-NMR for *N*-(2,4-dichlorophenyl)-*N*-(pyridin-4-yl)pyridin-4-amine.

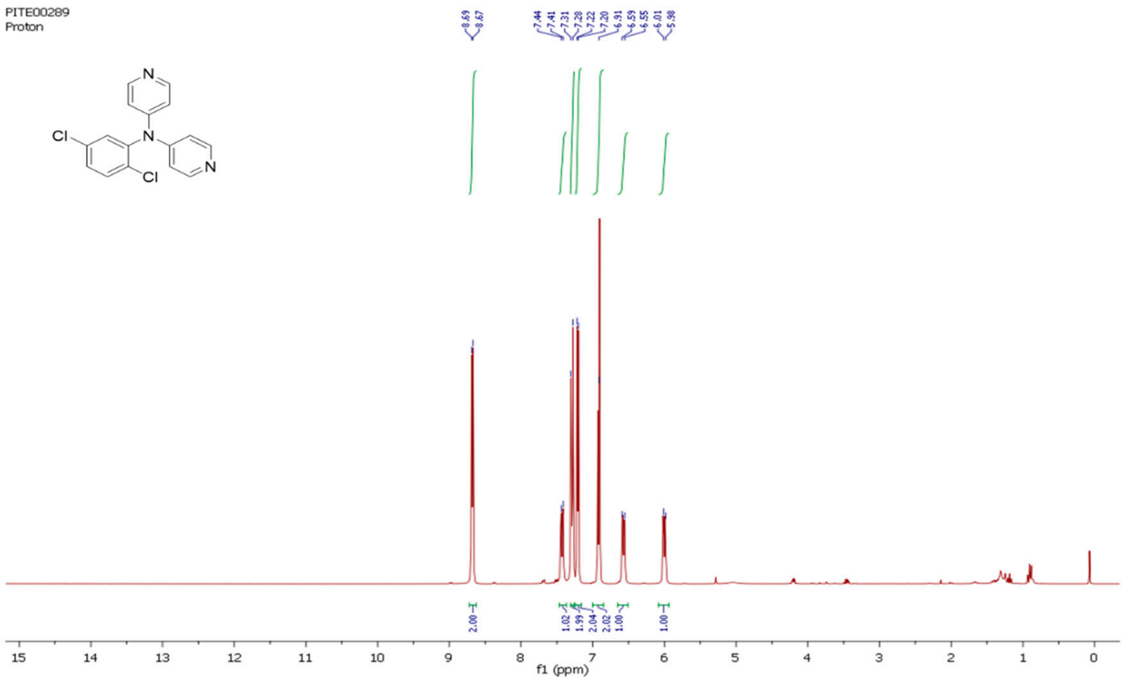

Figure S33. ^{1}H-NMR for *N*-(2,5-dichlorophenyl)-*N*-(pyridin-4-yl)pyridin-4-amine.

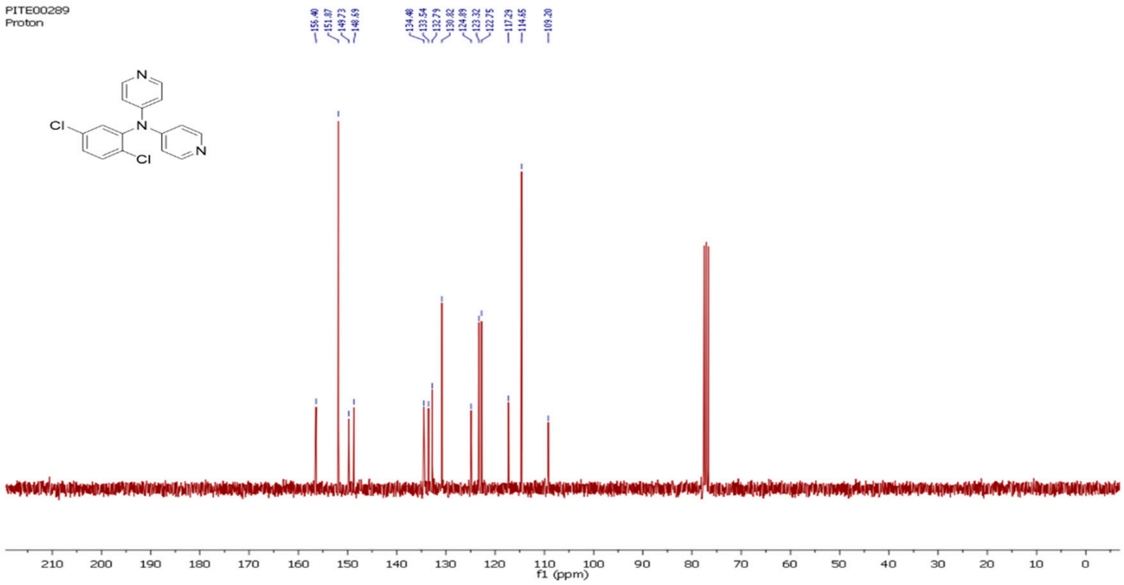

Figure S34. ^{13}C-NMR for *N*-(2,5-dichlorophenyl)-*N*-(pyridin-4-yl)pyridin-4-amine.

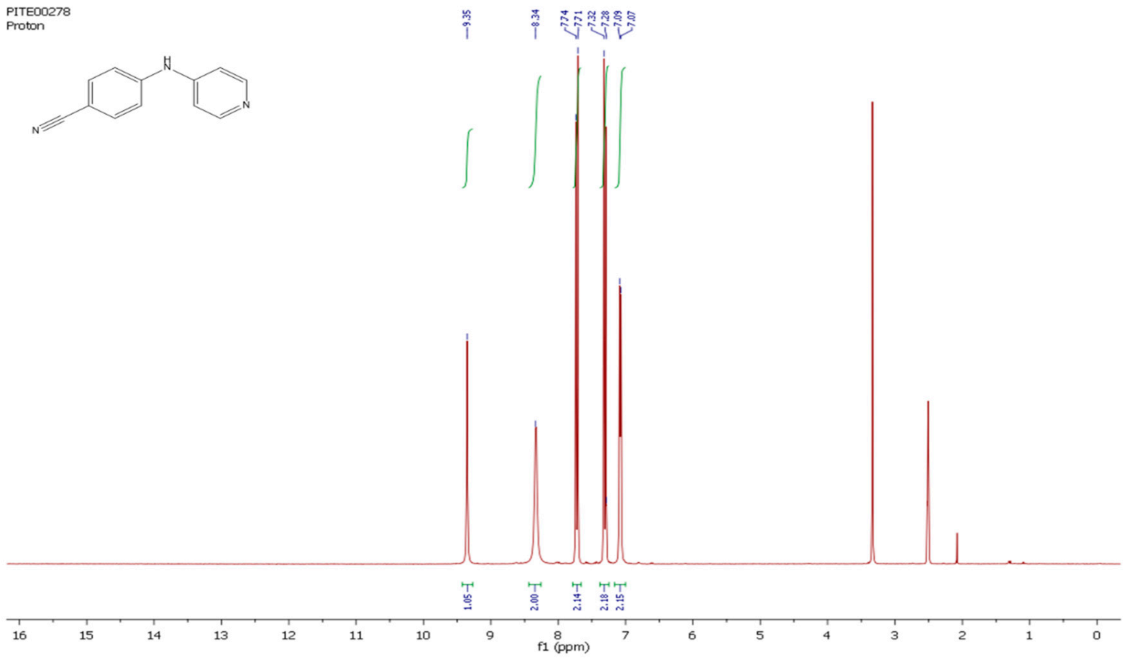

Figure S35. ^{1}H-NMR for 4-[(pyridin-4-yl)amino]benzonitrile.

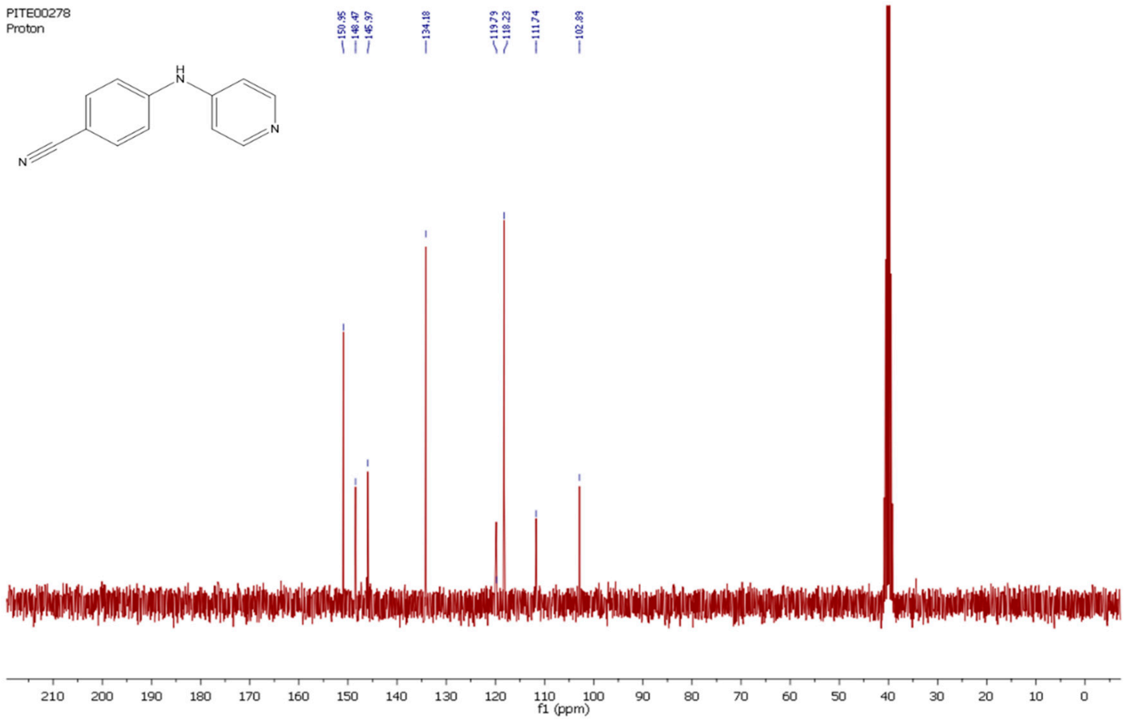

Figure S36. ^{13}C-NMR for 4-[(pyridin-4-yl)amino]benzonitrile.

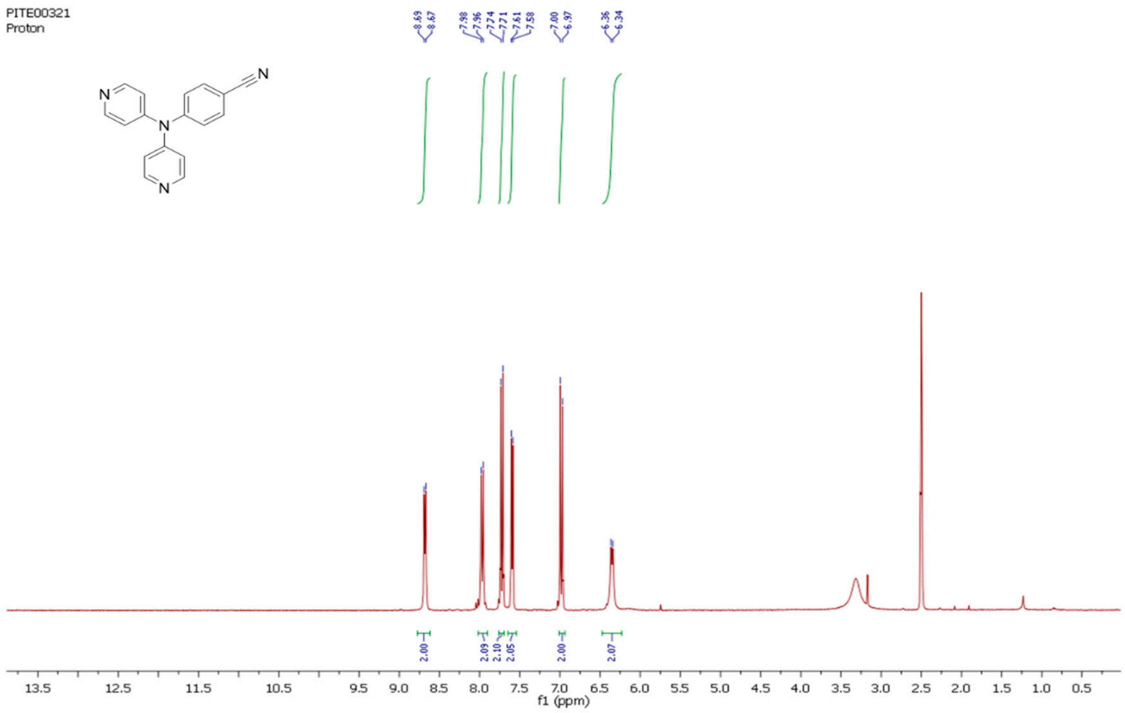

Figure S37. ^{1}H-NMR for 4-[(dipyridin-4-yl)amino]benzonitrile.

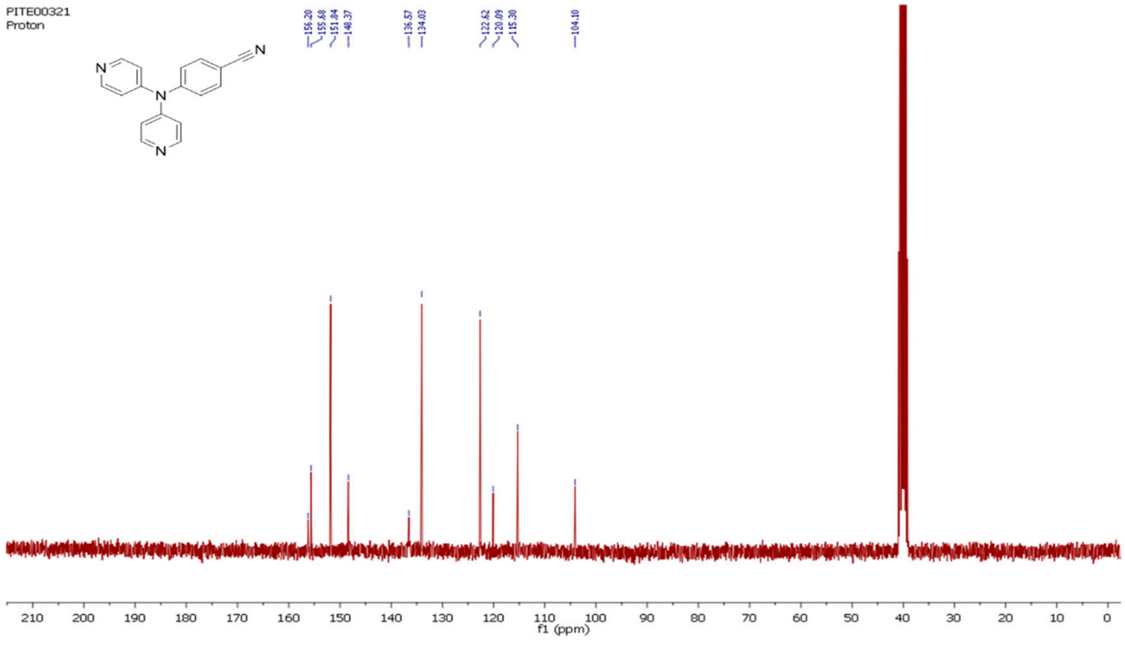

Figure S38. ^{13}C-NMR for 4-[(dipyridin-4-yl)amino]benzonitrile.

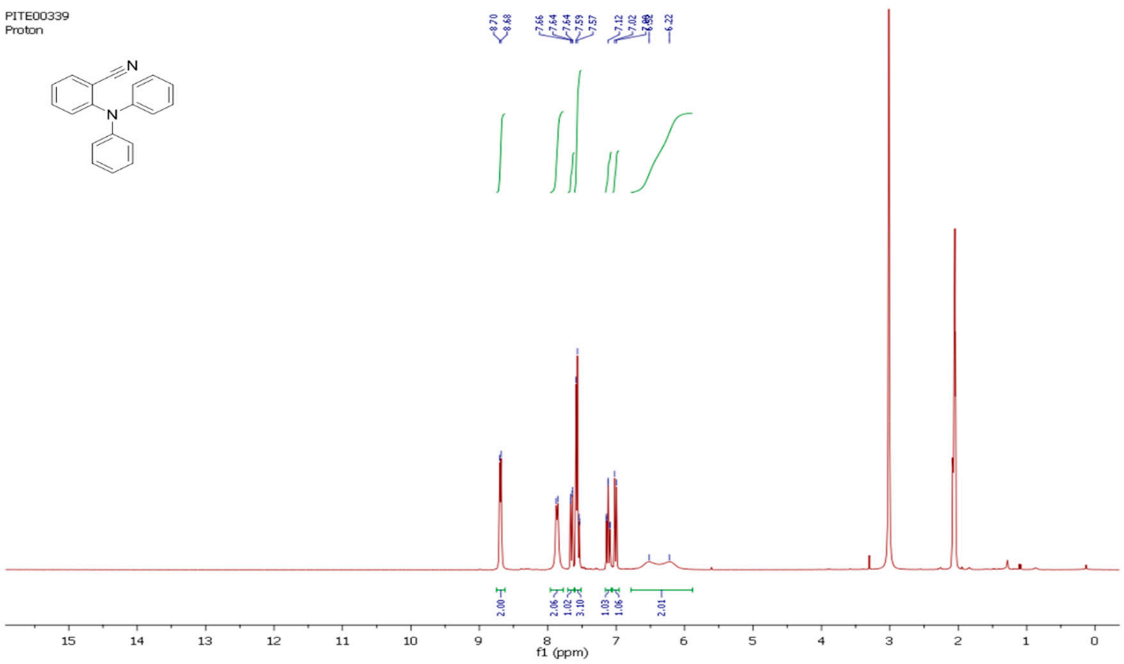

Figure S39. ^{1}H-NMR for 4-[di(pyridin-4-yl)amino]benzonitrile.

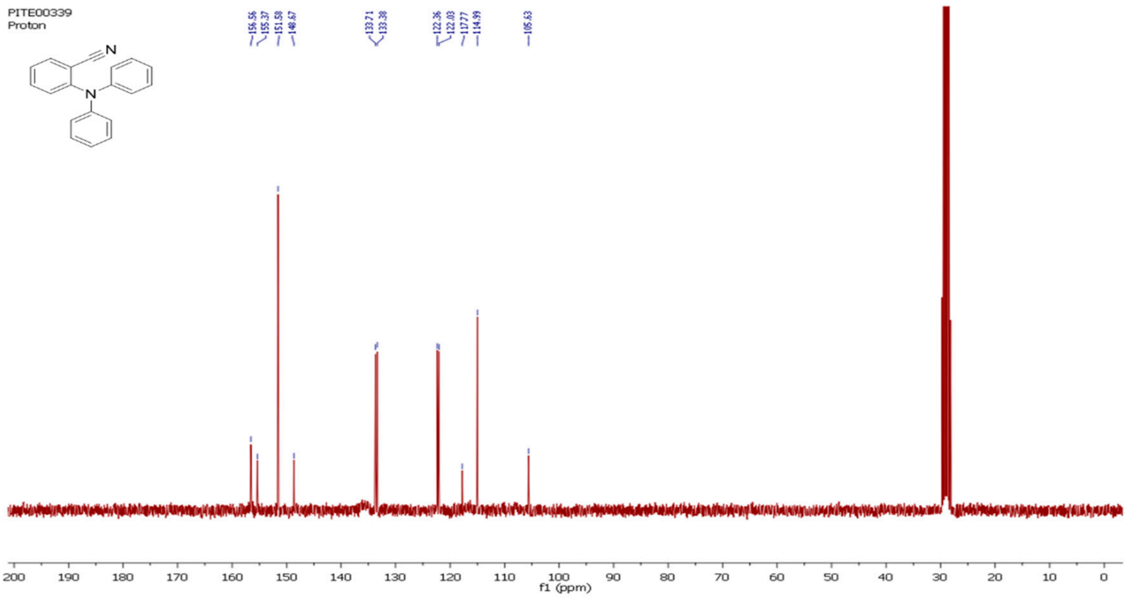

Figure S40. ^{13}C-NMR for 4-[di(pyridin-4-yl)amino]benzonitrile.

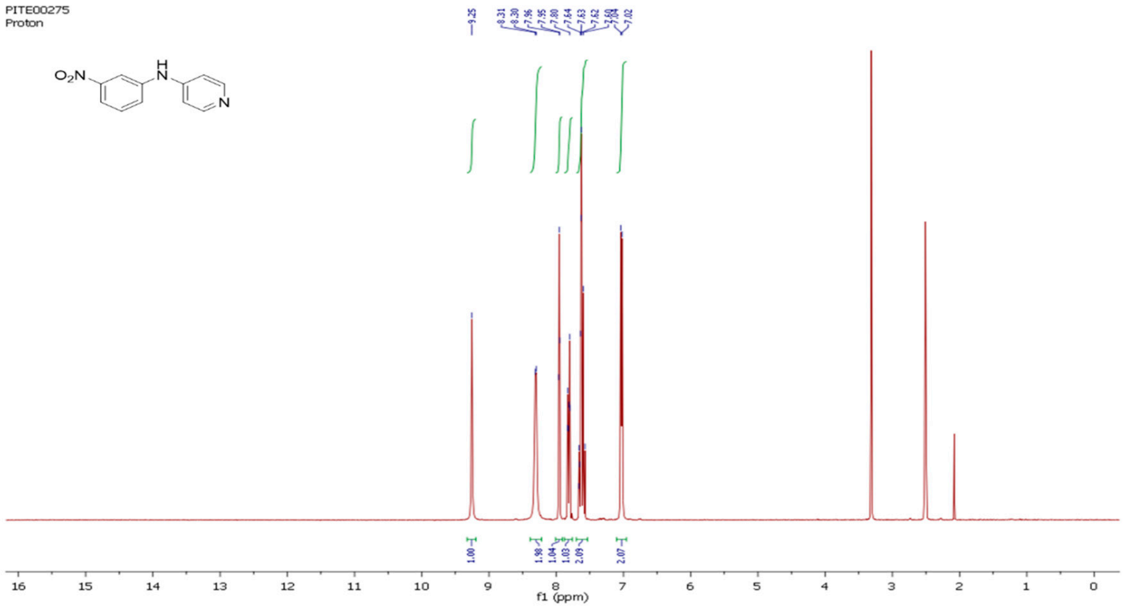

Figure S41. ^{1}H-NMR for *N*-(3-nitrophenyl)pyridin-4-amine.

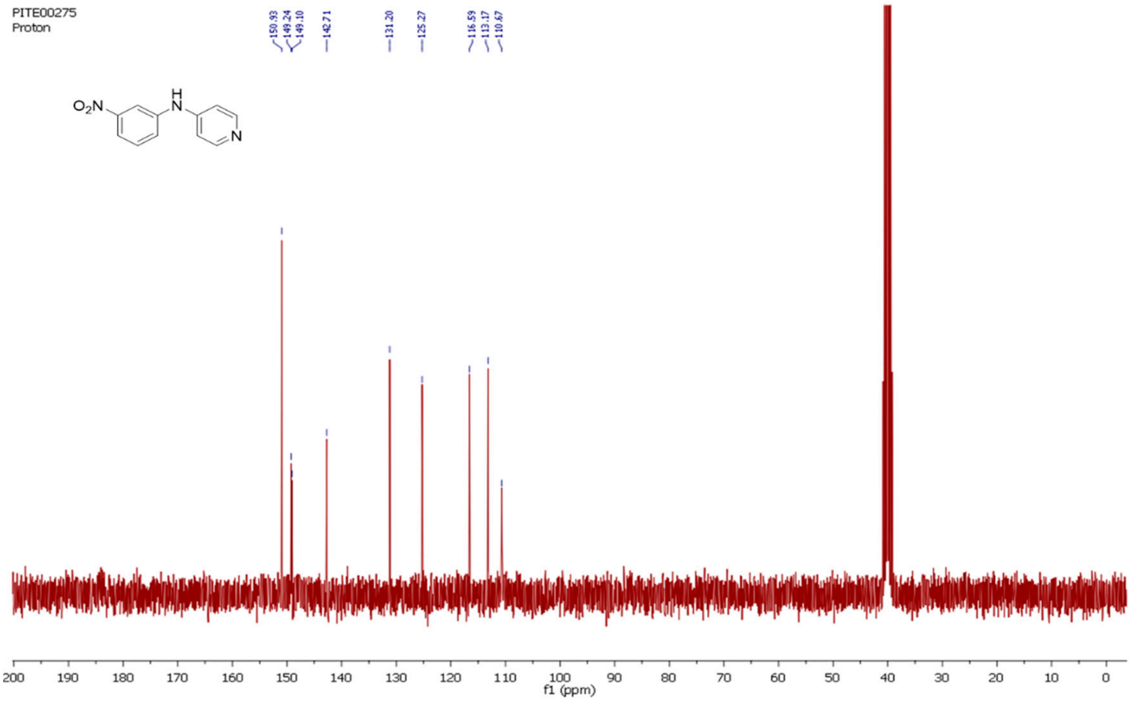

Figure S42. ^{13}C-NMR for *N*-(3-nitrophenyl)pyridin-4-amine.

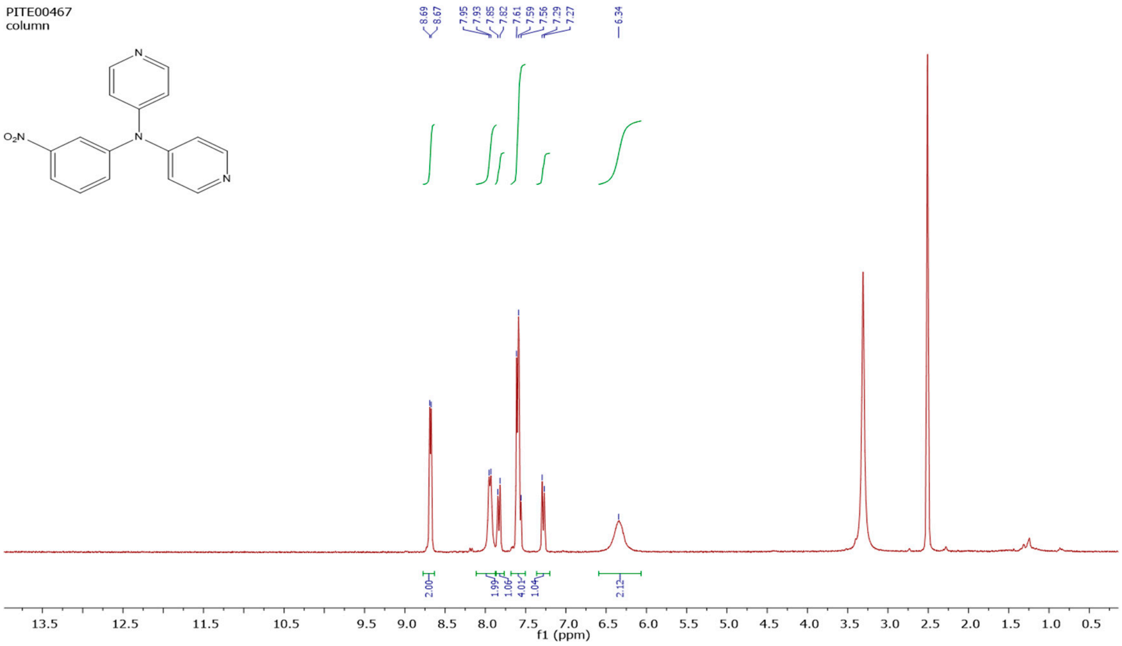

Figure S43. ^{1}H-NMR for *N*-(3-nitrophenyl)-*N*-(pyridin-4-yl)pyridin-4-amine.

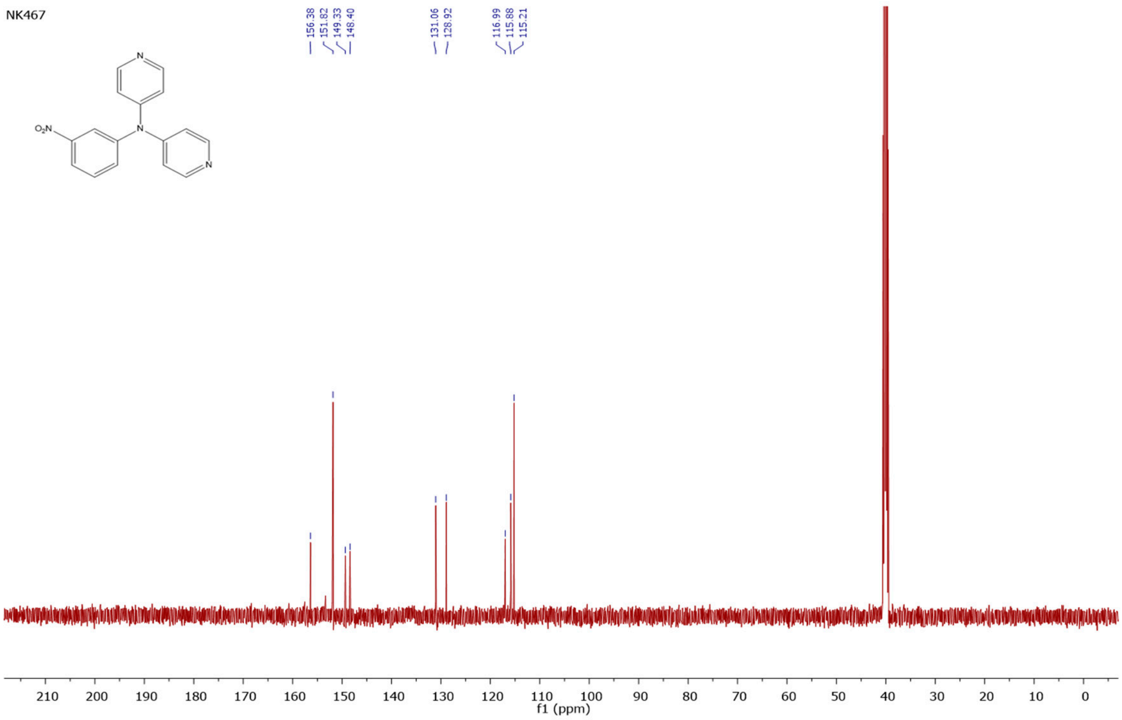

Figure S44. ^{13}C-NMR for *N*-(3-nitrophenyl)-*N*-(pyridin-4-yl)pyridin-4-amine.

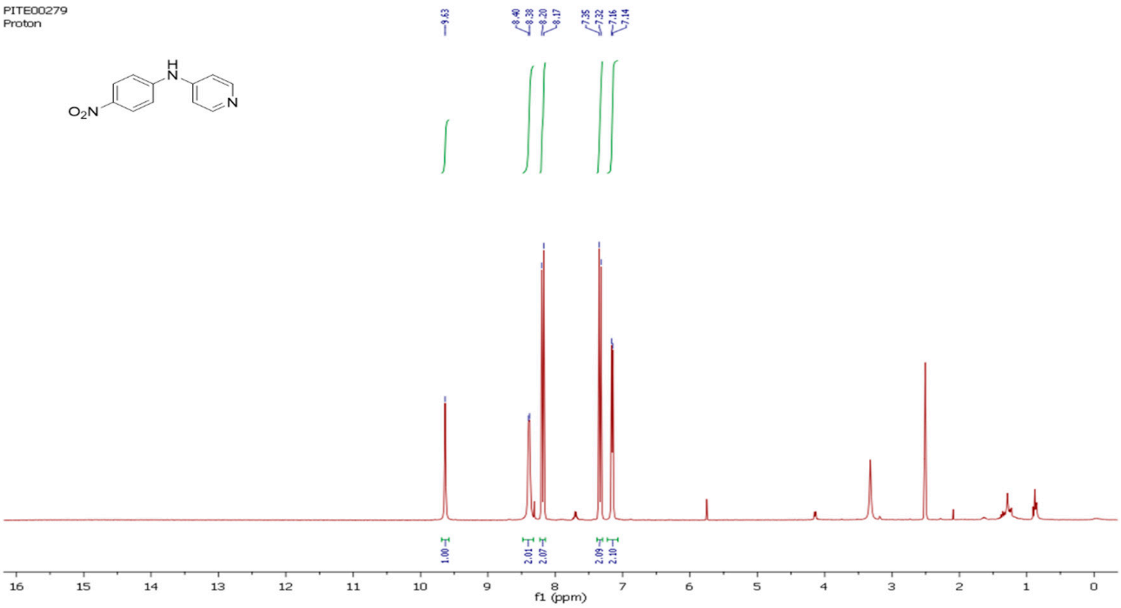

Figure S45. ^{1}H-NMR for *N*-(4-nitrophenyl)pyridin-4-amine.

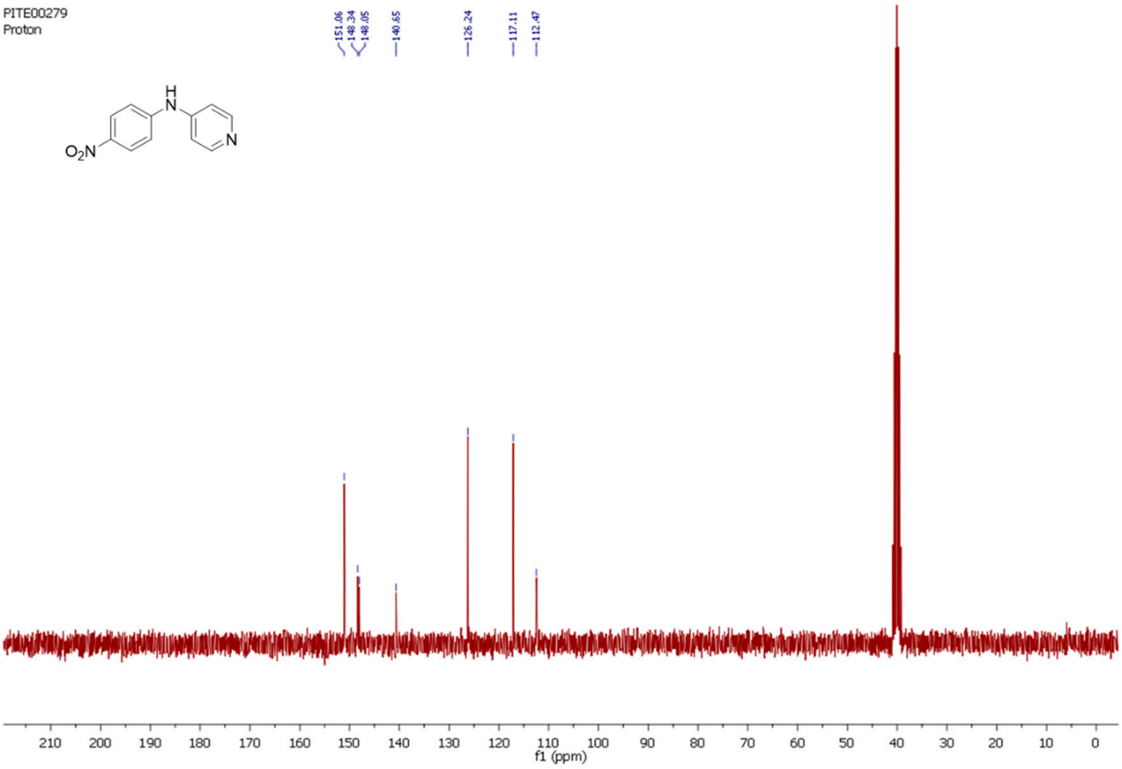

Figure S46. ^{13}C-NMR for *N*-(4-nitrophenyl)pyridin-4-amine.

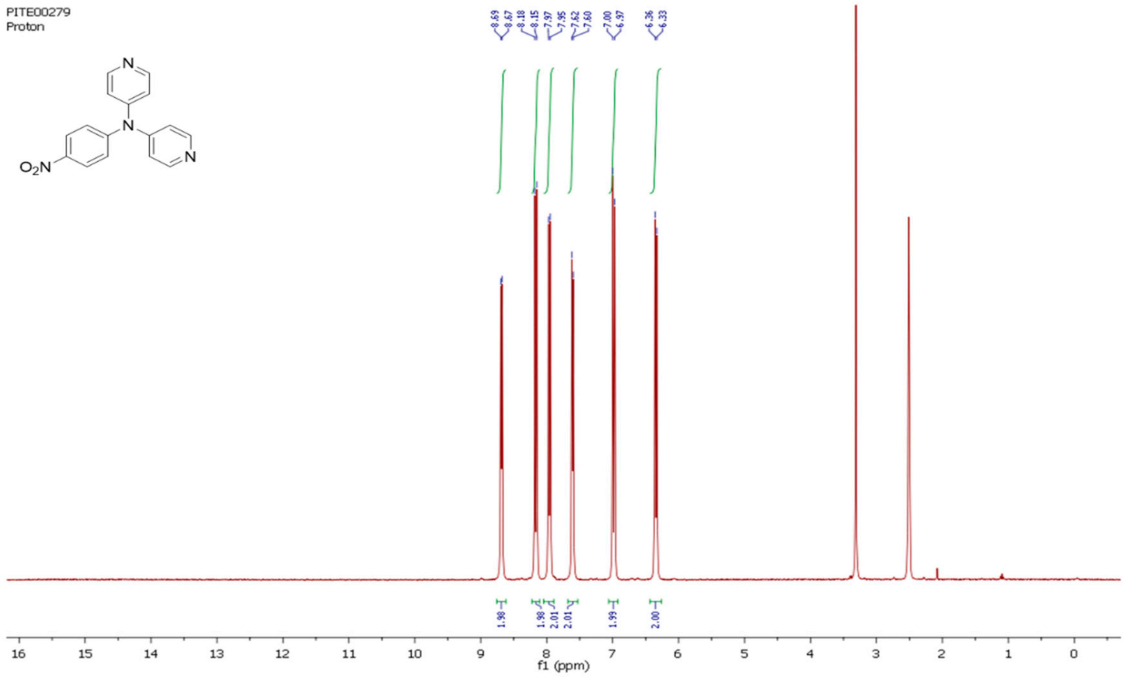

Figure S47. ^{1}H-NMR for *N*-(4-nitrophenyl)-*N*-(pyridin-4-yl)pyridin-4-amine.

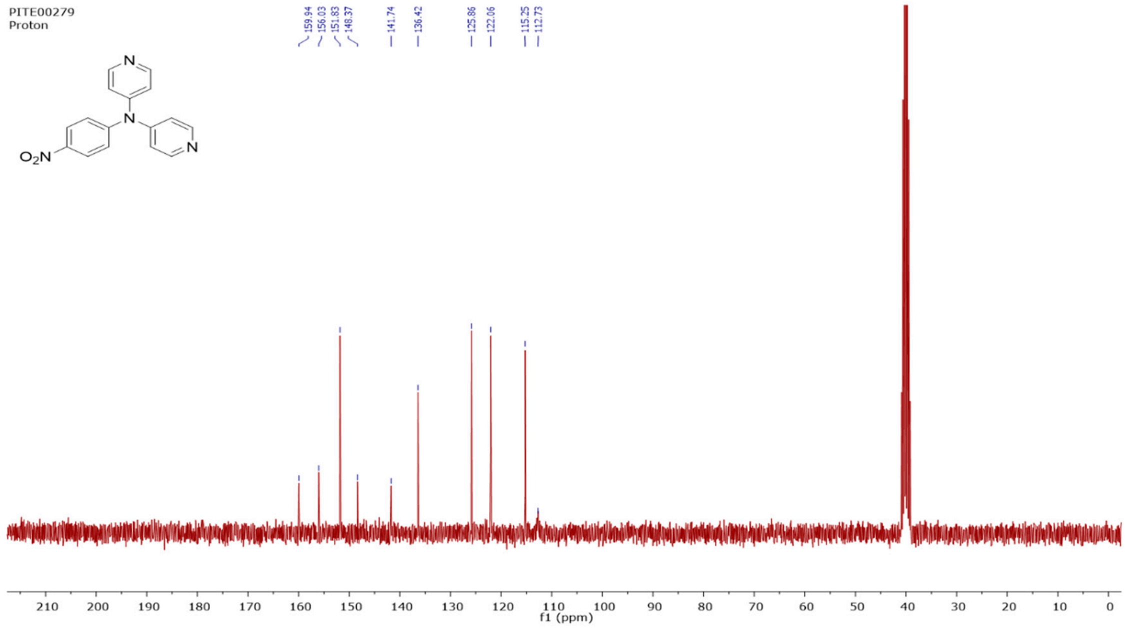

Figure S48. ^{13}C-NMR for *N*-(4-nitrophenyl)-*N*-(pyridin-4-yl)pyridin-4-amine.

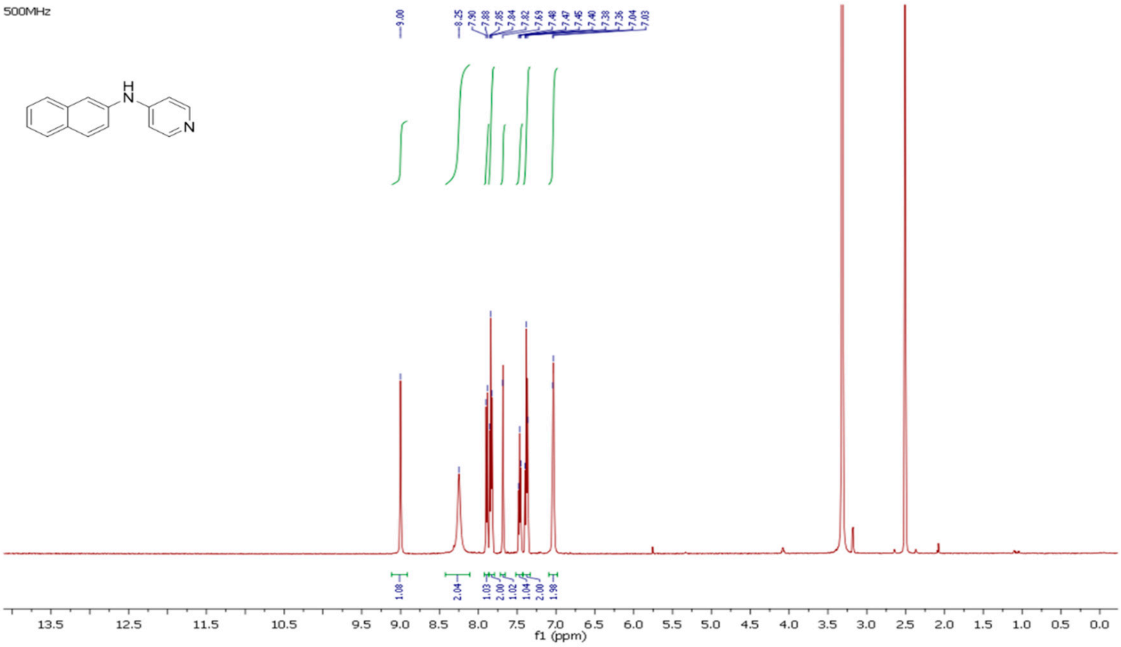

Figure S49. ^{1}H-NMR for *N*-(naphthalen-2-yl)pyridin-4-amine.

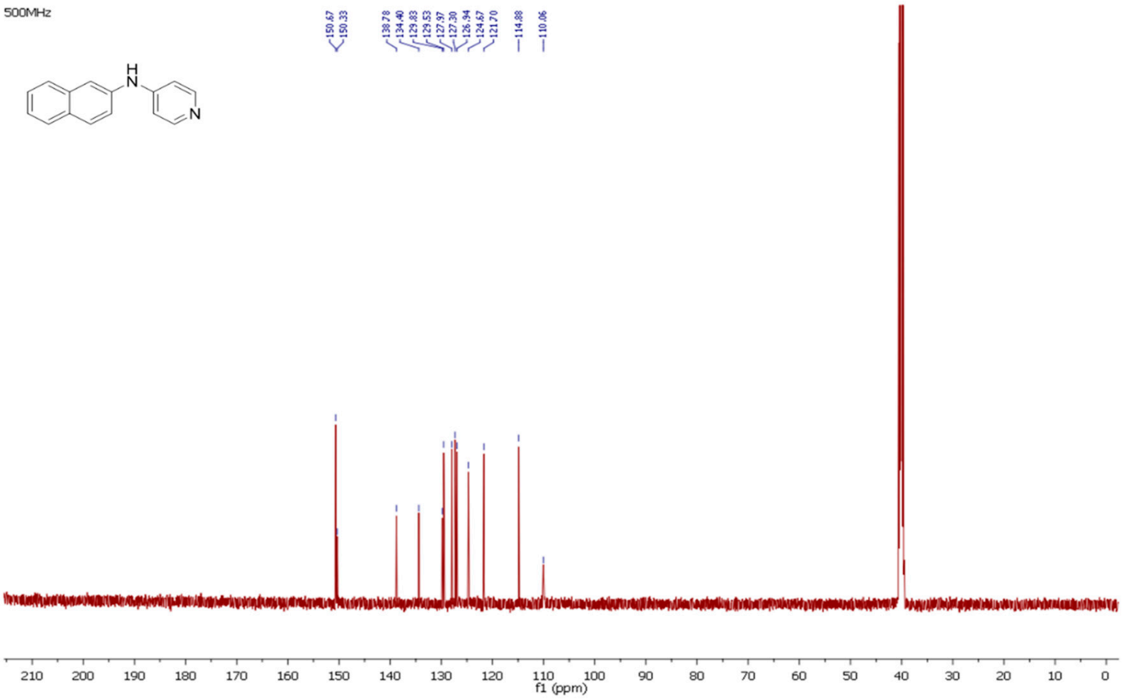

Figure S50. ^{13}C-NMR for *N*-(naphthalen-2-yl)pyridin-4-amine.

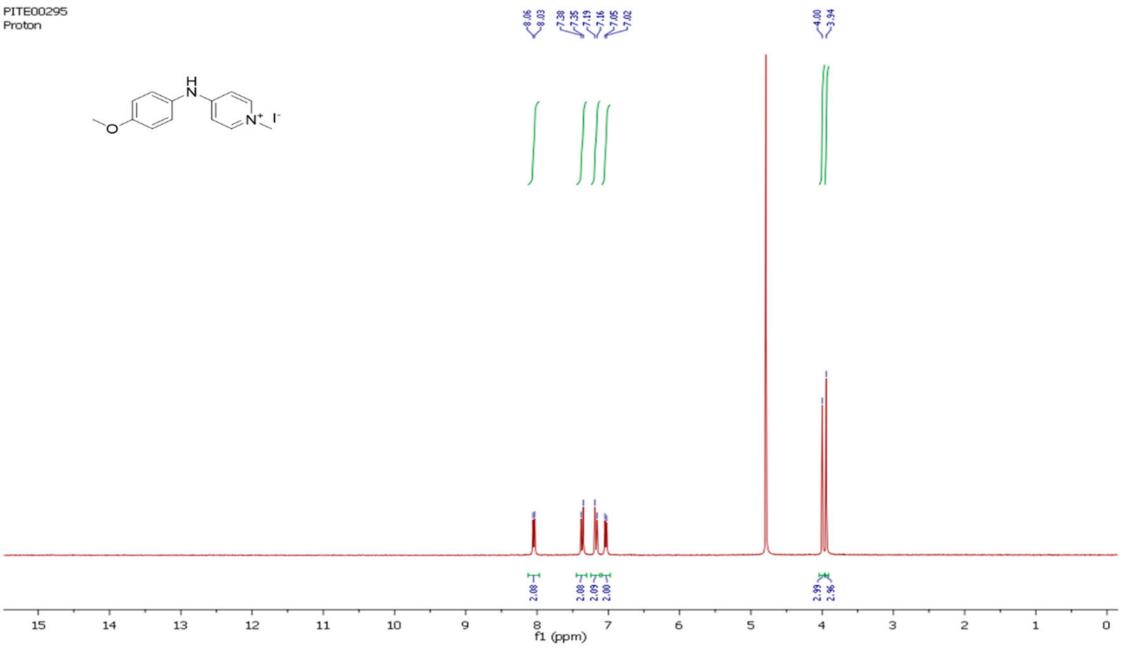

Figure S51. ^{1}H-NMR for 4-(4-methoxyphenylamino)-1-methylpyridin-1-ium iodide.

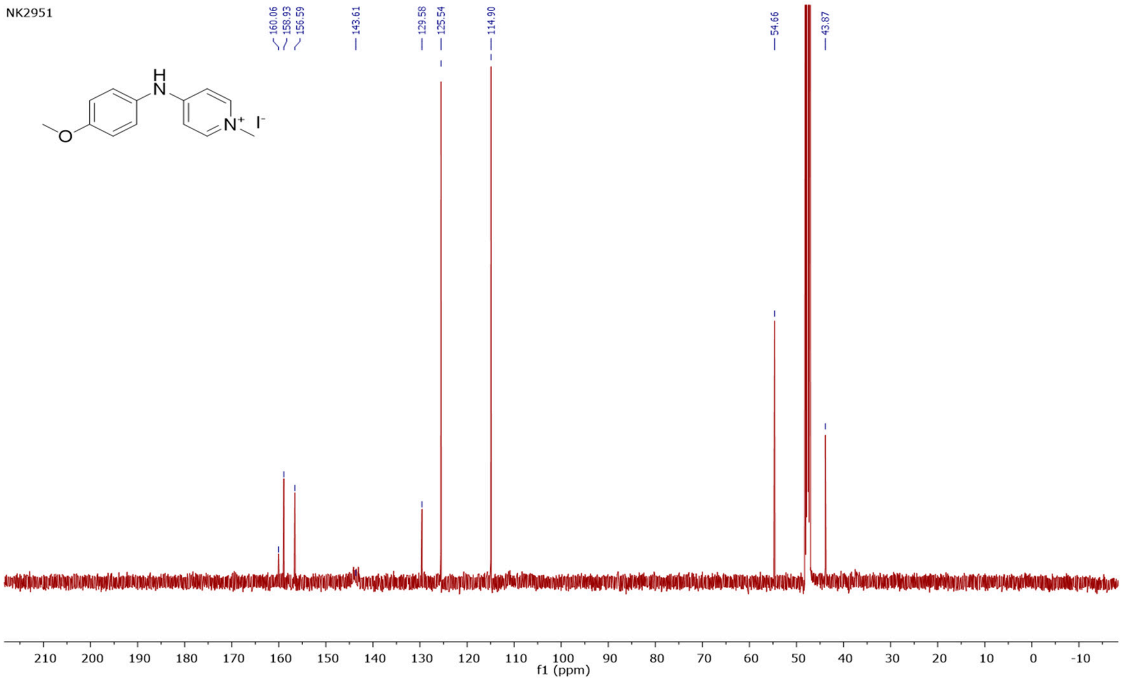

Figure S52. ^{13}C-NMR for 4-(4-methoxyphenylamino)-1-methylpyridin-1-ium iodide.

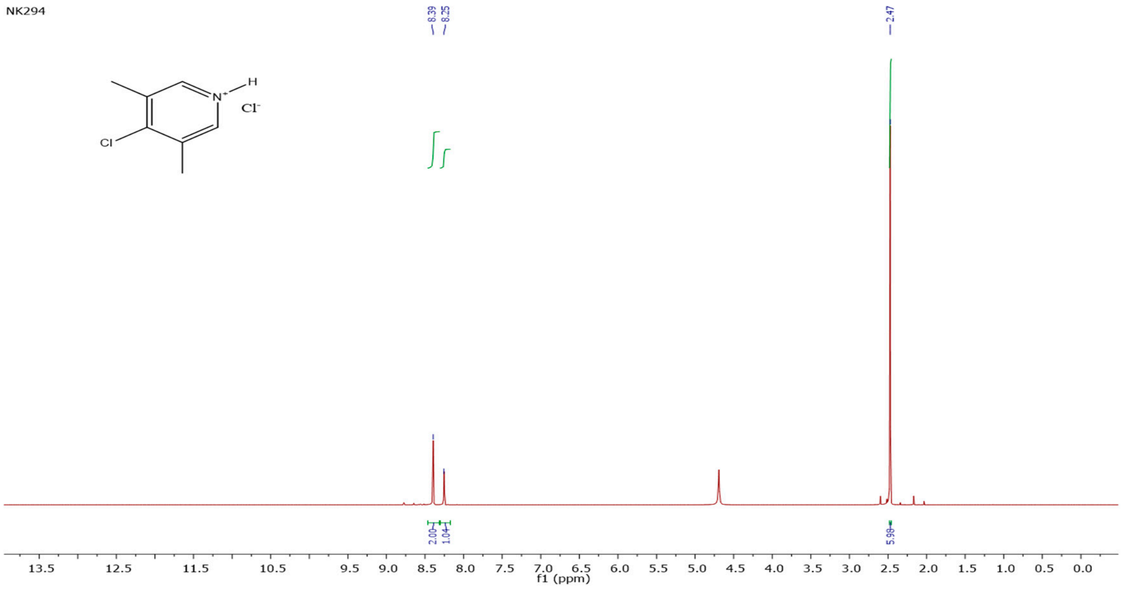

Figure S53. ^{1}H-NMR for chloro-3,5-dimethylpyridin-1-ium chloride.

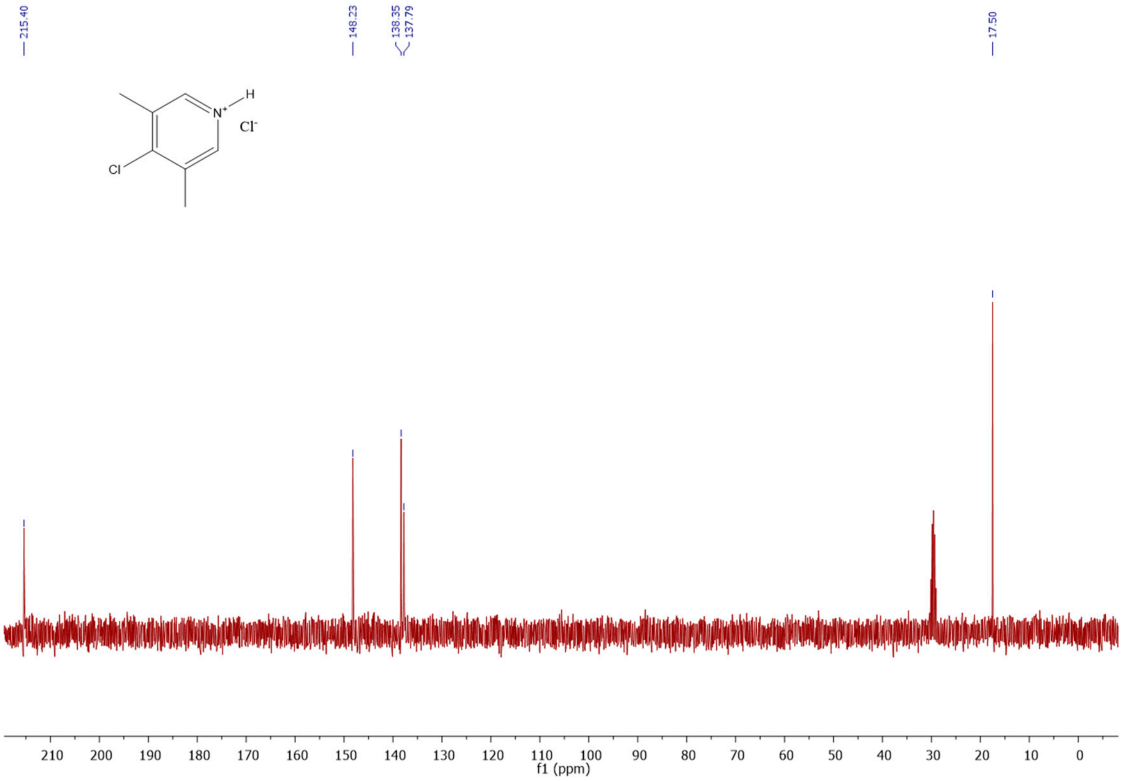

Figure S54. ^{13}C-NMR for chloro-3,5-dimethylpyridin-1-ium chloride.

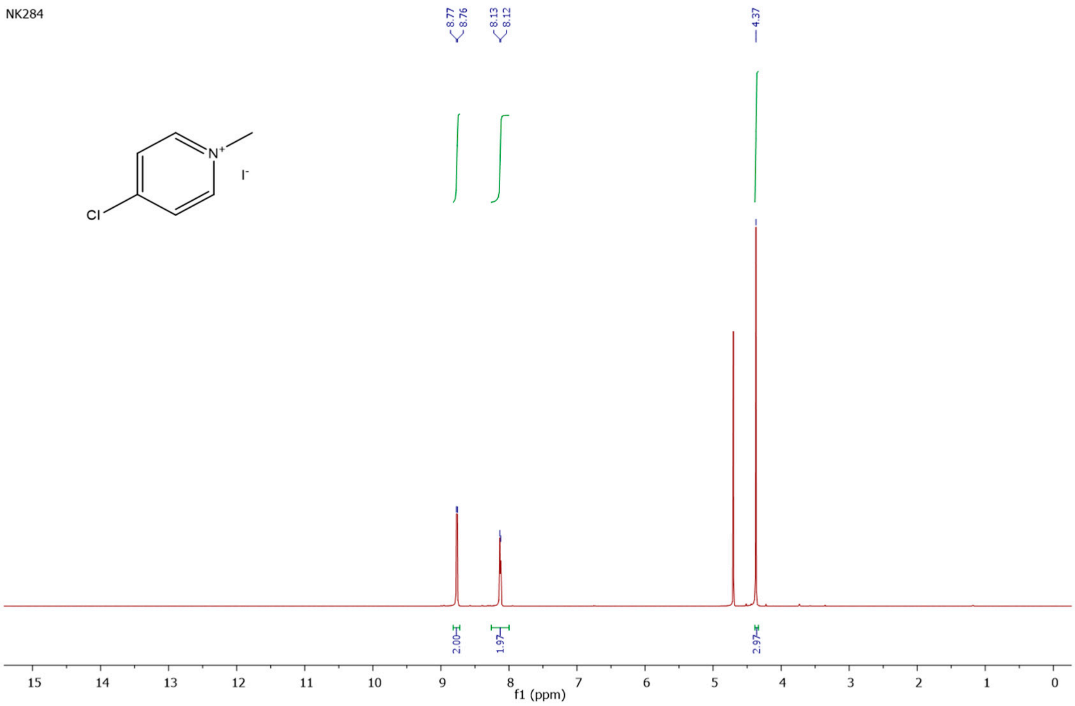

Figure S55. ^{1}H-NMR for 4-chloro-1-methylpyridin-1-ium iodide.

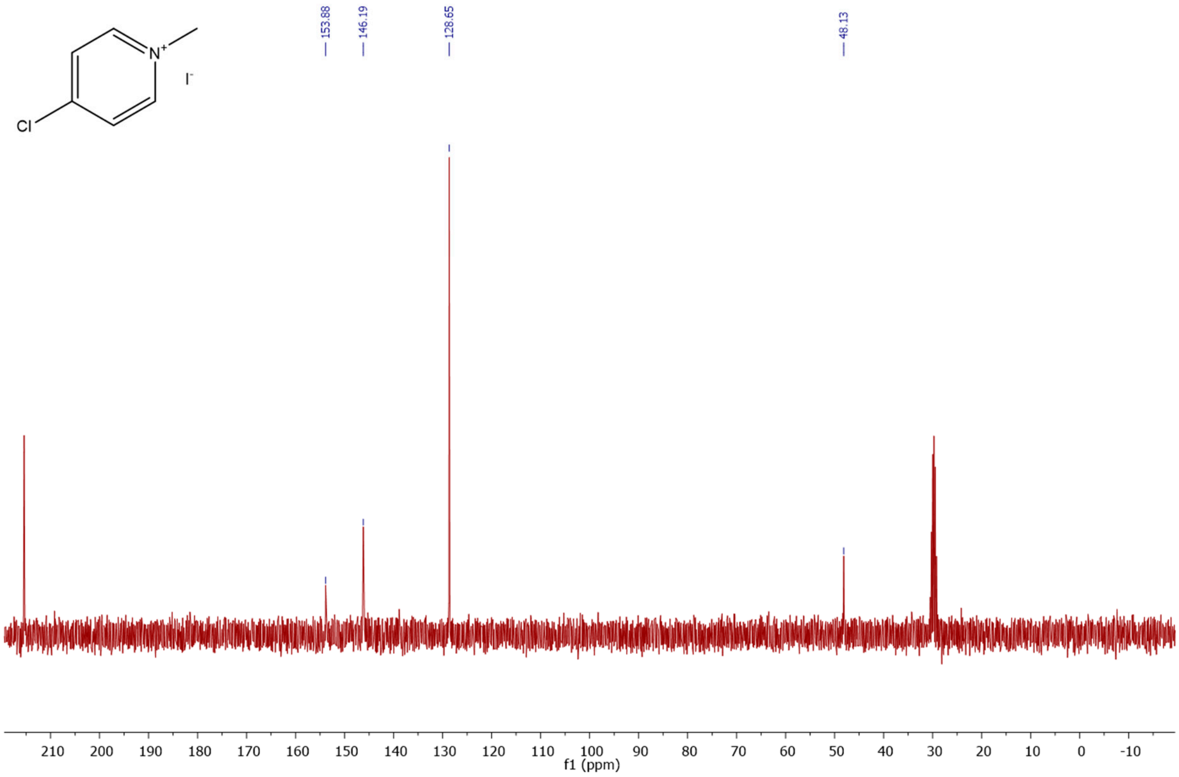

Figure S56. ^{13}C-NMR for 4-chloro-1-methylpyridin-1-ium iodide.

References

1. Zoghbi, M.; Chen, L. Synthesis of Pharmaceutically Useful Pyridine Derivatives. US 6437139, 20 August 2002.
2. Souza Brenelli, E.C.; Samenho Moran, P.J. The reactivity of some primary amines in SN2Ar reactions with 2- and 4-chloro-1-methylpyridinium ions. *J. Chem. Soc. Perkin Trans.* **1989**, *2*, 1219–1222.
3. Jerchel, D.; Jakob, L. *N*-Pyridyl-aminophenole aus pyridyl-[amino-phenyl]-äthern; eine neue Umlagerungsreaktion. *Chem. Ber.* **1959**, *92*, 724–732.
4. Keddie, D.J.; Guerrero-Sanchez, C.; Moad, G.; Mulder, R.J.; Rizzardo, E.; Thang, S.H. Switchable reversible addition fragmentation chain transfer (raft) polymerization in aqueous solution, *N*,*N*-dimethylacrylamide. *Macromolecules* **2012**, *45* 4205–4215.
5. Katritzky, A.R. The preparation of some substituted pyridine 1-oxides. *J. Chem. Soc.* **1956**, 2404–2408.
6. Bailey, D.M.; DeGaris, C.G.; Hoff, S.J.; Schulenburg, P.L.; O'Connor, J.R.; Paris D.A.; Sleet, A.M. Bispyridinamines: A new class of topical antimicrobial agents as inhibitors of dental plaque. *J. Med. Chem.* **1984**, *27*, 1457–1464.
7. Abou-Shehada, S.; Teasdale, S.D.; Bull, S.D.; Wade, C.E.; Williams, J.M.J. Lewis acid activation of pyridines for nucleophilic aromatic substitution, and conjugate addition. *ChemSusChem*, **2015**, *8*, 1083–1087.
8. Sammes, M.P.; King-Wah, H.; Tam, M.L.J. Synthetic applications of N-N linked heterocycles. Part 15. A facile synthesis of 4-pyridyl(aryl)amines via the reaction between 4-chloro-1-pyridiniopyridinium salts and aryl amines. *Chem. Soc. Perkin Trans. 1* **1983**, *5*, 973–978.
9. Schulte, J.P.; Tweedie, S.R. Palladium-catalyzed couplings of heteroaryl amines with aryl halides using sodium phenolate as the stoichiometric base. *Synlett* **2007**, *15*, 2331–2336.
10. Bearss, D.; Brooke, D.; Denny, W.; Gamage, S.; Phiasivongsa, P.; Redkar, S.; Supergen, I.; Vankayalapati, H. Quinoline Derivatives for Modulating Dna Methylation, WO002008046085A2, 17 April. 2008.
11. Peters, A.T. Synthesis, and ultraviolet spectra of nitroanilinopyridines. *J. Soc. Dyers Colour.* **1970**, *86*, 77–79.
12. Grout R.J.; Partridge, M.W.; Sprake, J.M.; Vipond, H.J. Polyazabenzo[a]pyrenes. *J. Chem. Soc. C* **1968**, 2689-2693, doi: 10.1039/J39680002689.
13. Mary, Y.S.; Panicker, C.Y.; Varghese, H.T.; Alsenoy, C.V.; Procházková, M.; Ševcík, R.; Pazdera, P. Acid–base properties, FT-IR, FT-Raman spectroscopy and computational study of 1-(pyrid-4-yl)piperazine. *Spectrochim. Acta Part A Mol. Biomol. Spectrosc.* **2014**, *121*, 436–444.
